# Women's perception about contraceptive use benefits towards empowerment: A phenomenological study in Southern Ethiopia

**DOI:** 10.1371/journal.pone.0203432

**Published:** 2018-09-13

**Authors:** Abraham Alano, Lori Hanson

**Affiliations:** 1 School of Public Health, College of Medicine and Health Sciences, Hawassa University, SNNPR, Ethiopia; 2 Department of Community Health and Epidemiology, College of Medicine, University of Saskatchewan, Saskatoon, Canada; ESIC Medical College & PGIMSR, INDIA

## Abstract

**Background:**

Despite the availability of copious information regarding contraceptive use benefits and the factors that influence the uptake of the services, there is little evidence revealing the lived experiences of rural women. Thus, this study was conducted with the purpose of exploring the lived experiences of women regarding contraceptive use and related benefits towards women’s empowerment.

**Methods:**

Interpretative phenomenological qualitative methodology was employed to explore the lived experiences of women. Data were collected through focus group discussions and in-depth individual interviews and analyzed using an interpretive phenomenological framework including phases of data immersion, transcribing, coding, theme development and phenomenological interpretation through hermeneutic circle.

**Result:**

The reported lived experiences of rural women revealed that their livelihoods greatly improved in different ways after they began to use contraceptives. The benefits included securing more time, energy and social engagements. Contraceptive use helped women postpone unwanted pregnancies and child births and engage in various income generation activities that not only boosted family incomes but also created opportunity to mobilize the resources for different expenses without waiting for the handouts from their husbands. The women’s experiences also indicated that contraceptive use improved the educational status of their daughters and they experienced improved self-image, better social standing and improved family relations. The experiences further illustrated that contraceptive use was not only emancipatory and transformative, but also created peace and stability in their lives.

**Conclusion:**

The study concludes that contraceptive use, which is part of a woman’s life experience, created remarkable opportunities and achievements. One of these was that women were able to control their bodies, reproduction and fertility which resulted in a higher degree of empowerment. The control of reproduction and fertility has liberated them from worries and entrapment of unplanned and unwanted pregnancies. Moreover, contraceptive use led to wider opportunities in the community, by improving their status and building a sense of empowerment. Creating awareness around the benefits of contraceptive use has the potential to improve community and national development. Based on the result, the study recommends that systems should be established to capitalize on the lessons learned about the lives of current users and expand the remarkable achievements and experiences to non-user counterparts.

## Introduction

The use of modern contraceptive methods was documented in the early 1960s (Cleland, 2008), albeit with varying levels of utilization around the world [1–3]. The innovative development of safe and effective contraceptive methods has critically benefited humankind in numerous ways [4–6]. The outcomes of contraceptives use include; poverty reduction, reduction of maternal and child mortality, women empowerment by reducing the burden of excess childbearing, and enhancement of environmental sustainability by stabilizing the population of the planet [5, 7–10]

The link between reproductive rights and women’s empowerment and the role of contraceptive utilization is obvious [11]. The ability of women to control their sexuality and fertility through proper use of contraceptives is the cornerstone to ensure other aspects of women’s rights and human rights [12, 13]. Contraception offers remarkable contributions towards the empowerment of women in multiple ways, including the avoidance of unplanned and unwanted pregnancies, increasing the amount of time between successive pregnancies, and enabling engagement in educational and economically productive activities [5, 14].

Despite the observed improvement in contraceptive prevalence rate (CPR) in Ethiopia currently, there is variation across the regions. The great variation in CPR is observed among urban and rural, pastoral and non-pastoral, and region to region. CPR in Addis Ababa is as high as 63% (nearly the global average) and in some pastoral regions as low as 10% [15]. The CPR in the study area is 25% [15]. In a similar manner, political and administrative trends in Ethiopia also created power differences between women and men, denying the right and privileges of women (although the current government has clearly attempted to change that through the 1995 constitution). Many additional circumstances affect the status of women and girls, and gender equality between women and men. These factors have been contributing to the difference in contraceptive utilization across the country from region to region, urban to rural dweller, and educated versus uneducated [16–20]. Moreover, lack of comprehensive knowledge about the benefits of contraceptives to the wider domains of women’s lives, inadequate support from the husband/spouse, low service quality, and limited types of contraceptive methods affect contraceptive usage rates [21].^.^ Notably, Ethiopia is still suffering from preventable morbidity and mortality of mothers and newborns with one of the highest maternal mortality rates (420 per 100,000 live births) and neonatal mortality rates (of 38 per 1000 live births), as well as high total fertility rates (4.6 children per woman), and the highest unmet need for family planning (26 percent) in the world [15, 22].

Despite some encouraging efforts and recent progress in improving access to reproductive health services, including contraceptive methods provisions, Ethiopia remains at lower levels with wide gaps and lags behind in service provision to reach the desired level of contraceptive acceptance, fertility, and accompanying health and empowerment benefits for women.

An important observation discovered in addressing these challenges is that in similar socio-economic, cultural and environmental contexts, some women appear to adopt modern contraceptives while others do not. This trend occurs in almost every part of the Ethiopia where contraceptive services are available [15, 23]. Yet, there is little evidence that explains why. The evidence is also elusive regarding how family planning service provision is perceived by the primary beneficiaries (the rural women), whether and how they relate it towards their empowerment, and how it is valued and internalized by them in order to ensure sustained use.

This study was conducted with the purpose of understanding the perceptions regarding women’s contraceptive use and the subsequent benefits towards their empowerment. In addition, the study aimed to narrow the gap between current users and non-users, and focused on women with unmet needs by sharing the experiences of current users and their perceptions regarding contraceptive benefits, all while elucidating factors contributing to sustained contraceptive use.

## Materials and methods

### The research context

This study was conducted in three of six districts of Sidama Zone designated by Hawassa University as technology villages for research and technology transfer. These three districts were selected conveniently. The university is situated within this administrative zone and has revitalized its research and community services in order to materialize its contribution to the surrounding communities and ensure its transformation plan. Sidama Zone is one of the thirteen zones in the Southern Nations Nationalities and People’s Regional state (SNNPRG), under the Federal Democratic Government of Ethiopia. Sidama Zone is located in the south-eastern part of the region and is bordered on the south, east and north by the Oromia Region on the west; it borders Wolaita Zone [24]. According to the population projection based on the 2007 national population census, the zone has a total of 3,471,568 people of which 1,753,142 (50.5 percent) are males and 1,718,426 (49.5 percent) are females. Women of reproductive age are estimated to be 23.8 percent of the total population. Household population size is estimated to be 4.7. Annual population increase is estimated to be 2.9percent [25].

### Study design

#### Interpretive phenomenological qualitative research approach

The study was based on the interpretive (hermeneutic) approach appropriate to understand the life world of women, as it focuses on describing the meaning of the individuals and how these meanings, such as the experience of contraceptive use, the phenomenon influences the choices they make, rather than seeking purely descriptive categories of the real, perceived world in narratives of the participants [26]. It further considers the importance of the expert knowledge of the researcher as a valuable guide to the enquiry. Exploring the life experience related to contraceptive use by employing this approach, clearly offered a unique opportunity to establish a rich and in-depth understanding about the contribution of contraceptives towards the empowerment of women, the improvement of the health of women and their children, and the society at large [27].

#### Data collection

The study employed linguistically competent research assistants and utilized two main data collection methods. In order to capture in-depth information in relation to the topic of interest: focus group discussions (FGDs) and the individual in-depth interviews were used. Research assistants were recruited based on the selection criteria stated in the original thesis document. After recruiting and training the research assistants, health extension workers and local women, the community leaders collaborated in the selection of study participants. The participants were selected based on the following criteria: 1) women who could illustrate the phenomenon, contraceptive use and related benefits in the study kebeles (the smallest administrative unit) 2) women of the reproductive age group using any type of modern contraceptive method before and during the study period 3) women having used contraceptives for at least one year. The following strategies were used to select participants: 1) potential participants (women using contraceptive method) were approached by the community leaders and informed about purposes of the study, 2) names of interested participants for focus groups and individual interviews were submitted to the research assistant and the researcher. A total of 82 women of reproductive age group were included and participated in the focus group discussions which comprised of 7–12 participants in each FGD. For the individual in-depth interview, 18 reproductive age group women from nine kebeles involved. A semi-structured interview guide was developed for the interview and the participants were encouraged to speak up about their experiences. This deepened discussions and reflection on the life experiences of the women [28, 29].

Through focus group discussion the experiences and perceived benefits of contraceptive use of women of reproductive age were explored in detail [30]. Totally nine FGDs were conducted in three selected study districts and nine *kebeles* (health posts). Eighty-two women of reproductive age who were recruited on the basis of criteria engaged in nine focus group discussions where the number of participants in each session ranged from 7 to 12.

Discussions were arranged in consideration of the time and regularity and viability of rural women. All the discussions were conducted outside of market days and from 10:00–11:30 AM. Women were contacted through the women community leaders and the health extension workers regarding the date, time and place of the discussion. Focus group discussions were conducted in the health post closer to residential areas of the study participants making sure that all participants received equal attention to explore their lived experiences.

Following the focus group discussion, the individual in-depth interviews were carried out with women who had been using contraceptives for a long time (the minimum time considered for this category was 18 months consecutively). A total of 18 individual in-depth interviews were conducted by the research team in the residence of the women and also at the health post based on preferences of the interviewee. For those who were interviewed at their home, the research team was guided by the health extension worker or the community leader or both at the same time. A consent form was read and permission obtained (those who can write signed using pen and those who could not confirmed using their fingerprint) to continue the interview and record the interview in audio-tape and on paper.

Each interviewee was encouraged to talk about her life experience in detail without any apprehension or reservations. The interview continued in such a way for 40 to 60 minutes until the study team agreed that the ideas emerging became repetitious [27].

#### Data analysis

This study used the guiding principles of interpretive phenomenological methodology to explore the lived experiences of women’s’ contraceptive use and their perceptions of related benefits. Interpretive phenomenological analysis enabled viewing the practice or phenomenon in such a way that considers the close interaction between the participants and researchers as instances of their “being in the world” rather than only “being’ itself [31, 32]. In a sense, the final presentation of the data thus becomes an inter-subjective representation of the topic of the study. An adapted flow diagram from the interpretive phenomenological analysis (IPA) was used to guide the analysis (S1 File).

The process of data transcription and analysis was more complex as three languages were used in the research. The following steps describe the process: transcriptions were made of all the audio-taped materials verbatim, by Sidamigna and then translated first into Amharic and then to English. Materials were also translated back to Amharic by a linguistic professional. The Amharic translation was then given to the research assistant to translate back into Sidamigna, after which the document was reviewed for consistency. The three-step translation was mandatory as the principal investigator (the main researcher is not literate for Sidamigna). Therefore, Sidamigna to Amharic translation was necessary to fully capture the essence of the discussion. Study participants were given a chance to see the transcribed data and summary results and made comments based on their impressions by the help of the research assistants. Field notes were organized under the guiding research questions. Data immersion by the researcher took place by reading the transcripts several times. Repeatedly reading and re-reading the material revealed recurring ideas and concepts. In the data immersion process, several visits were made to the study participants as a first step in identifying descriptive codes and checking preliminary interpretations. The participants commented on some points following an initial description of the issue(s), and these were incorporated into the second round of data analysis with remarks. Margin notes and descriptive coding were then completed for all the materials. Data reduction was done in a step-by-step approach, beginning with the transcripts, followed by descriptive coding, and then distilling the material into themes by bringing similar ideas and concepts together.

Deriving themes was completed with consideration of both emergent themes and the research questions. The analysis made use of the idea of a hermeneutic circle; mainly, the back and forth iterative linking of data from both the perspective of both the researcher and study participants [33]. Summarized reports were presented to the study participants about the conclusions derived from their shared experiences. Discussions were held with participants about the study guide questions and core concepts of the study. Participant feedback was then considered alongside the experiences of the researcher.

Quality assurance or trustworthiness of the study used four criteria: credibility (truth value), transferability (applicability), dependability (consistency), and conformability (neutrality) suggested in the literature [34–36]. Trustworthiness in this study was ensured through: 1) presenting the summary of transcripts to the study participants to give them an opportunity for further comment; 2) reviewing of the preliminary findings to ensure that the early findings reflect what they know of the women’s lived experiences. 3) sharing the preliminary summary finding with the health managers and service providers to check interpretations; 4) indicating the detailed steps of the field work including the process of data gathering using the overlapping methods of focus groups and individual interviews.

### Ethical considerations

The researchers obtained ethical clearance from the University of Saskatchewan Research Ethical Review Board, Canada and Hawassa University Institutional Review Board, Ethiopia[S4 File]. Signatures were obtained from the study participants as facilitated by the research assistants in their local language and participants were assured the right to participate or withdraw from the study. Through follow-up sessions, the researcher and research assistant assured that the information gathered during the study was kept confidential. The back and forth translation of transcripts were presented to the study participants after initial analysis summary in order to assure the correctness of the information they offered. Anonymity of the participant’s transcripts and verbal record were maintained by using pseudonyms.

## Results

### Women’s perception of contraceptive use: Benefits toward empowerment

The study presents how the benefits of contraceptive use were perceived by women in terms of economic, educational and psychological aspects of empowerment. In the same way that women often discussed their experiences with contraceptive use, it discusses changes in the lives and livelihoods of the women and their families in relation to contraceptive service use, both before and after service use.

#### Economic empowerment

Study participants collectively expressed that their livelihoods in general were poor before contraceptive service use. It is indicated that when the number of children increases due to uncontrolled fertility, the family plunges into abject poverty. Related to this is the concern of land size in rural communities. All livelihoods are based on agricultural activities where arable land is the main means of subsistence. However, household land size is alarmingly reduced, consequently, the quality and quantity of productivity is diminishing. An experience of Adanech; a 25 year old woman having two children and using contraceptives for three years was explained:

“*I have small plot of land which is also not fertile. What I have done since I started using contraceptive service is that I herd cattle, sell some and earn money out of them. You see, herding cattle is labor intensive. You have to prepare fodder for them. To do so, you have to have enough time. Contraceptive use has averted unwanted pregnancy for me and I am free to use this time for collecting fodder for my cattle*.”

Women expressed that after contraceptive service use, their livelihoods have improved in several ways. It is substantiated by the words of Mutarie, a 25 five years old woman with no formal education and a mother of five children:

“*When I gave birth to many children in close gaps, I was unable to go to market. After I have started using this method, I am not waiting on my husband’s hand only. I grow vegetables in my garden such as cabbage and others. I sell some part of these and earn some money and use to eat part of these*.”

When women are able to postpone unwanted pregnancies and childbirths, they have more time to plan and engage in non-reproductive issues such as income generation.

*“Now I am a merchant working partly in the market*. *I have no worries like previously as there is no young child who needs my frequent visit*. *I send older children to school and then work whatever I can do*. *Contraceptive use has enabled most of us to engage in diverse income generating activities*. *Some of us became owners of better homes*, *others bought cattle*,*”* as evidenced by the words of Workie, a 30 year old woman with non-formal education who used contraceptive method for seven years.

Other women who participated in the study reported that since having more personal time as a result of using contraceptives, they engaged in credit unions, thereby becoming involved in an investment enterprise. These women have appreciated their experience of pregnancy planning for having created wonderful opportunities to improve their income and position in the community. Some women joyfully added that they were newly considered the pride of their husbands. Daetie, a 34 years old woman having 4 children and completed grade eight education, mentioned her experience as:

“*Now I do my work. I have poultry and garden cultivation where I work most of the time. The freedom has created wonderful opportunities for me to get involved in income generating activities and use the income to manage my home properly. I am now considered as a blessing for my husband, who previously abandoned me and my children when we were in living a miserable life*.”

Women using contraceptives also reported better use of the limited resources available to them and their families enjoyed the resulting psychosocial benefits.

*“We are now capable of taking level actions either to generate or expend income for minor household activities*. *Therefore*, *we are freed from looking for handouts and have started to exercise our rights and autonomy*. *Simultaneously*, *we learnt how to use our resources in an economic way*. *We relate all these to contraceptive use as it averted unwanted pregnancies and created opportunities to properly use our time*. *We no longer have such worries about cries of our young children*. *Our minds and hearts are cool and restful now”;* said Mogise, a 25 year old woman with three children and a grade six education.

Further, study participants articulated that contraceptive use has helped them improve their work culture, both in their family and the community. The capacity to work in various sectors improves, which includes supporting husbands in the field and directly working for oneself. This increases productivity in the field and raises household income. As stated by Janame, a 30 year old woman with a grade seven education, who used contraceptives for seven years, and has four children:

“*Contraceptive use helped me to avoid unwanted pregnancy, and created time and energy for engaging in various outdoor activities, and thereby generates income for my household use. Moreover, contraceptive use has enabled me to help my husband in various outdoor work activities. We have now more cattle, chickens, and better crop yields. My husband works in a more pleasant way than before. We have constructed a better home as compared to the previous time. When taking all this into account, our livelihood has tremendously improved*.”

#### Educational empowerment

In terms of education, women elaborated on the change in educational status in their family since they started using contraceptives. Being free from pregnancy means being able to move freely wherever they want; be it to school, market, social gatherings, health institutions, etc. Women also mentioned that they are cognizant about the benefits of education to their children’s future:

*“I see education for my children as a sole means to escape livelihood challenges and springboard for future prospects*. *With this intention I am greatly determined to send all my children to school*. *At this time three of my children are in school… I give more emphasis to my daughters*. *I have learned enough lessons from my sufferings*. *‘What do you talk about*?*’ Had I been well educated*, *I would never led a life like this*. *As a result*, *I am highly motivated to educate my daughters with a particular emphasis*. *I feel I am lagging behind in many directions*, *economically*, *intellectually and socially*, *for that reason I haven’t succeeded in my educational career*. *This is why I showed great commitment to my daughters and curious to see if and when they will attain high-level career”;* as shared by Dalibe a 30 year old woman with grade six education who used contraceptives for two years.

Remarkably, study participants also connected contraceptive use to enabling those who had dropped out of school to resume their education. Some of the study participants dropped out of school sometime in the past due to forced marriage. They were waiting for the right conditions to continue their schooling.

The following excerpt richly expresses this notion:

*“I gave birth to the second child after my older child reached grade nine*. *After that*, *I continued my education and received my diploma before giving birth to my third child*. *What helped me was a contraceptive method*. *I could have given birth to six or seven children if there hadn’t been a contraceptive service*. *There are women with such happenings*. *If I hadn’t used a contraceptive method*, *my education would have remained at grade eight where I stopped during my marriage*. *I thank both my Lord and the government who have given us the opportunity*. *I also became able to be employed at public sector”;* according to Mosone, a 27 year old woman with diploma education and two children, who used contraceptives for six years.

The study also revealed the experiences of some women where they had wonderful occasions to communicate with their husbands and plan on their education. Since contraceptives were being used, they were able to plan household education by setting a schedule for the education of both husband and wife. Somane, a 23 year old who completed grade six in school and used contraceptives for three years, shared her life experience by stating:

“*I have also a great desire and plan to continue schooling myself. I’m waiting until my husband completes his schooling, then I will continue. At this time, my husband is attending his school. Therefore, I am unable to attend because if we both leave the house, no one will take care of our daughter and domestic responsibilities*.”

#### Psychological dimension

Typically, in Ethiopia when a woman cannot regulate her fertility, she has several children in very close succession. This creates an increased burden on the woman’s physical, psychological, and social dimensions. Such a woman cannot properly care for herself, her children, and husband.

*“Our lives were about pregnancies and child birth*. *One comes just after the other in nine-to-ten months’ time*. *That was the time which we hated ourselves and our children*. *They were emaciated*, *not thriving well and not attractive to see*. *We ourselves were not well-fed*, *unhygienic and undernourished”;* mentioned by Aster; a 25 year old woman who used contraceptives for four years and completed a grade seven education.

Women contrasted such experiences with contraceptive service use, noting how it has created wonderful opportunities for them to either postpone or delay unwanted and unplanned pregnancies. They considered themselves as fortunate people by comparing their improved status to that of their mothers at a similar age. This was substantiated by the experience of Tadelech, a 27 year old woman who completed a grade seven education, and used contraceptives for 12 years:

“*I feel very happy. I feel so because, I am free from the burden of pregnancy, no fear about the unlikely outcomes of it and have time to share for other activities than only the child care. My husband is too. I take the service in agreement with him. I told him about the benefits of the service and he whole heartily supported the idea. In general, I have pleasant feeling about contraceptive services as it has many benefits for a woman like me, for children and community in one way or another. I have accepted contraceptive service for its positive outcomes*.”

In addition, women explained that their harsh feelings of the old days had been converted into the “bright shining days”. They explained how they had now reached a point where they could take the time to relax and take some rest in their lives. Women using contraceptives speak of reaching the actualization of considering themselves as fully human–as a person that can stand before anyone without fear. This is demonstrated by the experience of Munushe, a 28 year old woman with four children who used contraceptives for three years:

“I say ‘effoy’,*(meaning taking a deep breath and thanking the lord for the comfort I have now*.*) I say now ‘effoy’ comparing to my previous non-contraceptive use time*, *where I was forced to bear children in close manner*. *During that time*, *I had little time to care for them and hardly had time to rest*. *Now through contraceptive use I got relief from that*, *and for the last four years I have been living in ‘efoyta’*, *with peace and rest*. *Our lives before contraceptive use were full of burdens associated with young children and looking for ways to support them*. *It made our lives stressful (both I and my husband)*, *hectic and no ‘effoyta’*.*”*

## Discussion

### Contraceptive use and women’s empowerment

The present study shows contraception is fundamentally empowering women in four ways: 1) economic empowerment and agency 2) effects on personal autonomy 3) effects on mobility 4) effects on relationship. In this paper we consider economic, educational and psychological aspects.

In general, women’s experiences in this study revealed that contraceptive use is emancipatory and transformative to their lives. When it is said to be emancipatory, contraceptive service played an important role by enabling women to avoid unwanted or mistimed pregnancies and kept them free to be involved in many activities beyond the reproductive horizon. Studies in Bangladesh, the US and West Africa also support the conclusion that contraceptive use has improved women’s ability to be involved in productive work and other socio-cultural aspects by averting unwanted pregnancies[37–39].

Women’s experiences clearly indicated huge livelihood challenges in the study area. They expressed worries that their land size was diminishing, and its productivity decreased, as they were unable to allow the land to rest. Unplanned pregnancies and childbirths further stress household finances. Shiferaw [40], argues along this line, stating that when there is uncontrolled fertility, the population growth outpaces the capacity of natural resources, resulting in a poverty trap that further aggravates rural livelihoods.

Contraceptive service appears to be one of the mitigating factors in harmonizing the ever-growing demands on the limited resources of the household. Contraceptive use functions to harmonize the family income with family size [4, 10, 31]. Preventing unwanted pregnancies means creating increased opportunities for the household to generate more income. On top of their domestic responsibilities, women are able to engage in economic activities where they can generate income and thus boost their families’ resources. The income they generated helped them invest in the construction of better homes that improved the quality of their lives. Newly obtained free time has enabled women to think carefully about which income generating activity would best match their situation and provide the best return. They are able to think and plan; setting proper priorities to become involved in income generating activities and subsequently, the economic activities enhance decision-making capacity.

Women’s experiences further revealed that when their chances of being involved in income generation increases, their capacity to properly manage resources also improves [10]. Following the implementation of contraception services, women in the study area became more conscious of the use of resources at the household level taking charge to minimize wastage and control of their older children’s expenses. Further, they learned that they have obtained opportunities to minimize waste and preserve resources for unexpected needs. This is another golden experience that could further improve community and national development through saving and investment.

Education is one of the most influential means for women’s agency. It helps to mitigate household and individual-level poverty and livelihood challenges [7]. However, women in the rural part of Ethiopia have not been able to fully enjoy the benefits of education. People in the study area discouraged girl’s education by saying that “the best education for a girl is to master domestic work through helping her mother at home.” Another excruciating condition was early marriage, which often ruined girls’ educational chances. A particularly humiliating local saying in the study area, which works against girls’ education, was “let the girl and a dead body leaves the house early”. This was in line with culturally embedded implications that if one invests in a girl it is only benefits others and not the family, as a girl ends up married anyway, which requires resources to be transferred from her parent’s house to her husband’s house.

Girl’s education is also hampered by household work. A mother, heavily burdened with domestic work, often does not wish to send her daughter to school; rather, she prefers her daughter stay home to help her with household duties, feeling that, “what good is an education while I am suffering with continuous domestic work burdens”. This dynamic not only denies the privilege or right of a girl to attend school but also disgraces her human dignity. According to Kruger *et al*.,[41], it is argued that women’s oppression results from social arrangements inherent in a society, which can be changed. In connection to that, family dynamics result in women’s subordination since these are based on dominant and subordinate roles legitimized by society.

Some of the women in the study were observed to exhibit changes in their thinking in this regard, stating they wish better futures for their daughters. At least some of the participants discussed how contraceptive use enabled them to send their daughters to school, or to continue their schooling after dropping out due to forced marriages. Contraceptive use has therefore shown that it can emancipate and empower women by allowing some of them to complete their secondary schooling, join tertiary school, graduate, and become employed in better paying jobs, but that it is not a straightforward path.

The connection between economic and educational empowerment is also related to contraceptive use, in that obviating an unplanned pregnancy was helping women generate greater revenue, which could go towards their children’s schooling. This finding is further supported by a USAID report that showed that couples with the means to control their fertility were often able to invest more resources in each child, which ultimately raises the standard of health, education, and wealth in the population [42, 43].

A woman’s psychosocial health is affected by several factors. Unregulated fertility is one of the major factors that limit women’s capacity. Women’s experiences have revealed that contraceptive use has helped in myriad ways. It helped the women plan their pregnancy and childbirth and manage their time for personal care and development. Unlike life prior to contraceptive use, they are able to better maintain their cleanliness, improve their self-image, and esteem. They feel proud and fortunate to live in a time with available contraceptive services by which their visibility and status in society can be improved. This finding therefore supports other studies conducted on the influence of women’s perceived body image on their marital and sexual relations. When women perceive that their body image is poor or weak, they can feel poorly motivated to engage various socio-economic and personal affairs [44–45].

When a woman generates her own income, she obtains relative financial autonomy instead of waiting for money from her husband. In rural Ethiopia generally, and in the study area particularly, finances in the care of the husband are often out of reach for expenses related to child care. Conversely, money in the control of a woman can easily be accessed for child care and other family members [46]. Moreover, when a woman attains otherwise unobtainable finances through contraceptive use and used the money to support her family, she feels proud and confident. Barroso^12^, similarly notes the experiences of women who have control of their sexuality and fertility through the use of contraceptives have created opportunities for them to be involved in other aspects of life outside the mere reproductive dimensions.

Sonifield et al., [47],argue that “education, employment, income and relationship stability are connected to mental health, happiness and quality of life for individuals and couples. By affecting these central life experiences, access to contraception may also affect mental health and well-being”. The present study reveals that a woman’s experience of psychological empowerment was directly related to personal autonomy following contraceptive use. Some participants equated this to a time when darkness changed into light and nights changed into days. Additionally, the fears related to unregulated and unplanned pregnancies have changed into happy and joyful planned outcomes. Contraceptive use has enabled women to postpone pregnancies to a time when they are ready. Indeed, avoiding an unplanned pregnancy created more time and resources for women to care for their family members and themselves.

Women using contraceptives in the study felt confident and stood firm before society, instead of feeling shy and looked down upon, as they received respect and love from their husbands and other members of society. Women’s attitudes and feelings have changed from the hectic and fearful to satisfied, peaceful and stable. Studies agree with the finding that the ability to delay early childbirth has many advantages such as improved earning potential, ability to send their children to better schools, enabling them to follow up on their children’s school attendance and improved marital relations by reducing domestic burdens [48–49].

Within the study, women’s visibility in society improved because they had enough time to become involved in social activities after completing their domestic work. Women’s capacity to accomplish their duties in the manner that they desire also greatly improved. Cleland, et al., [5], ascertained that contraceptive use, apart from the socio-economic considerations, allows the attainment of fundamental human rights to choose the number and timing of children. They explain further that contraceptive use created “freedom from the tyranny of excessive fertility” which has been called the fifth freedom, alongside freedom of speech and worship and freedom from want and fear.

Women’s experiences also revealed that they were involved in various community affairs following contraceptive use. They organized themselves into various enterprises where they received credit and were able to mobilize that credit to generate income, save, and spend for their family needs. They were also involved in other community affairs such as leadership positions for development organizations (named as development army), community networks, and model women’s groups in the community. Following contraceptive use, women were able to attend social gatherings, religious ceremonies and access health services. Women’s capacity to discuss family matters with their husbands improved as well. They discussed matters such as household expenses, children’s education, health services use and income generating activities. Women’s experiences in this study correspond with other study findings that when women are able to delay their pregnancy, it helps them reach to the destiny they want and paves the way to becoming involved in socio-economic arenas [50,51].

Decision-making processes and actions are among the core aspects expressing women’s empowerment. It usually relates to the level of power one has in the social system. Women in the developing world in general and in rural areas in particular experience challenges related to a very low social status, which hampers their decision-making capabilities both within the household and at the community level. Their decision-making experiences as they lived through contraceptive use have shown grey areas unlike other dimensions of their empowerment. Women’s experiences in their decision-making revealed that they have relative freedom only for domestic decisions such as cooking food, fetching water, cleaning houses, and washing clothes.

Women’s capacities to make decisions on major family affairs are contingent on their husbands. With the majority of decisions, the husbands are the ones make the final decision. In order to leave their houses, women often need to notify their husbands. The present study revealed that women have the liberty to make decisions only regarding lower-level domestic work; their power to decide on higher-level issues is diminished. Though there have been some improvement in the process of decision-making through household-level discussions, rural Ethiopian women often have less freedom to make their own decisions, thereby indicating the continued imbalance of power between husband and wife. As such, decision-making ability is one of the least privileged and slow-moving aspects of progress in women’s autonomy observed in the study area. Together with contraceptive use, women could benefit from empowering universal affirmative actions. The extraordinary experiences of contraceptive use related benefits should be extended to all other sectors of development.

## Limitation and delimitation

One limitation of the study was that it considered only married women, and included participants from only three districts of Ethioipia. The sensitivity of issues surrounding contraceptive utilization may be one factor that limited responses during data collection. Another possible limitation is that the study hasn’t considered current non-users’ perception about the benefits of contraception. Men are also not considered in this study as the prime purpose of the study is to explore the experiences of women.

The delimitation of this study is explicated in terms of the study purpose, the selection of the study area and selection of study participants. The study was carried out with the intention to explore the experiences of female contraceptive users in the Hawassa University research villages, which were established with the intention to observe the impact of university-based research in knowledge generation, technology transfer and the livelihood of the residents.

### Conclusion

This study was conducted with the broad aim of improving the overall understanding of policy makers, health service providers, service users, researchers, and activists about women’s perception of contraceptive use towards empowerment and how these experiences would help in improving access and utilization of contraceptives by the current non-user women. Based on the study findings it can be concluded that in the life of Ethiopian women, the overall use of contraception has created a remarkable means to control their bodies, their reproduction and their fertility. Contraceptive use has freed women from worries and traps related to unplanned and unwanted pregnancies and childbirth. It has opened wide opportunities for women and it has offered “peace and stability” in their lives. The study recommends introducing a clear strategy that allows the encouraging experiences of current service-user women to be shared with their non-user counterparts in order to ensure the expansion and sustainability of contraceptive service.

## Supporting information

S1 File Data analysis flow diagram adapted from IPA (Smith, et al, 2009, pp. 82–100.). (DOCX)

S2 File Transcription of individual in-depth interview. (DOCX)

S3 File Transcription of FGD. (DOCX)

S4 File IRB document. (DOCX)
