# Supplementary material for: Women's perception about contraceptive use benefits towards empowerment: A phenomenological study in Southern Ethiopia
Source: PLoS One. 2018 Sep 13;13(9):e0203432. doi: 10.1371/journal.pone.0203432 (PMC6136733; doi:10.1371/journal.pone.0203432)
Supplement: S1 File — (DOCX) [file pone.0203432.s001.docx]

**Annex # 2. Data analysis flow diagram adapted from IPA (**Smith, et al, 2009, pp. 82- 100.**)**

The qualitative data analysis steps adapted from IPA and customized to fit my study taking the following steps as indicated below in flow diagram.

Step 5. Connecting themes and summarizing themes into study questions

Step 4. Developing emergent themes

Step 3. Descriptive coding

Step 1. Reading and re-reading

Step 2. Initial noting/side noting
