# Supplementary material for: Women's perception about contraceptive use benefits towards empowerment: A phenomenological study in Southern Ethiopia
Source: PLoS One. 2018 Sep 13;13(9):e0203432. doi: 10.1371/journal.pone.0203432 (PMC6136733; doi:10.1371/journal.pone.0203432)
Supplement: S2 File — (DOCX) [file pone.0203432.s002.docx]

**Transcription of individual in-depth interview**

Individual in-depth interviewee # 1 Profile:

Kebele: Ganne

Age: 24

# of children: 4

Education: 5

Duration of contraceptive use: 5

Marital status: married

Type of contraceptive method used/using: depo-provra

Time interview started: 1: 10 Pm; ended: 1:45

What is it like to live through contraceptive use towards your status with the respect of your income status?

- - - What is your experience about economic status as to fulfil your basic need, your children and the family as a whole?
    - What is your lived experience like as far as you started to use contraceptives?
    - How you experience the situation in improving your income level at your individual and family level?
      - How you express this in relation to your former non-use time and the current non-user counter-part?

Answer/response

I live in our house together with my husband and our four children. Our livelihood since I have started using contraceptive service is well. We live peacefully and no problems encountered as of the time I have started using the service. Our dominant means of livelihood is agriculture but we also involve in other sectors such as small scale trading. We do involve in trading mostly during winter time where our agricultural involvement is minimized. Contraceptive use has created remarkable opportunities for me to involve in different income generating activities. Let me put this in this way. Before the time of contraceptive use most of my time is occupied in pregnancy and child care. I have many domestic responsibilities such as preparing food, fetching water, collecting fire woods, caring for children and also support my husband in agricultural fieldwork. These responsibilities usually doubled when I become pregnant and bear child. Contraceptive method use has eased my burden enabling me to space pregnancy and child births. In general, my livelihood after contraceptive use has improved. I am able to support my husband in different outside home activities and I also generate my own income going to market. As a result, I am able to spend some money for myself and my children necessities.

*What is your lived experience related to your educational status or your family members including your children?*

- - - *What is your experience related to your schooling situation, your children, particularly your daughters if any?*
    - *What is the experience with female education related to the contraceptive use? How do you explain the importance of contraceptive use in relation to sending your children to school?*
    - *If you haven’t reached your educational destiny, what do you explain for us about it as related to the availability of contraceptive methods by then?*
    - Do you think that it could have been different than what you experience now? In what manner? Can you tell us in detail about that?

I was attending school before my marriage. I learned up to grade five when I forced to stop my schooling due to marriage by abduction. Since then, I didn’t get opportunity to continue. However I send my children to school. I send both boys and girls to school as their age due for schooling. Three of my children are in school now; the first one is grade 8, the second one is grade seven and the third one is grade four. The four one is too young to be in school and waiting for appropriate age. I treat both daughters and sons equally in relation to sending them to school. This is evidenced by that my first child is female and she is grade eight followed by her brother who is seven. Contraceptive service use has helped me to send my children to school by preventing unwanted pregnancy to happen. If I get pregnant, I will be overwhelmed with many burdens both from pregnancy, child birth and other domestic responsibilities. Now, contraceptive service use has created nice chance for me to space pregnancy there by use my time to better care for my children and send them to school.

*What is your experiences related to physical movement outside from your house? Such as going to market, health institutions and other social gatherings?*

- - - *How you explain that in terms of frequencies, conveniences and challenges?*
    - *What is your experience particularly your movement in terms of easiness, getting adequate time to do so and any other experiences you would like to explain?*
    - *Probe here who is the primary responsible person for the decision to move outside the house.*

With regard to attending meeting, I have participated one meeting at kebele level which was about saving and credit. Since then I haven’t attended any meeting. I have participated in the health education program at the health post during I visit for contraceptive service use. Otherwise, I haven’t attended any special types of meeting in my locality or outside. I have no as such difficulties to attend meetings to attend at this time. When to put this in the era of contraceptive use and before, now I have better time to accomplish my domestic works and spare some times for significant social affairs. Before, contraceptive it was known that my times were occupied either by being pregnant or caring for young children. These were some of the reasons earlier prohibited my participation in many social and economic affairs outside my home. You see when you are overburdened with routines including child care household activities and outside home activities, you cannot think to move out from your home. Moving outside of your home in such situation not only harm your personal integrity but also end up in upsetting one’s husband and results in conflict. However, as of the time when I have started using contraceptive methods, we have mutual understanding with my husband whether to go somewhere outside my home or stay home.

*What is your experience related to your decision-making position at your house hold level in terms of: resources mobilization, movement and other domestic decisions?*

- - - *What experience could you share us about how decision made in terms of domestic expenses, health services coverage, schooling payment and procurement of clothes and school items for your children?*
    - *How do you practice your decision making at household level? Who is the primary decision maker? What are the patterns of decision-making at household level?*
    - *What is your experience in this regard in relation to your contraceptive use?*
      - - *Probe at this point as far as the information get saturated*

*What unique experience exhibits the use of contraceptives? How the experiences of user relate or differ to that of non-users?*

For any decision in our house we decide together however, my husband has upper hand in passing decision remarks and following him I do add my view. I go to market, health institutions occasionally. I have no problem of getting permission to go for such issues from my husband. *What is your experience in deciding on issues without your husband’s knowledge?* As I said earlier my husband is the first to pass decision remarks. There is nothing I do without his knowledge. I have to consult him before reaching. My husband is the one who put the final decision on my children educations. Surprisingly, all my children do fear and respect their father than me. Therefore, I leave all the decisions to him to execute. *When you have started to use contraceptive services?* I first started using contraceptive service in 2001 E.C. The reason for my contraceptive use is primarily to avert unwanted or mistimed pregnancies and births and to properly handle my children. I heard about the contraceptive services from the health extension workers for the first time and decided to use injectable method which so far I am on. I chose this method as it is convenient for me and suits my desire. *Do you know any other method than what you are currently using?* Yes I know oral contraceptive pill, implanon buried under the upper arm, and loop. But I am not comfortable with other methods than I am using.

You have told as now you have four children. *What do you think would happen if you weren’t using contraceptive service?*  I am quite sure that I would be in great problems. I would bear children in close gap and my children would be emaciated for the fact that I could not give them adequate and balanced diets. They would hardly get enough breast milk as well. Contraceptive use has eased my children’s life and growth. I am able to postpone pregnancies and now free from it that I feel healthy and comfortable. I have no problems now and do whatever thing I wish to do so. Generally I feel comfortable except that my weight seems decreased. However, the benefits out weights that of the risks in overall conditions.

*What is your experience related to your health status?*

- - - *In terms of your overall feeling good?*
    - *Your nutritional status, weight, types of diet consumed*
    - *Visiting health institutions*
    - *Reasons for the visit*
    - *Any illness that made inactive, bed ridden, etc…*
    - *What is your experience in your health status in relation to your contraceptive use? How do you explain this in terms of improvement or deterioration?*
    - *What is your experience in relation to contraceptive utilization and fertility issue*
      - *How this can be explained in comparing the patterns before and after contraceptive use? Probe more ……*

With regard to the health situation in the connection to contraceptive use, when I compare to the previous time, we all are doing well. Our health status has improved as a result of contraceptive use. Before contraceptive use, I got pregnant frequently that weakens my health status. Being free from pregnancy itself gives you comfort and sense of freedom. Moreover this enabled me to participate in activities which I couldn’t do earlier. I get involved in trading and income generating activities there earn some revenues. My husband is also happy about the service use. Contraceptive service use has created better chance even for my husband as I support him in some outside field activities. When I delay pregnancy, both my children and I feel well and healthy. I feel that I am lucky and most fortunate that I got this chance. I am also thankful to the government that has established this service in our vicinity.

*What do you recommend for those currently not using the services?* I know there are some husbands still say that children are wealth and prohibit their wives from using the services. They do this from ignorance; therefore, I will first approach a woman and tell her about the different types of contraceptive methods and how they help us. I will encourage her to come to the health post and listen what the health worker could advise. In my part also I will inform about the benefits of contraceptive service in enabling to properly manage her children before looking for more. I also tell her my experience and the benefits I obtained from contraceptive use.

Individual in-depth interview -2

Kebele: Waycho

Age: 30

Education: 7

# of children: 4

Year of contraceptive use: 7

Method used: Depo-provera

*What is it like to live through contraceptive use towards your status with the respect of your income status*?

My life experience after contraceptive use and before it has remarkable differences. Before contraceptive use I gave for two very close births where I had devoted all my times for them. I had not time to move out of my home in order to participate in other income generating activities. Our major source of income is agriculture where my husband is predominantly engaged in it and owes it. With contraceptive utilization, I am able to delay pregnancy and child births and therefore, participate in some small scale trading where I generate revenue at my level. I mean that contraceptive method gave me opportunity to get adequate time and spare this time to go to market and involve in trading. As I have stated earlier, it was impossible to do so before the contraceptive use. I had used contraceptive method for eight years before my first birth and then have given for my first birth. It is also seven years after I started using contraceptive method after my second child birth. -

*What is your lived experience related to your educational status or your family members including your children? More specifically, tell us about your experience on female education?*

I see education for my children as a sole means to escape livelihood challenges and springboard for future prospectus. With this intention I am greatly determined in sending all my children to school. At this time three of my children are in school. Two of them are grade eight and the third one is grade seven. The last one is too young to school and not yet started schooling. *What is your experience in relation to female education? Do you see both your sons and daughters equally with respect to education?* I give more emphasis to my daughters. I have got enough lessons from my sufferings. “What do you talk about?” Had I been well educated I would never lead a life as I live now. As a result, I am highly motivated to educate my daughters with particular emphasis. I feel I lagged back in many directions (economically, intellectually and socially) for the reason that I haven’t succeeded in my educational career. This is why I show great commitment to my female children/daughters and curious to see when they attain high level career.

*What is your experiences related to physical movement outside from your house? Such as going to market, health institutions and other social gatherings?*

I have no as such problems in going out of my home. I go to market to buy stuff needed for my family as well as purchase items for trading. I sell these items in local small market in order to get some profit out of them. I go to kebele to attend meeting as invitation come from respective organization or association. Mostly, I attended meetings in the kebele focused on awareness creation on harmful traditional practices such as early marriage, tuberculosis control, disadvantages of contraceptive non-use, etc….The meetings were organized by the district health office, the health post and kebele administration. *What is your experience in establishing discussion sessions with the health extension workers in issues related to your health?* I have very good experience in exploiting the health extension workers in relation to my health situation. By the time when I started using, depo-provera, I suffered irregularities in my menstrual flow. Then I told the problems to the health extension workers and they advised me to wait some times until the body adjust to it. Likewise, after some times the problem has subsided and now I am okay. I also visit health post to get vaccination service both myself and my children. At this time all my children have completed vaccination and I only visit to the health post every three months to get depo-injection.

*What is your experience in relation to your general health status? What do you fell now? Do you feel healthy or in contrary?*

With respect to overall health status of mine and that of my family is well. At this time we have no obvious health problem in our family. I feel healthy at this time. *What is your lived experience in relation to getting balanced and adequate food?* More or less my family members are having enough food and feed on regular base. I have no health problem identified by health professional. My children are well and grow properly. They caught their proper height and weight as compared to their neighbour counter parts. More specifically my children born after contraceptive use are growing better way than even their older brothers and sisters. When I relate these all changes or current status, it is all about contraceptive benefits. You see…., I got better time and relief from burdens related to unregulated fertility due to unplanned pregnancies and births by using contraceptive method. It has given me opportunity to care for my children in better way. *Where do you seek health service when your family members get sick?* What I do when any one in my family get sick is I take to the health post. If the condition is beyond the health post, they instruct me to take to health center and I do so.

*What is your experience in domestic decision making at household level? In respect to resource mobilization, sending children to school, going outside home for social, individual affairs.* Most of the time decision is made mutually by discussing with my husband. In condition where he is outside house, I do it alone. So long I haven’t come across difficulties in handling family matter related to this. I do by myself when I wish to send my children to school or to go out of home for various social and individual matters.

Individual in-depth interview #3

Kebele: Wyacho

Age: 30

Education: 6

# of children: 4

Year of contraceptive services use: 2 years

Interview started: 8:15 and ended 8 40

*What is your lived experience in relation to contraceptive use with respect to your overall livelihood status?*

I had started contraceptive use and was able to space pregnancies and child births long ago. However, I discontinued in middle due to health problems. I got sick while using the method. As I stopped contraceptive method, I gave two consecutive births in close gaps. But I was trapped in many life challenged and finally decided to use contraceptive again whatever health problems I may anticipate. I recognized very well that the problems of pregnancies and child births related to their subsequent burdens over weights that of the side effects of the contraceptive method. *Was the health problem you mentioned related to contraceptive use?* Sure. I used depo-provera for eight years when I developed a leg ache and weakness/flaccid at my legs. For fear of the problem, I stopped method use. Then, unlike the previous time I gave two births closely and again immersed in more puzzling condition. Finally, I have decided to continue method use as I realized that the problem of contraceptive method use is less risky than unwanted pregnancy and its related consequences. This had happened after my second child birth. As I have mentioned earlier, I used contraceptive method for eight years after my second child birth. It was that time that my legs started to ail me. When I shared the information, they told me that it could be due to the prolonged use of depo-provera and advised me to stop for some time. Hence, I got pregnant as I discontinued the method. I again turn to use it. I came to this decision as I weighted the problems of pregnancies and child births in comparison to method use. In my opinion the prime benefit of contraceptive use in my life is that it enabled me to space pregnancy.

*What is your experience related to improving your livelihood as you lived in contraceptive use?*

Let me first tell you about the process and consequences of pregnancy and child birth. Pregnancy ruins your physical strength. When you are pregnant, it is difficult for you to act in the manner that you are not pregnant. It limits your capacity to engage in various activities you may generate income. Contraceptive method use helped me to avoid unwanted pregnancy there by created time and energy for engaging in various outdoor activities thereby generates income for household use. Moreover contraceptive use has enabled me to help my husband in various outdoor works. We have now more cattle, chickens better crop productions. My husband works in pleasant way than before. We have constructed better house as compared to the previous time. When taking these all into account, our livelihood has tremendously improved.

*What is your lived experience related to your educational status or your family members including your children? More specifically, tell us about your experience on female education?*

I personally attended school up to grade six. I have dropped out schooling in 1987 E.C., since then I have never been back to school. My husband has attended schooling up to grade ten. Recently he is processing driving licence. *What about your children’s schooling?* My first daughter is grade ten. Her younger one is grade five. But when we saw his performance, it was not adequate to his level and we decided him to reduce two grades down and now attending grade three. He has significantly improved his performance and competing with top class mate students. The remaining two are too young for schooling and waiting their age to go to school. *What encouraged you to send your female child to school? Did you able to compare your life with your mother and grandmother’s? What is your experience in encouraging her to better perform in her schooling?*

I am encouraged to send my daughter to school from various points. I am the only one who is housewife among my sisters. They got educated and now employed in various organizations and lead better life. I recognize education will pave ways to my daughters for better future. With this intention, I sent my older daughter to Hawassa, where she could get better education as compared to Leku, where she has attended schooling in past. She attends now in one of the private school with better quality of education. I am sure that this school will prepare her in better way than the school where she was. When I try to look into the overall livelihood situations of today and sometimes in the past (during my mother’s or grandmother’s time), this time life situation is becoming complicated. In past, though there were no contraceptive services, the number of people to the land carrying capacity is less. As a result population ratio to household agricultural production is in faire balance. I know that my mother and grandmother gave as many children as they could but they lived better life with respect to obtaining better nutrition. They took care of their children in proper ways. *Was there any thing that made you to regret about the past time as if you would say if this or that?* My parents had better economic capacity than what I have now. Therefore, my parents cared for us in appropriate ways. I know that my mother have never used contraceptive service but she was lucky that her pregnancies and child births were naturally spaced.

*What is your experience related to your health status?*

I feel sick some times. I have head aches and joint pain. *What is your experience with regards to having adequate and balanced food at your house?* So long as I feel and understand, I can say that I have enough. I don’t exactly compare what is adequate and balanced but I eat what I have without starvation. Except that I feel sick sometimes, otherwise I am okay with feeding. *What about obtaining proper weight to your size?* I feel that my weight is less than what I have to bear at my size. The reason is that I feel sick sometimes. Another factor may be in past I had two close pregnancies and births. That also has contributed to the recent body status. You see….., when you have two young children at home, these will make you enough busy. You have no rest and time to eat well and sleep well. I was hospitalized during my third pregnancy. I faced bleeding at fourth month of pregnancy. They examined me using ultrasound and finally evacuated the conceptus material. They admitted me and managed the problem. *What do you tell about your children health status by comparing with children born by mothers not using contraceptive methods?* It is clear that children born in spaced gaps have better chance to be cared and nurtured. The better care they obtain the better health outcomes they exhibit. My children’s health status is well. They grow well. Their body weight, height and overall make up is in good status as compared to their ag fellow children in the neighbourhood. One of my children had minor health problem and went to health institution.

*What is the feeling of your husband about your contraceptive use? Does he support or oppose?* My husband is in agreement with me about contraceptive use. We discuss the matter each other. He reminds me about my contraceptive method appointment date if I forget it. He is happy about contraceptive use. We openly discuss about it. He asked me when I stopped last time why I did it.

*What do you say for those women not currently using contraceptive services from your life experience? What is your experience about the beneficial and drawbacks related to contraceptive services?*

Contraceptive service is a means to space pregnancies and child births. Therefore, these benefits on top of the others are among its merit for women. As I mentioned above, I have benefited by spacing pregnancy. Now I am not pregnant and no very young child at my house. As a result, I go to market and engaged in small trading activities. I also become able to move to various places for my personal and social issues outside my home, which I could not do when I was pregnant or have young child.

*What is your experience with regard to decision-making at household and individual level? Who decides at your household level?* Mostly at our family decisions are done jointly between I and my husband. However, the principal decision-maker is my husband. I have never attended meeting at kebele level. No one has invited me to do so. My major duty is handling domestic matters. Occasionally, I also go to market. I want to share my experience to those women who are not currently using contraceptive by disclosing the multidimensional benefits of contraceptive use. More specifically, for women it enables them to space pregnancies and give time to get rest and relax.

III # 4.

District: Boricha

Kebele: Konsore Fulassa

Age: 23

Educational status: grade 6

# of children: 1

Year of contraceptive service use:3

Time interview started: ended:

*What is your lived experience in relation to contraceptive use with respect to your overall livelihood status? In terms of with whom you live, what is the source of your income and how many children you have?*

I live with my husband in our private house with our daughter. The main source of our livelihood is agriculture subsidized by occasional small scale trading activities. When I say occasional trading, we participate in interrupted trading. When the agricultural involvement is intensive, we pause trading and fully engage in agriculture. By the time when agricultural activities become loose or less intense, we shift to trading. *What is your experience related to education in your family? What is your experience with female education including yourself?* I am cognizant about education in general and female education in particular. Thus, I am waiting for my daughter’s age appropriate for schooling. She is now too young to go to school. I have also great desire and plan to continue schooling myself. I wait until my husband completes his schooling, then I will continue. This time, my husband is attending his school. Therefore, I am unable to attend as if both we leave our houses, no one will take care of our daughter and domestic responsibilities.

*What is your experience related to attending various meeting at kebele, going out of your home for social and personal affairs?*

I mostly attend meeting at kebel as they invite me. I have so far attended meeting focused on environmental sanitation including proper construction, utilization of latrine; and saving. I haven’t attended educational session about family planning. I visit health post frequently to collect my contraceptive method, to vaccinate my children. I go to market as far as I have issue to do so. I tell my husband before I go to market but not necessarily ask him permission for every incident to move out. In case when I have no money at my hand, I ask him to give some money to go to market.

*What is your experience related to your health status as you live in contraceptive service use?*

I am okay with my health. There are no health problems diagnosed by health professionals. I do fine with having proper physical and psychological health. My body weight is proper to my age and height. I eat well and adequately. *What is your children health status?* I have no many children and currently use contraceptive service. Therefore, I have good time to care for my daughter. I gave her breast milk adequately. I also give her better and balanced food. As I have time to spare for her, I do share with her good time, cuddle her, play and smile with her. She has no any medically identified health problems. *Where do you go seeking health services if any family member gets sick?* First we visit to health post and if anything beyond the health post, we go to health center. In my experience so far I and my family members never went to traditional practitioners for health services. Neither have we stayed home being sick. We at outset visit to health institutions whenever we have such problems.

*What is your lived experience with respect to movement outside your home to various places such as market, social gatherings and patterns and likely to decide on personal and family affairs?*

I go to market ones in a week and nothing so far had happened. I plan to go to the market but inform my husband before I do so. I never do it without his knowledge. I take money from my husband to go to the market. I do nothing without his knowledge or informing him. Whatever thing I want to do, I have first inform him. These include: going to market, expending in the market and involving in small scale local trading. We jointly plan in which temporary trading should we involve. *You have mentioned that your husband is now a student and if you wait him for every small piece of domestic decision, how that could affect your accomplishment?* I do all domestic duties alone that include: food preparation, preparing coffee, washing clothes, etc….. I also wash my child and maintain all domestic cleanliness without having his go ahead.

*What is your lived experience in relation to your contraceptive use, livelihood and health status generally?*

It is about three years since I have started using contraceptive method use. I heard information about contraceptive services from my neighbours and went to have more information from the health extension worker. The health extension workers have given me with detailed information about contraceptive use and finally I have decided and started using. I have so far used depo-provera injection and now stopped it with intention to have another child. It is about five months since I have stopped using it. I know no other method than the one I have used. *What benefits you got by using contraceptive method?* I have got many benefits; first and for most, I got rest and comfort thereby have got better time to care for my child. I personally benefited by maintaining my good health status. I feel well and healthy. I am not undernourished and feed well. With extra time I have due to contraceptive use, I have engaged in cultivating my yards and grow vegetables and also involved in small trading activities where I generate some income for me. In addition to the above, I have properly used my time to keep and maintain my cleanliness. *What is your feeling about bearing a female first time?* I feel happy as my first child is a female. I know that children be male or female are gifts from God. My husband too, in this issue. *What are your overall comments about contraceptive use?* I strongly agree and share that contraceptive use have multiple benefits. Its benefits out way its side effects and therefore, I strongly urge those women who are not currently using the services to soon come to the service and enjoy its benefits as we do. I know some women are still resistant to listen this information. However, I stand firm and detrimental to share my experience how it helped me to space unwanted pregnancy and involve in many social, economic and personal affairs. It is quite obvious that un-spaced pregnancies and births harm both mothers and children in many ways. Therefore, I say to them please use!

III # 5.

District: Boricha

Kebele: Konsore Fulasa

Age: 30

Educational status: - (cannot read and write)

# of children: 5

Years of contraceptive use: 10 years

*What is your lived experience in relation to contraceptive use with respect to your overall livelihood status? In terms of with whom you live, what is the source of your income and how many children you have?*

I live in our house with my husband and our five children. In so far as I recognise, we have no as such major life challenges or big problems. Our major source of income or livelihood is agriculture supplemented by some trading activities. I sale local alcohol (areke) to generate additional income for my family. *What about educational status of your family members including your children?* My first child was a female one. She attending school up to grade six and marries by dropping out her school. She married at her 14 years age. The marriage was consensual and here in nearby. No one has forced her to do so but I guess it was her decision. *What is your intention to send your other children to school despite your older one had discontinued?* I have very strong feeling and desire to educate all my children. I was not in agreement even with my first child who has married by dropping out her school. Recently I have one female and one male child attending their school. Two of my sons are still outside school; I can say that they are cheated (ተታልለው), as they have engaged in local trading. I am committed to support all my children at school with great emphasis to my daughter.

*What is your experience with regard to attending various meetings at kebele and community levels and going to markets and other social gatherings?*

I occasionally attend meetings at kebele level as I get invitation. Among the meetings I attended, sessions on health education at health post addresses issues of family planning and environmental sanitations are few to be mentioned. I go to market very occasionally as I have a lot of domestic work burdens. I get money from my husband before going out to market. Sometimes, my husband buys whatever things needed to us from markets. With regard to decision-making at our household level, there is nothing I do decide without the knowledge of my husband. My position is only to support him. *Where do you get money if you want to buy something to your children?*  When I have money, I give it to my husband to purchase it or if I get out of money, my husband does it as he is conscious about it. Most of the times, we effect all decisions by discussing together.

*What are your experiences with respect to your health status and that of your family planning? How do you relate your health status with contraceptive use?*

I had suffered from hemorrhoid in the past but by the help of medical intervention I got rid of that problem. Now my health status is okay. I have no obvious health problems either I feel or told by the health professionals. I am in peace now. Concerning my children’s health condition, none of them have been sick so far. All of them are doing very well. *Where do you take if any family member gets sick?*  Our preferred health institution is Fulasa missionary clinic. We visit this health institution in case if any members of the family got sick. We don’t want to go to other place.

*What is your lived experience with regard to the time when you started contraceptive method, what has motivated to do so and who was the initiator for the services?*

It was about ten years since I have started using contraceptive method. The very purpose to start contraceptive use is my recognition to the ever changing livelihood situations particularly shortage of land. You see, unlike the past times now a days land holding capacity at house hold level is rapidly diminishing. I have five children (three males and two females). I have decided that these five children are adequate for me as the current situation is taken into account. First I heard information about contraceptive method from Yirba health center. I use injectable method. I started using method forty-five days post- delivery. I have come across no problem so far. What other methods than the injectable contraceptive method do you know? On top of the method I use, I heard about surgical contraceptive methods and contraceptive pills. *What is your experience about the benefits of contraceptive services as you have lived in it?* I say I got rest. *How do you explain rest?* It freed me from the now and then pregnancies and its related child births and cares. The free time I got has enabled me to involve in income generating activities. As a result, herd sheep, goats and poultry. These resources can fill some gaps in my family when need comes. If I did not use contraceptive method, I guess my life would have been deteriorated and messy. Contraceptive use has made me happy. My husband is also happy. Our family members involve in various income generating activities. My older children have started to generate their own income.

Generally, contraceptive use has created great opportunities in our household. More importantly, I am free from unwanted and mistimed pregnancies which have been bottle necks for the health and wealth of women. I thank both our God and the government for offering me such opportunity. If I am not using this method, I know I have many children beyond my capacity. It is clear that having many children would ruin my family capacity. On top of my personal contraceptive use, I share my experience with my neighbours who are not currently using contraceptive methods. I inform them the tremendous benefits of contraceptive method succinctly.

III #6.

District: Boricha

Kebele: Konsore Fullasa

Age: 25

Educational status: cannot read and write

# of children: 2

Year of contraceptive use: five years

*What is your lived experience in relation to contraceptive use with respect to your overall livelihood status? In terms of with whom you live, what is the source of your income and how many children you have?*

I do not know my age clearly. I have two children; the age of the first one is I guess a little more than ten year and my second child is about five years. I have started contraceptive method use after I gave my first birth. I used for three years and got pregnant my second one.. I am on contraceptive method as of a year for my second child up to now. I live with my husband and two children in our house. Our livelihood is problematic. We have critical food shortage in our house. We purchase on daily base as we got money to do so. The reason is that we have very small plot of land to be cultivated. Our livelihood was mainly dependent on what we got from our small portion of land and engagement in trading. The trading part had been supported us in the past but this year it did not work. I can say that our livelihood is just on the mercy of our Lord (በእግዝአብሄር ጸጋ ነው ያለነው).

*What is your experience about education both yourself and your children?*

With respect to education in our family, my first son is now attending his grade one schooling. He began his schooling late. Had he begun it, this time he would attain grade three. He was with his grandparents in his early age that was why he couldn’t began his schooling earlier. My second child is too young to schooling. She will start when her age is right for schooling. *What is your experience and intention with female education?*  I have all the determination to educate her and offer all supports at my capacity with the will of my God. I don’t want her to be as illiterate as I am. I am ignorant for many things as I was not educated. I guess this has prohibited me from many pleasant life affairs.

*What is your experience related to attending various meetings and training at kebele, community and other social affairs?* I have no experience in attending meeting so far but I only visit the health post on regular schedule to collect my contraceptive method.

*What is your lived experience related to your health situation?*  I had never experienced sickness formerly but since I gave birth to my second child I felt abdominal discomfort and pain. I sought medical help and now I am okay. My children are healthy and okay in the grace and mercy of my Lord. When any of our family members feel sick, we visit the health center. *Tell us your experience related to visiting market and other social gathering.* I go to market daily as we have to purchase food from market on daily basis. In the past I also sell flour. During that time I spend money from my pocket for domestic use but now I receive money from my husband to purchase food. *What do you do at your decision level by yourself without consulting your husband?*  I have nothing to do by myself without the knowledge of my husband. We have very small piece of land which does not need my involvement. The size of the land is not beyond the capacity of my husband.

*What is your lived experience in relation to contraceptive use and your livelihood in general?* *What primarily initiated you to use contraceptive service?*

Look…. We are poor. We have scanty things. If we keep on bearing as many children as we can do, we have nothing toffer them. We have no money, no land. On top of that if I keep on bearing children in uncontrolled manner, I will be harmed. I, therefore, started to use the method cognizant of the situations I mentioned above. *Where from you have heard about contraceptive services for the first time?* I heard from the government health institution, the health post. The health extension workers taught us about it. Then I started to use injectable contraceptive method since then. *What methods other than the method you use do you know?* I know methods such as oral contraceptive pill, other long acting methods such as loop and surgical contraceptive. But my preference is injectable method and so long it has suited for me. I do not see my monthly period (menstrual bleeding). *Who remind you about your contraceptive appointment? Does your husband remind you?*  My husband doesn’t know the appointment date. I count the date and visit to the health post when it is on. *Does your husband aware about your contraceptive use?* Yes he does. *What is your lived experience about your livelihood and health status since you have started using contraceptive method?* With regard to my health status, as I have informed you earlier I am okay as of the time I got sick last year. No special health problem I came across. But with regard to my economic status, there is no as such new attributes I can tell you. When I say this, we have no adequate resources. Our land is very small which cannot feed us from year to year. Consequently, our family is registered beneficiary from the safety net program. The safety net program provided us with one cow. That cow gave us one lamb which we sold to purchase food for our family. We are really poor family even when we compare to most of our neighbours. It is difficult to reveal whether contraceptive use has contributed to the improvement of our family economic status or not because of our poor basis. But, what I cannot deny is if I haven’t been on contraceptive method, our status would have been further deteriorated than this. *What is your feeling about not being pregnant at this moment?* I feel very happy for not being pregnant at this moment. You see, being pregnant and giving birth is not only consumes your time but also most of your energy, economy and many other issues. On top of living in such abject poverty, having more children mean being more crazy. Therefore, both I and my husband are really happy for that as we have done this purposely. With respect to the benefits and harms related to contraceptive method, for me the benefits are out ways it’s harm. I have minor gastric discomfort. When I compare this with the benefits I obtained from the contraceptive method, it is indeed not comparable.

*What do you suggest for those eligible women who are not currently using contraceptive services?* My message to them is clear. I will tell them that contraceptive service is useful. It helps women to delay unwanted pregnancies, therefore, urge them to use the services.

III # 7

District: Boricha

Kebele: Korangoge

Age: 30

Education: 7

# of children: 5

Year of contraceptive use: 4

*What is your live experience in terms of your livelihood, who with you live, number of children you have and what major life challenges you have in your family?*

I have a married woman live with my husband and five children together. My husband doesn’t have other wives than me. Our major source of livelihood is agriculture. Our major life challenge is lack of resources during summer time. We mostly went out of food stock at this time and hardly get money to purchase food items from market. *What is your experience to education in general and female education in particular?* *Do you send your children to school?* I used to attend schooling up to grade 7 before I dropped it for marriage. I send my children to school. I believe that education is one of the important instruments shaping the future of our children. Out of five children I have three are females. My older daughter was in school but she has dropped it for marriage without my knowledge. They fooled her and took her for marriage. I was totally in disagreement about that marriage but lacked strength to put an end on the marriage. This very young daughter was end up in such untimely marriage as they fooled her by telling her to give us huge money in terms of dowry. They said that to give me, the mother 3000 ETB, the father and other relatives culturally accepted standard of cloths and for her many cloths and other gifts. She wrote these points on letter and left the home for marriage. One of the problems in our local culture with respect to marriage is the involvement of outsiders. Those brokers without the knowledge of parents cheat young girls. That was how I lost my daughter. I would never let her marry at this age. My plan was to educate her to the level I could do.

Although I know the effect of early marriage and marriage without the consent of parents for young girl like my daughter, I was not in position to stop such act for the fact that our Sidama culture did not allow me to do so. For me it was not customary to directly go to the son in-law. I did try others to got to her and talk to her about the unlawfulness of the marriage but those people were not willing to convey my message. My husband was furious on the incident and wanted to take the matter to the court but local elders did not allow him as well.

*What is your experience about attending meetings in your locality?*  I work as a trained traditional birth attendant previously. Therefore, I attend meetings in the kebele frequently. I participated in health education meeting on child health and breast feeding. I gained knowledge about what exclusive breast feeding and supplementary feeding thereafter. I also attended education session on family planning, the importance of family planning, different methods available when to begin contraceptive method use. Based on the information I started using it for the last seven years. I can stop when desire comes for more children. But at this time, I am not sure whether to have more children or not. I receive my contraceptive method from the health post. I visit health posts for service use and attend meetings and educational sessions as they invite me. I also bring monthly report on woman who I attended on their labor to the health post. So, we have close link with the health post.

*What is your feeling about your health status? Do you have any health problem at this time?* I have no major health problems medical recognized but I feel abdominal discomfort and cramping pain which may be intestinal parasitosis. *What about the health status of your children?* My children are pretty well. None of them ever has visited health institution for medical reason except for preventive health services. With my small exposure to health information, I do care for them. I provide them varieties of food. I grow vegetables such as cabbage, carrot, potato, pumpkin etc… and give them these on top of their other food staff. *Where do you take if any of your family members get sick?* First, I take them to the health post. At health post they provide treatment to uncomplicated malaria. If the condition is beyond the health post, I take them to the health center.

*What is your experience in terms of decision-making at household level with respect to go out to market or other social gatherings?* I always seek his permission or I have to notify him before I go out to anywhere. If I fail to do so, he will be angry when I come back. I go to market twice a week: Wednesday and Saturday.

*What was the reason that motivated you to use contraceptive method?* When I saw emaciated and starved children born in close gaps and the harms their mothers faced, I recognized that if I continue in similar way, the same would face me. Therefore, I have started contraceptive method use to escape from such problems. *Where from or from whom you first heard about contraceptive services?* First, I heard information about contraceptive service in the church from the health extension workers. The health extension workers as a strategy to enhance access to the information for family planning have been using many community gathering places including the church. *What is your experience in contraceptive method use dynamics? Have you ever switched from one contraceptive method to another?*  Yes, I did. First I started to use oral contraceptive pill and then shifted to the injectable one. *What methods other than the ones you used do you know?*  I know other contraceptive methods such as surgical implants protect pregnancy for three and four years.

*What is your live experience related to your livelihood and health status since you began to use contraceptive methods? Tell us in terms of any change: either improvement or deterioration and how?*

We have got enormous improvement in our general livelihood. If to put in a very simple way, before contraceptive service use, our life was messy, untidy and full of bad odors. We were forced to pass our nights in bed richly socked with children urine. As of the time I started to use contraceptive service, our cleanliness improved, we have clean and better night cloths, new blanket not ever soaked by children urine. We have got new cloths for outside of our home. *What is your feeling if so far you haven’t been on contraceptive services?* If I haven’t been on contraceptive services so far mean for me to have more children than what I have now. I easily can say that I would have been broken/ deteriorated. You see…, my last child is now seven years old. Women who were not on contraceptive methods have added at least two children since I gave my last birth. For me being able to postpone these two unwanted births are considered a big relief on my life. I feel comfortable and safe this time. *What do you feel as you are not pregnant now?*  At this time, I have just discontinued using contraceptive services. In our Sidama culture, if the number of sons is less, people feel discomforted or not ease. And I have spaced for the last seven years , I want to have more child now. I feel I am pregnant now. I did this in consultation with my husband. He must know it for the reason that if anything happens, he is the one to give care for me. *What is your husband’s feeling on your contraceptive use and now its discontinuation?* He is happy. I did all in consultation with him. Nothing is new for him. Over all my opinion towards contraceptive service is great. It has enabled me to take adequate rest and improve both my health and income status. We became capable of planning the number of our children in relation to our economic status.

*What message do you convey to your neighbour women who are never used contraceptive methods?* It is my routine duty to tell them the benefits of contraceptive methods. I tell them if they use contraceptive method, they can plan the number and time when and how many children to have. One of my neighbour women, despite my telling, she continued to bear children looking for the male. After having many children, now she has started to use it.

III # 8

District: Boricha

Kebele: Korangoge

Age: 30

Education: cannot read and write

# of children: 5

Years of contraceptive use: 5

*What is your live experience in terms of your livelihood, who with you lives, number of children you have and what major life challenges you have in your family?*

I live with my husband and our five children together in our house. I have experienced no problems in relation to contraceptive use but have some problems related to food. What I mean here is we have no enough food to eat. Our source of livelihood is agriculture. *What is your educational experience including your own, your children in general and female children in particular.* I am un educated woman but determined to send my children to school. My first son has completed his secondary school education but not succeeded. He wanted to re-sit for the exam but unable to do so this years as he was sick in meantime. One of my daughters is grade seven this year and actively attending her schooling.

*What is your experience in attending various meetings and social affairs in your community? Have you ever attended meetings?*

I have attended a meeting organized by Goal Sdama five years back. The topics of the meeting were on births and food handling. They taught about how to handle food and prevent it from contamination. They shared for us the importance of personal hygiene while preparing food. If we fail to wash our hands, various contaminants can easily spoil foods and cause diseases in our family. They covered about saving and economic use of agricultural production at our households. They also informed about how to feed different food items we produce and also diversify our agricultural production in order to maintain our health status.

*Have you ever talk to the health extension worker?*  Yes I do but it was not in the form of meetings. When I go to collecting my contraceptive method, I discuss with the health extension worker about how to continue on the service and any challenge or problem I anticipate. The health extension workers also informed me about how to handle children, about child spacing etc…. *What is your lived experience about your health status, and that of your family members?*  So far, I haven’t come across serious health problems but we have chronic nutritional problems. We have never got balanced and diversified food. We mainly feed on “kocho” food processed from false banana or locally called ‘inset’. We have no enough cereal crops. Sometimes we purchase such food items from the market when we get money. No medically noticed health problems. With regard to my children health status, after the health post started in our kebele no health problems found on my children. Firstly, I use contraceptive method and spaced pregnancy and secondly, I took all my children born after the health post commenced function for vaccination. Except one all others are doing well and growing well. My one child is following treatment at Fullasa health center. The health professionals told us that he has anemia. He was transfused with blood and now regularly taking medication. Whenever my children get sick, I take them either to the health post or health center. In case the medical condition is beyond the health center, I take them to Yirgalem hospital or Hawassa University referral hospital. Before the health extension worker informed us where to use health services, we took sick family members to various places such us witch crafts and traditional healers.

What is your experience with regard to your movement outside your home? Who decide on your movement to various places? I go anywhere when I want to go with the prior notification to my husband. It is difficult for me to do so without his knowledge. We first discuss on the matter and then I go. I usually go to market twice a week (Wednesday and Saturday). When some urgent desires anticipate, I go as the demand comes to the nearby small market. With respect to income/ resource mobilization either for my family affair or other social affairs, I never do alone. I present the issue to my husband and we jointly decide on how to expend. When I bring the matter to my husband’s attention, he either agrees or disagrees. I carry out when he permits. Otherwise, I never do it alone.

*What is your lived experience in terms of contraceptive use, when you started it, what really initiated you to do so and where from you heard about the information and which method s you have used so far?*

I started contraceptive method immediately after my first child birth. I used oral contraceptive pill from Leku health center. This was long before a health center at Yirba established. Then I have shifted to the injectable method at Yirba health center. I was motivated to use the method as I have recognized that my economic status is not enough to have more children. When my first child reaches to take more food, where from can I offer him, and I saw that my breast milk was getting less. My physical condition was also getting worse when I saw it critically. If I keep on getting pregnant again, I said, “I am judging a death statement on myself”. That was why I have started to use contraceptive services. *Where from or from whom you have heard about contraceptive method for the first time?* I heard from my husband for the first time. My husband was attended schooling up to grade nine earlier. He heard about contraceptive methods from his friends and shared with me about it. He not only shared the information merely but also requested me if I could use it. Then, I said if it is good, why not and then started to use. *Why you shifted from oral pills to injectable method?* I was okay with the pills initially but lately I got a bit emaciated and decided to shift to the injectable one. Otherwise I had no problem. *Do you know any other method than the one you have used?* Recently, Zinash, the health extension worker, has told me the availability of methods such as surgical implants, loops. But for me the injectable one is okay and I don’t want to switch to other method now. I have seen the implannon but have never seen the loop.

*Please share your lived experience with contraceptive use related benefits and or harm in your life with us.* Before contraceptive use, women got pregnant now and then. But those who have started to use contraceptive methods become able to postpone or avoid unplanned pregnancies. Now, everything is smooth. You are in position to decide when and how many children you want to have. That is why I say everything is cool. I haven’t experienced major problems that can be sited here. Lives before contraceptive service for women were full of challenges and mess. We went to the extent we fail to get single cloth. Not only that but we were not able to wash ourselves and our children as well. Now it is nice for our family as I use the services. *What do you feel as you are not pregnant this time?* I feel happy as I am not pregnant. Being pregnant interfere with many activities. You cannot do whatever you want to do when you are pregnant. Now I am free to fly everywhere. I can go here and there, work whatever I want to do. The difference of being pregnant and not pregnant is clear. *Is there any problem you came across while using contraceptive method?*  In past times I had no problem with both oral and injectable methods but since last July I experience heavy menstrual bleeding. I have visited health post for this problem and talked to the health extension worker. She gave me medication for this purpose and since the time I used the treatment the situation has improved. But I feel I am getting weaker and a bit underweight. *What about the health status of your family members?* They all are doing well. They perform their routines in proper manner. Those who go to work, they do so as those students go to school.

*What is your lived experience since you have started contraceptive service use in terms of livelihood improvement or deterioration?* Since the time I have started using contraceptive method, I have engaged in small trading and generate some income, thereby I support my husband. There reason is that contraceptive method has made me get time and freedom from closed pregnancies. He works as a carpenter on top of his agricultural activities. *What is your husband’s feeling as you are not pregnant at this time?* He is happy as he was the one who brought the information of contraceptive service and initiated me to use it. *What really change of life you can tell us about contraceptive method has created for you?* There are lots of changes in this regard. Some are already stated above. But to substantiate what I have mentioned, I got time to prepare food in time and feed on time. I have better time to balance between work and rest. Therefore, I get better rest now. I am clean now as I have time to keep my personal hygiene. There are plenty of benefits that contraceptive service has created in my life.

*What is your opinion about those your neighbour women who have never used contraceptive service?* I keep on informing them about the benefits of contraceptive services. I say to them that the service will enable you to space your pregnancies and child births so that you will get enough time to spare for other personal and family affairs. I also have disclosed that contraceptive method will enable you to be clean and tidy. You will get time to feed well and maintain your health properly. Some of them have just started using the service.

III #9

District: Boricha

Kebele: Korangoge

Age: 30

Education: 7

# of children: 4

Year of contraceptive services use: 5

*What is your live experience in terms of your livelihood, who with you lives, number of children you have and what major life challenges you have in your family?*

I live in our house with my husband and our four children. One of the leading challenges I experience is lack of water. I born and grown up in Yirgalem, which is known for its ample availability of water. Since I moved to this area, what I couldn’t accustom and so far failed to seek remedy is water scarcity. When I went too far rural area out of my residence, I drunk water and unfortunately caught by diarrhea. Our livelihood is based on revenue we generate from agricultural activities and trading. We sell bread and tea in the local market by means of which we generate income for our family expenses.

*What is your lived experience in relation to education in general and female education in particular? Tell us about your education experience and intention to teach your children with emphasis to your daughters’ education.*

With respect to my educational status, I was attended schooling up to grade seven. I dropped out schooling as influenced by parents and ended in marriage. I am aware about educating my children and dedicated to support them in their entire educational career in my capacity. Both my son and daughter are attending school. *What about the experience of your daughter in engaging domestic work after school? How this can be explained in terms of comparing your son and daughter?* I never request my daughter to support me in domestic work leaving her school but she helps me in her spare time. She does it in the morning if her shift is afternoon and vice versa. In the evening she studies together with her brother.

*What is your experience in attending any training, meeting or social gatherings in your locality?* Yes I have some experiences in attending meetings. The first meeting was with a woman from Goal Sidama. She taught us about how to prevent malaria among pregnant women and young children by using insecticide treated bed net, draining stagnant water, environmental sanitation and personal hygiene. She taught also about the connection between poor personal hygiene and environmental sanitation with communicable diseases. Prevention of communicable disease is largely based on maintaining good personal hygiene and environmental sanitation. I also attended health education section about family planning. In this session I got information about the benefits of family planning in which it helps a couple to space pregnancies and child births and enable to plan the number of children and time when to have them. When a woman bear child in close interval (at least less than two years), the child couldn’t get adequate breast feeding, complementary food and general care. Moreover, the mother will be harmed by close child births through bleeding and lack of care including proper feeding.

*What is your lived experience in visiting health post and talking with the health extension workers?* I mostly visit to the health post and discuss with the health extension workers. The main purpose why I do so is to receive my contraceptive method (injectable). I also consult them when I experience some contraceptive method related side effects such as heavy menstrual bleeding. They gave me a tablet for the problem and advised to go to the health center for further help if the problem persists. Accordingly, I have visited the health center and the health center professionals done for me some examinations including urine test. Finally, they told me that there was no as such major problem and returned to my home. *What other health problems your have experienced?* As I have mentioned above, I had abdominal ache and diarrhea last time due to water contamination. Since then I have been using safe water by purchasing a five litre water for five ETB and now safe. With respect to nutritional status, we don’t have great problem in this regards. We eat various food items including corn, soya been, inset, drink milk and use milk products. I can say we use relatively balanced diet. *Did you come across any medical problem indicated by health professionals before?* In the past malaria was one of our major health problems. I was treated for malaria and got relief. Other health problems in the past were typhoid fever, giardiasis and other water and food contamination related problems. This time I am free from such problems as I was educated about the way how these infectious diseases are transmitted and can be prevented. My children’s health status is well. All of them grow in desirable manner. No health problems so far have been seen. If in case any health problem happens in my family, we will first go to the health post. The health post refers cases beyond their capacity to the health center.

*What are your lived experiences related to decision making in your family at your level in terms of going out of house to market and other places, mobilising resources and expending for family need?*

I am a merchant and have my own source of income from which I can expend when need arises. For example, my husband buys school materials and cloths for our children at the beginning of school year. I fill the need in the middle of academic year. I am the one who buy soap to wash their cloths. I am also the one who follows most of my children’s’ academic performances. I follow them while they do their homework, study and perform other school activities. I go to market and buy food items from my own pocket. But we get corn and inset from our garden. I have no problems to go out of my home. I have freedom to do so. I frequently go to market as I am a merchant. I go to health post and other community gatherings freely. I do not ask my husband for minor expenses as I can cover from my personal income. You see… it is not good always to depend on someone’s pocket as you can generate by your own at least some.

*What is your lived experience in relation to contraceptive use, the time when you started using it, what initiated your to use it and what change you have experienced since you have started using?*

I have started contraceptive method use after I born my first child. Then I used it for seven years and discontinues with the intention to have a female child. I consulted the health extension worker about it and discontinued. I first heard about the contraceptive method from the traditional birth attendant and community health agent before the health extension workers. My husband was worked as a community health agent and his experience has also helped me to use the services. Both I and my husband we also teach other members of the community to use contraceptive methods. Many women shared my experience and currently using the service. I use injectable contraceptive method since the beginning. I have never switched to other methods. I have never thought of changing the method I use. I found it comfortable and convenient for me and not certain about other methods. For example, if I want to use surgical implant, I feel it may not be convenient for me as I am a busy woman doing so many domestic works. Placement of surgical implant in upper arm may interfere with such activities. *Do you know any methods other than the one you use?* Yes I know. I know methods such as oral contraceptive pills and implants.

*What do you feel if you are not a contraceptive method user?*  I feel that I would have born as many children as possible in very close gap. The condition would harm both me and my children. My children might be undernourished, malnourished, emaciated, and skinny and in the worst case die of the problems. They could not feed breast milk properly and adequately. I might be either died or disabled if I were not used contraceptive method from unregulated pregnancies and its related complications. *What is your feeling ass you are not pregnant now?* I feel happy and peaceful. I give good care and support for my children. I am free from pregnancy as a result easily go where ever I want to go and generate income for my family. Everything is okay for me now. I feel good. My health status is nice. I have regular menstrual flow. No fear of pregnancy and its related burden in general. No bleeding following child birth and related health problems such as anemia, infection etc…. I feel extremely happy and thankful to my God. All family members are happy. We have planned our children number in relation to our economic status and health aspect. Both my children and my husband are really happy.

Contraceptive method use has prevented unplanned pregnancy and enabled me to participate in income generating activities. I am not pregnant, thus I easily walk to market and buy items to be sold in local market. I fully mobilise my energy to both domestic work and trade activities without hesitation. Pregnant woman can not to what I do freely. I have two daughters and two sons. I sent three of my four children to school. My first daughter is grade five, her successor is grade four and the third one is grade two. The last one will begin next year. Generally, I live happy and joyful life as a result of contraceptive method use. I really thank full to my Lord.

III #10

District: Wondogenet

Kebele: Wotera Gendo

Age: 20

Education: 2

# of children: 2

Year of contraceptive use:4

*What is your live experience in terms of your livelihood, who with you lives, number of children you have and what major life challenges you have in your family?*

I use contraceptive method currently. I live with my husband and two children in our house in this kebele. I have no as such major problems in household level. Our main source of livelihood is agriculture.

*What is your lived experience in terms of education? Tell us your educational experience whether you have ever attended school or attending it now, about your children education and more specifically your daughter’s education?*

With regard to my educational status, I attended to the level of grade two and dropped it out long ago. I send all my children to school including my daughter. I have one son and one daughter. I do not differentiate in between the male and female education. I send both of them to school and offer my support to both of them. I well recognize that female has to get educated to best fit in today’s world. I took lesson from my personal experience as I had done my schooling very well in the past; I would have obtained better position in society than this.

*What about your experience in attending various meetings in the kebele?* I have never attended any meeting.

*What is your lived experience about the health status of your and your children, what do you do when ever any family member gets sick, where do you go or take to?* I feel well and okay. I have no observable or felt health problems. I feed well relatively. With regards to the health status of my children, they are well, grow properly and have no notified health problems so far. They attend their school and play with their friends in neighbour and school. They feed well and act properly. My experience in looking remedy for health problem is, first I go or take to the health post and if the condition is beyond the health post then to the higher level health institutions ; the health center and hospital (Yirgalem or Hawassa referal). Other than the health institution, I never go or take to other places.

*What is your lives experience in terms of going out of your house to the market or other social gatherings and attending meetings? How often do you do these? And what is your experience in doing this by yourself?*

Here in our community it is a customary for a woman to get permission from her husband to go to anywhere. Likewise, I have to first secure permission from my husband before moving anywhere. I go to market twice in a week. In case if my husband is unhappy and failed to allow me to go to market, I never go. There are some activities I perform without requiring his blessing. These include washing clothes, cleaning house, cooking food, and garden cultivation. I can sell some of the garden vegetables which belong to me but I cannot sell my husband’s properties.

*What is your lived experience in relation to contraceptive use, the time when you started using it, what initiated your to use it and what change you have experienced since you have started using?*

I started contraceptive use four year back. I was initiated to use contraceptive method as observing the gaps between the two pregnancies I had. I got pregnant in less than a year time for my first child. He did not fed breast milk adequately. Failure to breast feed properly means for a child, unable to attain desirable level of growth and become weak. I saw this practically on top of my knowledge about it. Therefore, not to repeat similar mistake, I decided to take contraceptive methods and using it since the time I have started. *Where from or from whom you first heard about contraceptive services?*  I first heard from my neighbour called Beletu. I am using injectable method. I have never used methods other than the one I use now. *What other methods do you now than the one you use?*  I know surgical implants and loops. *What is your experience in terms of livelihood in general by comparing the time before and after contraceptive use? What difference you have experienced?*  The first issue is time issue. Before contraceptive use, I hardly get time to even wash clothes, and do other domestic works as mostly occupy with child care. But now in the era of contraceptive service, I do all my activities in plan. I budget my time to the activities waiting me to perform. If I have not in contraceptive method, my life would be miserable. Children would have been born in close frequencies, no enough things to feed them, no time to take care of them; they would be emaciated and malnourished, un health. I would also been sick, underweight and unhappy in my life. Contraceptive use has also enabled me to participate in generating my own income by herding animals such as sheep and cows. Whenever I have demand for my family, I sell cow products: milk and butter and expend for family need.

*What do you feel for not pregnant this time?* I feel happy and peace. I fell healthy. My husband is also feels happy for that I am not pregnant. He is convinced that unplanned pregnancies and child births affect both the health and economic status of a given family. He wants to handle properly the two children we have without over stretching his capacity. We both agreed in this idea and I continue using the method. *How do you recognize about the benefits and harms of contraceptive methods?* I know that contraceptives for me are beneficial. I have never experienced its harm. Since I started using it, I have never felt sick, thus the benefits in my case is obvious than the harm.

*What is your advice to those your neighbour women who are not on contraceptive methods currently?*  I approach them telling my story. I try to reveal what benefits I got by using contraceptive method both for me and my children. I will tell them that if a woman bear children in close gap both the mother and her children are affected in health, nutrition and many other economic and social aspects. I will also add to them that contraceptive method use improved the love between a husband and a wife as they get better time to know each other and the woman can please her husband better than before. So, I will emphasis in all these matters and try to convince them.

III#11

District: Wondogenet

Kebele: Watera gendo

Age: 30

Education: cannot write and read

# of children:7

Year of contraceptive service: 7

*What is your live experience in terms of your livelihood, who with you lives, number of children you have and what major life challenges you have in your family?*

I live with my family members (my husband and children) in our house together. I have seven children and currently using contraceptive method. With regards to livelihood problems, I have no as such major problems. And our livelihood is mainly depends on agriculture.

*What is your lived experience in terms of education? Tell us your educational experience whether you have ever attended school or attending it now, about your children education and more specifically your daughter’s education?*

With respect to my educational status, I have never attended school and therefore, unable to read and write. I am illiterate. My children are attending school. I not only send my male children but also my female children equally. I have two daughters both are attending their schooling.

*What about your experience in attending various meetings in the kebele?*  Yes, I attend meetings. The meetings I attended include session on HIV/AIDS prevention and control, breast feeding, latrine construction and utilisation, family planning and child spacing. I closely communicate with the health extension workers. I visit the health post to collect my contraceptive method and discuss with the health extension workers about any issue related with contraceptive method.

*What is your lived experience about the health status of your and your children, what do you do when ever any family member gets sick, where do you go or take to?* I feel well and no professionally notified health problems. My children are also doing well. They grow well and catch up regular patterns as compared to the neighbour children of their age. I haven’t faced major health problems in our family. We seek health services mostly from the public health institutions including the health post in our nearby vicinity.

*What is your experience in deciding by your own when you plan to go to market, health institutions and other social affairs?*

I go to market once in a week time and I do this with prior permission of my husband. I never go to anywhere without his knowledge. There is nothing mostly I do by my own decision. I do only taking care of domestic activities such as cooking food for the family, washing clothes, utensils and digging the garden. In the absence of my husband I do all household related decisions.

*What is your lived experience in relation to contraceptive use, the time when you started using it, what initiated your to use it and what change you have experienced since you have started using?*

As you can see I have already seven children. To have more children means to have more challenges. Knowing this situation, I preferred to take contraceptive method for better handle my children, to educate them and to nurture them. *Where from or who from you first heard about contraceptive services?* I first heard from the kebele chairperson about contraceptive method. I use injectable contraceptive method currently. I have never used other methods than this one. But I know contraceptive methods other than the one I use. I know oral contraceptive pills, surgical implants and loops.

*What is your experience in terms of change or difference in your livelihood before and after contraceptive services?* After contraceptive use I have no problem. I got many changes. I got many things which I was not previously. If I am not currently on contraceptive use, at this time I would have more than 10 children and I would either be sick, disabled or dead. My children would have been ill, malnourished and some of them might have been deceased. But, contraceptive method use has eased from such problems.

*What do you feel now as you are not pregnant?* I feel happy and peaceful. I feel joy. I am well and feel healthy. I am engaged in many domestic and outside activities which I could not do when pregnant. I have more time to spare for these activities as compared the time before. My husband is also happy as I am not pregnant this time. We do this in consulting each other. Since he knows my contraceptive use, he is happy about it. *How do you see the overall benefits and harms related to contraceptive method use? Which overweighs?* For me the benefits of contraceptive method use outweigh that of its side effects. I have never noticed major problems related to contraceptive use.

*What message do you share to your counterpart women in neighbourhood who are not using contraceptive services?*  Contraceptive methods enable user to space or totally stop unwanted or unplanned pregnancies and safe life for both mother and children. I tell them all the benefits of contraceptive services for both mothers and children. I tell her if she keeps on bearing children without gap, her children get emaciated, stunted, and unhealthy and may die due to health problems. Moreover, the mother herself will be badly harmed. I will tell her this way.

III #12

District: Wondo Genet

Kebele: Wotera Gendo

Age: 25

Education: cannot read and write

# of children: 4

Year of contraceptive use: 6

*What is your live experience in terms of your livelihood, who with you lives, number of children you have and what major life challenges you have in your family?*

I am a married lady live with my husband and four children on our house. Our main source of income is agriculture. We use a mixed type of agriculture which means both crop production and herding of animals. With regard to the patterns of our livelihood, there hasn’t been major problem as compared to our neighbours. *What is your educational experience at your family level, sending your children to school in general and female child education in particular?* I recognize the benefits of education to my family members. I know I haven’t attended schooling for the fact that my parents didn’t make me to see this world. This has created a sense of strong feeling to send my children to school. The good thing this time is that we have primary school at our kebele. Our children are not worried about walking long distance. Thus, we have convenience in sending them to school. I have one daughter and she is at school.

*What about your experience in attending various meetings in the kebele?*  Yes I have good experience in attending meetings and educational sessions at kebele and community levels. The topics so far I have participated include: about environmental sanitation (latrine construction and utilization), about family planning, personal hygiene and others. *How far you exploit/use the health post? What level of contact do you have with the health extension program*? I have good relation with the health extension workers. I usually go to the health post to get my contraceptive injections and also when get invited to attend meetings.

*What is your lived experience about the health status of your and your children, what do you do when ever any family member gets sick, where do you go or take to?*

With respect to my current health status, I am well and feel good. I don’t have any problem. My children are also doing well. There is no health problem notified by the health professionals. I had some problems before I have started contraceptive use. Before contraceptive use, I experienced close pregnancies and child births which had depleted my general wellness. I was weak then but since I have started using contraceptive method, I feel healthy. In case if any of our family members get sick, first we take him/her to the health post. If the medical condition found to be beyond the health post, we take to the health center and then to hospital.

*What is your experience about going out of your house to various social affairs, health institution, market etc… and who is deciding on these issues?*

I go to market twice a week and to the health post as need arise. But I must visit health post once in three months to get my contraceptive injection. With respect to who directly decide on my movement out of my house, we jointly do it but when I want to go, I have no problem. I do it by myself. My husband doesn’t create problems whether I tell him or not to go where ever I want to go. I have my own means of income. I have a cart that I get money from it and expend for expenses directly concern me. For example, I buy school materials (exercise books, pens, pencils, and clothes) to my children without requesting my husband.

*What is your lived experience in relation to contraceptive use, the time when you started using it, what initiated your to use it and what change you have experienced since you have started using?*

I first encouraged using contraceptive service as I had experienced burdens of closed pregnancies and child births. When I got information about the existence of means to get rid of such problems, I decided joyfully to commence the services. I do not want to bear such problem again. First I heard about contraceptive service from the health extension workers here in our kebele. *Which method do you use now? And why do you use this method?* I use injectable contraceptive method (depo-provera) and I do so as this method is convenient for me. *What other methods of contraceptive do you know other than the one you now use?* I know methods such as surgical implants, oral contraceptive pills, and loops. With respect of live change since I have started using contraceptive service, I have many pleasant issues happened in my life as of the time I have been on contraceptive. With most impressing issue, my marital relation has greatly improved. My husband disrespected me when I gave too frequent births and not in position to care for myself. As contraceptive service has allowed me avoid unplanned pregnancies and fairly use my time for family affairs and my personal life, now I am able to catch my husband’s eyes. Our life is now established in agreement and mutual respect and love. *What do you feel now as you are not pregnant now?*  Look, when you are pregnant you feel tight and discomforted. You are uncertain for the outcome and worried about what you can do for it. But now I am free from such problem and feel healthy, peace and happy. I do whatever I wanted to do without limitation as long as I have time and energy. Here contraceptive method has enabled me to engage in such activities by freeing me from pregnancies and child births. My not being pregnant has also made my husband feel happy and love me instead of hating me formerly. He came to the level that reminding me my appointment date. In general, I have experienced the benefits of contraceptive method enormously and its harm is negligible. My message to the current non user neighbour women is that “learn from my experience”. I was hated by my husband but now as a result of this method improved the relation, therefore instead of bearing children closely and too frequently, try to space and improve your livelihood. I emphasis for them to recognise the benefits of contraceptive methods use with that of unplanned / unregulated fertility are great when compared to the risks/side effects.

III# 13

District: Wondo Genet

Kebele: Wotera Gendo

Age: 25

Education: 5

# of children: 3

Years of contraceptive use: 3

*What is your live experience in terms of your livelihood, who with you lives, number of children you have and what major life challenges you have in your family?*

Our main source of income or livelihood is agriculture. We get need for survival from this sector. I live with my family members (my husband and our three children) together in our house. With regard to the life challenge, I noticed no special problem. We lead normal local life as average people of our surrounding.

*What is your experience with regard to education in your family including yours and your children?*

With respect to my educational status, I had never attended schooling. My parents did not give me such opportunity. Our childhood life was dark time where many of our parents had no courageous or belief about female education. Like most of my age female children then, I remained illiterate. But now I send all my children to school including my daughter. I understand the benefits of education to all human kind and my daughter. I never want my daughter to lead a life as me. I wish her attain better education and lead better life.

*What about your experience in attending various meetings in the kebele?*

I have no experience in attending meeting at kebele level but I go to the health post to get my contraceptive injection. Except for this service, I never went to the health post either.

*What is your lived experience about the health status of your and your children, what do you do when ever any family member gets sick, where do you go or take to?*

I am okay now. No any professional notified health problems so far. I feed well in my capacity and lead normal daily life. My children are also in good health status. *What do you mean by good health status?* I mean that they eat and drink well, they go to school without interruption and they have no obvious health problems or signs of sickness. They grow well to their age. They do play with their friends in neighbour. In case when a family member got sick, first I take them to the health post and when the health extension worker instruct us to go to higher level health institutions such as health center, I take there. I never take to other place such as traditional healers.

*What is your experience about going out of your house to various social affairs, health institution, market etc… and who is deciding on these issues? What is your experience in other domestic decisions?*

I go to market once in a week. I do this with prior notification to my husband and securing permission from him. I never go to market or any place without his knowledge and permission. *What do you do by your capacity without informing your husband?*  I do domestic activities such as food preparation, feeding my children, cleaning of house and other similar activities at house.

*What is your lived experience in relation to contraceptive use, the time when you started using it, what initiated your to use it and what change you have experienced since you have started using?*

I gave two consecutive births in close gaps and challenged to handle them. I got lesson from that and decided to take contraceptive method. I understood that contraceptive service is a means which can ease me from such challenges. *Where from or from whom you first heard about family planning/contraceptive methods?* I first heard about contraceptive service from the health extension workers. I use an injectable method. I have never used methods other than the injectable one. *Do you know the existence of other contraceptive methods than the one which you use?*  Yes, I know methods such as surgical implants and loop. *What change you have experienced since you started using contraceptive service?* Before contraceptive use I was isolated from other people. Many people did not approach me as they undermined me for the status I had. My repeated and close pregnancies and births ruined my personal, economic and social status, thus they undermined me. I feel happy and comfortable for not being pregnant this time. You see…, pregnancy itself is a problem. It hampers your involvement from various household and social activities. You cannot actively involve in many duties. Now I am not pregnant and do many things. I also walk long distance to market and buy items needed for my family members. All my family health status is well. Contraceptive use has created opportunities for me to involve in income generating activities. By doing so I boost my family income. I plant garden vegetables such as potato, inset and maize. On top of my husband’s effort, I strongly participated in agricultural activities and improved production.. I generally feel very happy as I am not pregnant this time.

When to put the balance between the benefits and side effects of contraceptive method, for me, the benefits is wider and deeper than its side effects. I say contraceptive use is beneficial for me in many perspectives.

*What do you say for your neighbour women who are not currently use contraceptive services and subjected to bear children in close gap?* I will tell them that contraceptive method is a remedy to escape from problems related to unplanned pregnancies. I will also present my life experience as good example for them.

III # 14

District: Boricha

Kebele: Dilarife

Age: 27

Education: 7

# of children: 4

Year of contraceptive service: 12

*What is your live experience in terms of your livelihood, who with you lives, number of children you have and what major life challenges you have in your family?*

I am seventh grade completed and 27 years old now. I live with my four children and my husband together in our house in this kebele. I have been on contraceptive method use since 1994 E.C. Our main source of livelihood is agriculture. With regards to challenges of livelihood, we have some problems. After the time we have this four children, our livelihood is becoming compromised. We have no enough food, and other livelihood inputs thus, I feel discomfort now.

*What is your lived experience in terms of education, inclusive of your children and yours?*

I am seventh grade completed and dropped schooling long ago. My children are in school. I have four children and three of them are currently attending school. With regard to female education in our family, from the two daughters I have, one is in school now. How do you see female education? I have heard many good things about female education. I am really convinced to send my daughters to school. I learned that female education is important means to improve women status. Therefore, I not only support female education but seriously dedicated to see their fruit. I know that there are many obstacles for female education such as marriage by abduction. But I tell my daughter to be strong and deny any request related to young age marriage. I have big vision to see the fruits my daughter education.

*What about your experience in attending various meetings in the kebele?*

Yes I have experience in attending meetings and education sessions in the kebele. The meeting and educational sessions I have attended were on the following topics: about personal hygiene, environmental sanitation, harmful traditional practices, reproductive health and etc…. *Do you have chance to contact the health extension workers?*  Yes I do. I visit the health post to receive my contraceptive service regularly and when they call me to participate in various meetings and educational sessions.

*What is your lived experience about the health status of your and your children, what do you do when ever any family member gets sick, where do you go or take to?*

I have no major health problems but I feel dizziness sometimes. When I feel so, I go to the health center and take treatment*.* They told me that I have anemia. I feel that is this due to the contraceptive method I use, but I do not usually see my period. *How about your children health status and growth patterns?* They grow in very good manner. None of them has undernourished or underweight. They have no medical notified health problems so far. In case if any of the family member get sick, we take to the health center.

*What is your experience about going out of your house to various social affairs, health institution, market etc… and who is deciding on these issues? What is your experience in other domestic decisions?*

I go to church with my husband but I go to market either alone or sometimes with him. I go to market ones in a week. The market is at Darara, which is at least one hour walk distance from here. I tell to my husband before I go to market. *What experience do you have in decision-making by yourself at household level?* I do many domestic works by myself such as food preparation. Every school expenses for our children were done by my husband. He buys clothes, and other school materials for them.

*What is your lived experience in relation to contraceptive use, the time when you started using it, what initiated your to use it and what change you have experienced since you have started using?*

I was motivated to take contraceptive services because I learned the benefits of contraceptive services in enabling a woman to postpone pregnancy which she is not desirous or planned. I got insight about contraceptive service helps both the mother and her child to be healthy. *Where from or whom from your first heard about contraceptive services?* First I heard from a project called ESHE. This project was teaching people about contraceptive services in our kebele. I heard about it in 1996 E.C. Since that time on ward I decided to use the service and now I am taking depo-provera, injectable contraceptive method). I have briefly used oral pills but it have caused massive bleeding and I switched back to depo-provera. *Do you know any other methods of contraceptive than the ones you have mentioned?*  Yes I know methods such as surgical implants and condom. *What change in your life have you observed/experienced since you have started using contraceptive service?*  If I hadn’t started using contraceptive method, this time I would have many children. I could not be in position to handle them and send them to school. Now, we all are well and healthy. If no contraceptive so far, it means that the level of poverty would be intense. The number of children easily would be more than eight this time. This means how much challenges for a fragile economy like mine. *What do you feel now as you are not pregnant due to contraceptive service use?* I feel very happy. I feel so because, I am free from the burden of pregnancy, no fear about the unlikely outcomes of it and have time to share for other activities than child care. My husband is too. I take the service in agreement with him. I told him about the benefits of the service and he joyfully supported the idea. In general, I have pleasant feeling about contr*ac*eptive services as it has many help for a woman like me, for children and community in one way or another. I so far accepted contraceptive service in its positive outcomes.

*What advices to you have for women who have never used contraceptive services?*  What I will do is to tell them about the concrete benefits of contraceptive services such as maternal and child health. It gives women better time to maintain their health and that of their children. So I will say to them, please see the benefits and get rid of the unplanned pregnancies’ outcomes in your lives.

III# 15

District Dale

Kebele: Ganne

Age: 32

# of children 4

Education: 5

Year of contraceptive service use: 3

*What is your lived experience in relation to contraceptive use with respect to your overall livelihood status? In terms of with whom you live, what is the source of your income and how many children you have?*

I am on contraceptive use currently and I have four children which I born in well-spaced manner. There was gap between my first and second births about four years. Between the second and third children the gap was seven years. I am using contraceptive service and encouraging non users to use. *What life challenges do you experience at your household level?*  Our life is beautiful. My husband is a farmer, I am a merchant and our children are students. Our children after school time also help us in domestic work and occasionally the bigger ones help in market activities as well. I sell prepared food on the market day and also sell many items. My older child works as a barber and generate some money.

*What is your lived experience in participating various meetings or community education sessions?*

I serve as community mobilizer for women in my locality. I disseminate information about them in case when the kebele need them to attend meetings. I also organize sessions for health extension workers when they want to teach women in health extension packages. I also work as messenger for women federation in our kebele. These positions have given me to easily participate in most community affairs as a facilitator. *What is your experience in relation to attending training session or meeting about reproductive health at health post level or kebele level?* To my knowledge I haven’t attended such session collaboratively. But I individually visit health post to receive contraceptive service. I used implanon for three years and reinserted again when the former one has done its functionality duration.

*What is your lived experience about the health status of your and your children, what do you do when ever any family member gets sick, where do you go or take to?* I feel healthy and okay. Nothing special thing or any medically notified health problems I have this time. I feel I have standard weight to my age and height. I eat well and do my routines uninterruptedly. With regards to my children’s health status, all have grown well. To my knowledge I haven’t taken them to the health institution even for the common cold other than vaccination program. *What is the secret behind for the healthy upbringing of your children without major health problems?* There is nothing special we have done but I fed my children very well and all of them have received vaccination against child hood and vaccine preventable diseases during their young child age. They have followed growth monitoring and none of them has shown any sign of malnutrition. I also tried to stick to the health information given by various media and health extension workers in order to prevent health problems. *Where do you take if any family members get sick?* We will take to the health institutions. First I take them to the health post and if the health post tell us the case is beyond their capacity, I will take to Yirgalem hospital.

*What is your experience about going out of your house to various social affairs, health institution, market etc… and who is deciding on these issues? What is your experience in other domestic decisions?*

We have an established experience in reaching on decisions including any movement outside our house. When I plan to go to health institution, market or any other social gatherings, first I present the issue to our family gatherings and discuss on the matter and reach on consensus. Then, I go where ever I want to do so. *What has derived you to think of such collaborative decisions in your family?* The idea of such collaborative /joint decision at household level has developed from various points. We heard about it from local radio broadcast about family planning and saving and credit. Moreover, the current one-to-five organization in our kebele has given nice opportunity to try such approach in our family. We strongly believe that for any development joint decision is spring board. We took the experience at kebele level to our household level and using this way to pass any decision. *Is there nothing you alone decide?* Yes I have. What I do usually is that first I initiate thinking at my mind and send to my heart. In the heart it was said to have seven openings. There, the idea circulate and mature. After that I confidentially carry it out or perform. *Who decide on expenses including school fees?* Most of the time, as I said earlier, my husband and I decide together in consultation with our children. But the primary one is my husband as head of the household. Sometimes children are shy to ask their father for some school expenses and they easily approach me and I give them as long as I have money at hand. *Were there any occasion that you either denied the decision of your husband or modified it?* There were no such circumstances but what usually we do is we discuss and I raise my concern in the process of decision if I see any discomfort in expense.

*What is your lived experience in relation to contraceptive use, the time when you started using it, what initiated your to use it and what change you have experienced since you have started using?*

I first started contraceptive use in 1988 E.C. I married at very young age. I was only 13 years old. My marriage was not consensual but I was abducted. As I was too young to be pregnant, I decided using contraceptive for fear of pregnancy. At that time injectable contraceptive method was not offered for null para woman. I first heard about contraceptive method from my brother. My brother was educated and when he saw that I married at that young age, he was worried about what could happen. As I was too young to be pregnant, he terrified about the outcome of pregnancy at that age and consulted a health professional. Then the health professional informed my brother the possibility to take contraceptive method and postpone pregnancy until I will be matured. Even though he was not in agreement with my marriage at that way but as a matter of local culture influence, he could not abort it. Thus, I used oral contraceptive pills for three years and when I feel that I was mature to get pregnant; I stopped taking the service and got pregnant. After I gave birth for my first child, I took him for vaccination to the health center and the health professional at the center gave me injectable contraceptive method. I used the injectable for ten years and then planned to get pregnant and stopped it. Then I gave birth to my second child. After that when the surgical implant introduced, I used it. I used it for three years and when its functionality time due, I removed the former one and re-inserted the second cycle. *Why you have switched from the injectable to the implant?* Both the oral pills and injectable methods were convenient for me but the reason why I switched to the current implant is that this method once is inserted in the upper arm, it works for three years. Therefore, it doesn’t require you to remember appointment every month or three months and not to go to the health posts frequently. That is why I chose it.

*What is your intention to have more children?* I have no concrete idea on this but with the time I may think of it. What I know at this time is to continue on the method I am using. *What is your feeling about the life experience and changes observed since you have been on contraceptive methods and what could happened in contrary if you were not on the services?* In simple words I can say that if I were not on contraceptive service, leave alone my existence, you may not find my burial place this time. As you could understand from my life experience, I married at that very young age and got pregnant immediately, I might be dead or disabled. So, for me the benefits of contraceptive method are beyond expectation. Not only it has saved my life but created real opportunities for my children to be better cared. I care for them, send them to school and nurture them in a way that they are competent with their neighbour children. Both my livelihood and that of my family has come in proper manner. If I were not using, we could be in abject poverty, my children could not attend school, and they might have no enough to eat.

What is your feeling about you are not pregnant now? I feel very happy. *What made you to feel happy?*  I feel happy because I see the difference in overall livelihoods and health status of my neighbours who are not using contraceptive methods. When I tell them about the benefits of contraceptive service, they were confronting me by saying that it is not good, causes some health problems and so on. Look, it is about ten years since I gave my last birth. But the non-user since then has given four additional births. With these they are trapped in poverty, they have no enough to eat for themselves and give for their children. Some of their children area highly emaciated and some were died. I see the gaps between my life and theirs. I really cry about their lives. What surprises me about this service is that it is free. If we want to buy from market by paying from our pocket, how expensive it could be. *What is your husband’s feeling about you are not pregnant this time?* Somehow he wants to have more children but I convince him about the disadvantage of having many children. I tell him in detail about the burden related with pregnancy and child bearing. Having one child, at least take more than three year time from your involvement in various economic activities. I say to him that let’s first take care of our existing children and up bring them. If I get pregnant, I stop all the activities I do now. All household expenses will require you to cover, thus you will be overburdened. If one child get sick and seek high level medical care, the expense may not be simple. It may request to the level of more than a thousand birr. Therefore, with all these information, he is happy and also we can have more child when we feel we are ready so both psychologically and economically.

*How do you explain contraceptive service in general either good or bad, why?* Contraceptive service is beautiful for me. I say so because it has created many opportunities for me and for other women who use it. Having close pregnancies and births means great burden, poverty, weakness, and socially under recognized. *What do you do for women who are not currently on contraceptive use?* The problem in our area is that many women start contraceptive service use but lack perseverance. They easily stop whenever they see minor side effect. I have a neighbour who was on implanon but immediately removed in two months of its insertion and then shifted to injectable. She also stopped injectable and got pregnant. What I tell them is to use the services continuously and if they face some side effects; instead of stopping the service to share the problems to the health worker and seek advice from them.

III # 16

District: Dale

Kebele: Degara

Age: 30

Education: 10+2
 # of children: 4

Year of contraceptive use:

*What is your lived experience in relation to contraceptive use with respect to your overall livelihood status? In terms of with whom you live, what is the source of your income and how many children you have?*

I live with my husband and four children together. We have no major livelihood problems. I married him when I was grade 10. After that while we are living together asked my husband if he could send me to school or not as I have strong desire to complete my secondary education. He accepted my request and let me continue my diploma level 3 educations in gender and development studies. Now I am a diploma holder. Our sources of income are mixed. My husband is a principal in Degara public primary school and earns monthly salary. I work in the agriculture activities in our garden. We generate income from these two sources and use for our family. Three of our children are attending school at Yirgale private school called Admas primary school. My first child is 12 years old; his successor is a female one aged seven year. *When you sent your children to Yirgale, who care for them and how do you accommodate them? Is it not difficult for you to manage?* I organized for them how to care. We have relatives living with them and we pay for the school which is not beyond our capacity. We have enough to teach them as we generate income from various means. *You sent all your three female children to school. How you overcome the tradition of not sending all females to school?* Both my husband and I well understand the equality of male and female. Therefore, without any compromise or challenge in our level, we sent them to school. As I am graduated from gender study, I well recognize that both male and female have to get equal access in all life endeavours.

*What is your experience in attending meeting or education sessions related to health and reproductive health at kebele level?* I have never been attended such sessions so far. *Have you ever discussed or communicated with the health extension workers?* Yes I frequently contact them. I closely work with them as a community volunteer to mobilize women in our locality. I share information with women on various health program directed by the health extension workers.

*What is your lived experience about the health status of your and your children, what do you do when ever any family member gets sick, where do you go or take to?*

I feel well. I have no problem. I live happy and decent life. We respect each other and love each other. So, our life is cool. I have good relation with my neighbours. I participate in social affairs in our surrounding feed well and guess I have normal body weight. Moreover I have no medically notified health problems. *What about your children’s health status?*  All of my children are doing well. They have come properly to their age. They all received child hood vaccination and monitored for their growth when they were young children.

*What is your experience about going out of your house to various social affairs, health institution, market etc… and who is deciding on these issues? What is your experience in other domestic decisions?*

With regard to physical movement outside home, I have no problem of doing so. We have smooth relation with my husband and good trust between each other. That was how I went to tertiary level education and completed it. In case of domestic decisions, we jointly make it. We discuss what to do for our children’s’ in their schooling and then buy all necessary inputs such as cloths, exercise books, pens, pencils etc…. We also decide household expenses together. He collects salary and come to home then we list expenses and execute it together.

*What is your lived experience in relation to contraceptive use, the time when you started using it, what initiated your to use it and what change you have experienced since you have started using?*

I get married in 1994 E.C. and gave birth to my first child in 1995. I started to use contraceptive service a year after my first birth. *What has derived you to use contraceptive services?* The first thing I decided using contraceptive service is a big concern for my health status. When I mean this: unregulated and unplanned pregnancies have much impact on maternal health. A mother will become very weak due to repeated pregnancies and child births. Sometimes massive bleeding happens during labour may threat the life of a mother. I had big dream to complete my education which I dropped due to marriage and to do this I was aware that if I get pregnant repeatedly, I could not. In order to avoid or space the pregnancy I must have used it and did it. I first used injectable method and remained using the same up to now. *What other contraceptive methods you know than the one which you use?*  I know methods such as pills, loop and surgical implants. *What life change you have experienced since the time you have started using contraceptive methods? Tell us the change you experienced either in improvement or deterioration by comparing with your none-use time and those women who are not yet using.*

My life situation in general is well. If I had never started using this method, I would have deprived of many privileges I obtained this time. As I have said earlier, I could never attend my secondary and tertiary school and unable to send my children to school. Moreover, I may not be as health as I feel now. If I want to put my livelihood situation with those who are not using the method, the difference is like the sky and the earth. *What is your feeling about you are not pregnant as the result of contraceptive use so as your husband?* I feel happy and joyful as I am not pregnant now and did it purposely. I feel very happy not only because I am not pregnant but the method has freed me to care for my children in better ways. In general our income has improved since I started using contraceptives. One thing is that I have time to work by myself in the agricultural filed and generate some income. My husband is a public employee earning monthly salary. We boost each other and our income is stable. If I have never used the method, this time I may have more than seven children. This means that it depletes our income and stretches our capacity. Therefore, the contribution of contraceptive use has double fold in improving our income status.

What do you do for women in your neighbour currently not taking contraceptive methods? I will inform them the benefits of contraceptive services. For a woman it has many help. It prevents unwanted pregnancies and births so that prevents untimely deaths and discomforts. I also emphasis that not only the benefits to mothers but also for children they born, the method provide better chance of survival. Thus, I will say please use and experience the benefits as I do.

#17

Woreda: Dale

Kebele: Degara

Age:

Education:

# of children: 4

Year of service use:

*What is your lived experience in relation to contraceptive use with respect to your overall livelihood status? In terms of with whom you live, what is the source of your income and how many children you have?*

With respect to my life style and livelihood, I live with my husband and we have four children (two sons and two daughters). We have various means of incomes. I am a teacher in public primary school consequently earn monthly salary. My husband is an evangelist in fully Gospel church. We have also ploughing land where we grow various plantation and crops. To mention some of these are: ‘inset’ (the false banana), coffee plants, maize and other vegetables. We have about two hectare land plot. We hire worker to process the inset into food item (kocho), collect coffee and sell it. We feed part of to and sell the remaining part. The income from salary, agriculture and other means, we use for our domestic expenses and our children’s school expenses.

*Educational experiences:*

Among the four children we have three of them are in school. We sent all of them to Leku town where we expect better education than the education at our kebele (the most rural one). Our third child is too young to school and is at home now. But we will directly send him to school when his age is proper to schooling. Out of the three children we have in school now, two of them are females. *What has encouraged you to send your female children to school as it was not the case in short past? And, how do you see the education of female and male in general and female education in particular? What does it contribute to one’s family?* Education in general is an important asset for everyone. Its importance is becoming more crucial and visible this time. Formerly many people in rural part of the country depend on land to be cultivated. Now a day, the land size in getting smaller and smaller to the level that it cannot carry in the manner it did before. With this recognition, we try to shape them from this time on ward to be educated and shape their ways forward. I highly value the female education. Unlike our times, we have good insight about sending our female children to school in order to compute with their male counterpart and lay down basis for their future life. My opinion in educating female children is strongly based with the assumption that female education has overarching and fast expansive benefits in households and community at large. A female is the one who closely takes responsibilities at her parent’s house next to her mother and her education will enable her to better handle her prospective house.

*What is your lived experience related to your physical movement outside of your house and attending various meetings and education sessions in your area?*

I regularly attend various trainings in our kebele. I work as a community volunteer and by virtue of this position I have got many training and attended various meetings. The training I have received include: family planning, environmental sanitation, personal hygiene, communicable disease control , eradication of harmful traditional practices etc… After I received these training, I move around my neighbours and transmit information on the issue and how to use them properly. On top of doing what I have mentioned above I also closely work with the health extension workers. I also use contraceptive service from this health post thus I have close contact. They teach as about health extension packages including family planning.

*What is your lived experience about the health status of your and your children, what do you do when ever any family member gets sick, where do you go or take to?*

Concerning my health status, I feel peaceful now*. What do you mean that you feel peace in terms of your health?*  When I say I feel peace, I mean I have no thing disturbing my wellness. I have no sign and symptoms of illness. I do all my life activities very well. I eat very well. I feed three times a day at least. I have milk in my house. I get meat occasionally as I have to buy it from market but I have eggs at my home as I herd chicken. In general, I am doing fine with my health status. The health status of my children is also wonderful. All of them have completed their vaccination recommended to their child hood age. For their completion all of them have received a certificate from the health post and appraised for that. During their under-five age growth monitoring follow up, all of them have passed through the program and none of them had been under nourished. *Where do you take if in case any of your family members get sick?* Our first contact health institution be it for preventive or curative health services is the health post in our kebele. Once we arrived there, they offer us services we have to receive as long as they could do or referee as to the next hierarchy health institution, the health center*.*

*What is your lived experience related to decision-making at household level?*

I have no problem encountered so far. We discuss all our family matter and decide consensually. One of us could initiates idea and then we thoroughly discuss and reach on decision. Almost all our decisions are in such condition. I may come up with one excellent idea that could greatly promote our family; I and my husband share it and finally act. However, the precedence for decision making is given to my husband. He is the one to take the major share about deciding where our children school should be, what expense is needed for them so on. *What if your husband disagrees on your important issue?* *What would you do?* I have to establish mechanism to convince him through various means. I tell him the benefits of the issue and persuade with him. Moreover, we have established community insurance in our catchment and gather money. Whenever any members of the community get sick, we easily take the person to health institution. This is also how our decisional capacity is growing.

*What is your lived experience in relation to contraceptive use, the time when you started using it, what initiated your to use it and what change you have experienced since you have started using?*

It is four years since I have started using contraceptive service. The reason why I have started using contraceptive is to enhance the livelihood/ free from burdens f/or my children born earlier. For I know that if I continue bearing more children, the likelihood for the survival of both the older and younger children could be highly compromised. *How your contraceptive service use make the livelihood of your children better?* If I bear more than the number of children I have currently means, I cannot provide batter care for my children. We have already limited resource which we can apportion for them. The more children we have the more our economy become over stretched and thread. I cannot buy adequate food, cloth including shoes and school inputs. Hence contraceptive use has helped me to postpone subsequent unplanned pregnancy and share my income for my children in proper manner. Moreover, I have been able to send my children to attend better school in town. I heard about contraceptive service for the first time from the health post (health extension worker). The method formerly I had used was injectable contraceptive and now switched to oral contraceptive. The reason I shifted is based on the information I obtained as there is shortage of injectable method. They said that they have problems in obtaining it. There are also some vague rumors related to the new method (implannon). As a result we requested the health extension workers to give us adequate information about the various contraceptive methods and what the government desire. *What other methods of contraception do you know than those you have already used?* On top of what I have used I know methods such as surgical implants, loop and condom. *What is your lived experience in terms of your general livelihood related to contraceptive use?* Overall my life situation is improving. I have no stress about uncertainty related to what to feed my children or what to give for them for their school. I am free to do whatever activities I plan without being challenged by the problems of pregnancy or thought of caring for young child. I live descent and relatively cool life. *What do you mean that you live a descent life? How your life now is different from your non-contraceptive use time life?*

When I say a descent live, I am not only measures this in terms of having what to eat but for me getting rest as I am not pregnant now is very important. I say ‘efoy’, taking a deep breath and thanking the lord for the comfort I have now. I say now ‘efoy’ comparing to my previous non-contraceptive use time where I forced to bear children in close manner. During that time I had no enough time to care for them and hardly get time to rest. Now through contraceptive use I got relief from that for the last four years and living in ‘efoyta’, peace and rest. Our lives before contraceptive use were full of burdens with young children and looking for what to offer for them. It had made our lives stressful (both I and my husband), hectic and no ‘efoyta’.

What do you feel now as you are not pregnant this time? I feel very happy. I am greatly encouraged as I am caring for my older children properly. I feel happy as our children are attending their school and we think they will have bright future. This is due to the benefits of contraceptive service that I am not pregnant now. How do you feel about your health status since contraceptive use? I am okay and feel well and healthy. When I compare with my pre-contraceptive use, this time I am healthier than that time.

Contraceptive use has created wonderful condition for us in terms of improving our income and properly manages our resources. We plan what and how to expend our income for our domestic and children’s school expenses. As I said above, through contraceptive use, we reached on planning the number of our children and created a faire balance to our income. *What is your experience about the overall contraceptive utilization?*  For me contraceptive service is wonderful remedy that has improved my overall status. I, therefor, not only taking the service for myself but also informing and educating my fellow women to take the services and enjoy the multifaceted benefits of it.

**Key for colours:**

*Light blue= before contraceptive use*

*Red= after contraceptive use*

*Green=type of contraceptive method used*

*Purple= reason for method switch*

Individual In-depth Interview # 1

Chama hembecho

Age: 32

Education: 8

# of children: 7

Year of contraceptive service: 6

*What is your lived experience in terms of contraceptive use related life circumstances, livelihood and over all patterns*?

In the past I was ignorant about contraceptive services, what it was mean and what benefit did it have despite I was educated to grade 8 in formal education. Life before contraceptive use was occupied by frequent pregnancies and child births. I almost bear yearly basis. After I started using contraceptive method, I saw changes. *What you mean by changes and what changes you saw?*  When I say a change, as I said earlier before contraceptive use I gave births year by year and all my time was occupied by pregnancies and child care. Contraceptive service use has created for me to get gap and prolong pregnancy interval. The health education I got from health extension workers about contraceptive service has also created conducive environment to share ideas about our reproductive life between my husband and I. The former unregulated fertility has hampered our development. We are now aware and convinced about child spacing and family planning. This enables us how to plan our life and the number of children we will have in connection to our economic status. We are now aware that if we will have many children without plan, we could not properly care for them including their education, feeding etc…. We decided to regulate number of children we will have for the reason that we have limited economic capacity. In this regard, we have benefited a lot. The other dimension I would like to mention is the change I saw in respect of my children health and nutritional status. Before contraceptive use, I had very frequent births one after the other in yearly base. It was meant that before the first one get adequate breast feeding, the subsequent pregnancy was in my womb. For fear of the harm for the subsequent pregnancy, I forced to stop breast feeding the earlier child. Therefore, that child gets harmed nutritionally and lack of other care. He/she became emaciated, un attractive and frequently attacked by illness (እጅ እግራቸው ቀጫጫ ይሆናል). My experience after contraceptive use in this regard is that I am able to space the interval between pregnancies to at least for five years. This means that a child born before five year grow well, handled properly, got breast feeding adequately. He easily catches up his/her normal growth pattern like any normal neighbour children.

I first used norplant and remained with it for three years. Then after three years, I switched to injectable (depo-provera). I am now on depo-provera and altogether I have been on contraceptive methods for the last five and half years. *Why you wanted to switch from implant to depo-prover?* I was not comfortable with the implants. When I say this, as I am a rural woman required to do many household activities, implant was seen to be discomforting me while doing all the activities. Sometimes I felt weak, developed a burning sensation in my stomach, have no milk to drink or gruel to take in. I thought that implant is the one which caused such feelings and I also experienced dizziness and head ache. For the aforementioned problems I have consulted health extension workers and they put me on some medication. After that I decided to see the alternative contraceptive method, the depo. *Are you now comfortable with depo-provera and the problems you faced earlier had gone?*  Since I switched to depo, I haven’t noticed any problem. When I was on implant, I had heavy menstrual bleeding as result, I feel head ache and dizziness on the way to market or anywhere I move.

*What has initiated you to use contraceptive method? How was your experience in reaching decision on this matter?*

I was not aware about contraceptive use and related benefits before the health extension program. Health services were not accessible in our localities before this program and we were ignorant about health services such as contraceptive use in vaccinations. When the health extension program has been established in our kebele, the worker started to inform us about various health extension packages including contraceptive service use and related benefits. They showed us the difference between unplanned and planned fertility and mechanisms how to control our fertility. After receiving the health education about family planning from the health extension workers, I shared the information with my husband. He also agreed and allowed me to take the contraceptive service in order to regulate our fertility and postpone pregnancy to the time when we desire it.

*What other benefits of contraceptive use did you experience while you use it in terms of your children status, your livelihood, physical mobility and other life arena?*

It has tremendously benefited us. One thing with my child health aspect is that I used to exclusively breast feed my child after I started using contraceptive service. The reason is that it helped me to avoid another pregnancy and given me chance to confidentially breast feed my child. I also got time to feed my child well after six months on complementary feeding. I prepare for him food from various mixes. Over all, contraceptive use has enabled me to better care for my children. It has eased my life and improved my status in community. When I born children year by year, people in the community have been pinpointing their figure on me by saying that the wife of Mr. X is getting pregnant yearly and intimidate me, look down on me. Contraceptive use has created opportunity for me to take better care myself, have good cleanliness, increased awareness of lives. It has also improved the relation with my husband. I have no fear of pregnancy; therefore, enjoy sexual relation with my husband. He is happy about that as formerly I was reserved and unhappy whenever he request for sex. Contraceptive use has also created better chance for me to involve in income generating activities. For one thing when I was pregnant, I hardly help my husband in out of door activities and waiting his hands. He used to purchase something when I was in puerperal period. The frequent delivery and care during this time had drained our fragile economy. But now, through the use of this method, my husband has got some relief at least.

Moreover, this time we are able to plan our income and expense. When I was bearing children every year, we were not in position to save money and forced to sale whatever we have such as cattle or any crop we have. We sold them even during cheap season as we were not able to wait until we would get better price. Now, the condition is different. We only expend for planned issue. We wait to sell coffee or other crops until we would get better price. We also now invest on our children’s’ education, health, buying clothes and school uniforms etc….

*What is your lived experience in terms of education in your family, yourself and your children? How you related contraceptive use to educational experience in your family?*

All my seven children are in school now. The last one is in kinder garten and the rest six are in primary and secondary schools. My first and second children are females. The older one is grade 11 and her immediate younger one is grade 10. I attended school to the level of grade eight and stopped schooling. *What has initiated to send your daughters to school?*  In our tradition in the past leave alone to send females to school, they were deprived of many social and human privileges. They were never considered full human. However through time females have witnessed that they are competent in every sphere of life. I have observed that females working in many public and private organizations such as teaching, health institutions, finance organizations. Now a day every organization requires them to function. When I saw these all, I am motivated to send my daughters to school as their future will depend on what they obtain today. I am cognizant that they will help me if they get educated. I do so for my daughters with great commitment and regretting for my inability to complete education. I say for myself that if I were educated, I would have obtained better position in society and earn better. So, all these have energized me to send my daughters to school. Contraceptive method use has enabled me to send my children to school this way. I got time to care for them, prepare better food, keep their clothes clean, and support them in many ways such as paying for their uniform, other expense. If I was not using the method, it is clear that I could not get enough resources to support them. They may easily drop out their schooling unless I support them to do so. Since I am not pregnant or want to be pregnant in future, I have great ambition to see my children climbing their high up educational career.

Moreover contraceptive method has enabled to involve in income generating activities. I go to local market and buy butter and coffee, gather them and take to larger market at the woreda town and sell there. By doing so, I generate some profit out of these. Contraceptive method helped me so by averting unwanted pregnancy. It is clear that if I am not on the method, I will easily get pregnant and cannot move from market to market as I do now. I therefore, generate my own income. *What is your lived experience in mobilising resources at your family to expend for various reasons such as school fee, purchasing clothes, etc…?* Most decisions in our family are passed jointly between me and my husband. We thoroughly discuss matters arising in our family and reach in agreement. Contraceptive use contribution for this joint decision is explained in terms of our close collaboration and listen each other. We plan for the future of our children to further push them to higher level. We have dreams to educate our children to the master degree level. For this contraceptive service use has created nice chance to share ideas between me and my husband by averting burdens of pregnancies and child births.

*What is your lived experience in terms of your health and that of your children in the era of contraceptive use?* I am well since the time I started using contraceptive services. When I got pregnant, I had severe morning sickness but thanks to the method I have no such problem now. I have no medically notified disease or health problem. My husband is also healthy. With regard to my children, none of them are sick now. They properly attend their schooling, help in some domestic work whenever they have spare time and then study their subjects. *Where would you take if any one of your family members gets sick?* I use health services such as vaccination and family planning from the health post. Any health problems above the level of health posts, I will take to either Hembecho/Areka health center or Dubbo hospital.

*What is your experience about women in your neighbour who are not currently using contraceptive services?* To my knowledge except those old age women and widowed ones, all reproductive age group women eligible for this service are using now. The health extension workers in collaboration with community volunteers provide continuous health information and women turn to use the service.

*What is your lived experience related to your involvement in leadership and political environment since you have started using contraceptive method?* I work as a leader for thirty households women in our surrounding. I inform them about reproductive health matters, child health, saving and other issues. Contraceptive service use has given opportunity for me to spare time for such issues. Had I been pregnant or had young children, I would never attain such position in community. Likewise I share information related to general environmental sanitation, personal hygiene, vaccination, exclusive breast feeding and complementary feeding. There are other women like me also do the same thing in their community.

*How do you see the situation in past time, the time of our parents, before the commencement of health extension program and the health extension period in terms of contraceptive services and reproductive health outcomes?*

Our parents lived in dark without seeing changes. That time there were huge infant and child deaths. Lives were miserable. The women in the past lived being pregnant and bearing children. They lived in poverty life were they hadn’t had anything to eat or give for their children. Children were malnourished and sick. This time people are aware of their health issue, they know what to do and where to go whenever they need health services. The health extension workers are doing great thing to the community in accessing health information and services.

*What is your opinion about the contraceptive services sustainability and anything you need to suggest for future?*

One point I want to forward is about the fertility desire of our society. In the rural part of our area people still desire for more children. In urban area as we hear people need less number of children. Therefore, I wish also the rural women have to understand problems related to unplanned and high fertility. I also feel uncomfortable with having seven children. If I had known contraceptive methods earlier, I would only stop at four children level. When one has many children, it is difficult to handle all them properly. Life this time is not like the same as the past. Resources are becoming scarce and life becoming expensive. Therefore, couples should be aware of the situation. If I haven’t started contraceptive use I would have three more children. Contraceptive use has prevented me from having three unwanted births. This is not only created good opportunity to my family but also to the community in general. If all women avoid such unwanted births, this means helping the community and the nation grow and develop. It helps to reduce poverty and improve livelihood. Now most of our community members think their children better grow and develop. They want to see the bright future of their children through education. We all dream our children to be professional: teachers, clinicians, managers, etc… I also help those women who are not currently using contraceptive method to use the service. I will share my life experience and show them the benefits I obtained from contraceptive use.

District Boloso Sorie

Kebele: Chama hembecho

III #2.

Age: 36

# of children: 7 ( 5 males and 2 females)

Education: 6

Year of contraceptive service: 6

*What is your lived experience in terms of contraceptive use related life circumstances, livelihood and over all patterns*?

I got married in 1982 E. C. After one year of my marriage, I gave birth to my first son. While I was breast feeding my first child, in less than a year time I got pregnant gain. Imagine, I could not give breast milk for my first child adequately and properly. Not more than a year for my second child, I gave birth to my third baby girl. I had difficulty to recognise who is who, as they were born in very close gaps. During that time there was no contraceptive services. I was harmed as I was young so as my husband. By that time , despite having goop size of land to be cultivated, we had no oxen to plough or cows to get milk. Both of us run to market to get basic needs. When I went to market, I left my children alone in house. I couldn’t give them my breast milk as I pass significant time outside my house. Almost all my children were badly affected by life challenges. They couldn’t get enough to eat and drink.. In such situation, I got pregnant for the fourth time. Until I had four children, I have never used contraceptive service. After this time, one health worker by his name called Addisu Sadebo, approached me and informed me to take contraceptive services in order to space pregnancies. The information reached me after having five children. After that I discussed the issue with my husband and started using contraceptive method. Had I known the availability of this service I would have never be harmed like that. I took contraceptive service for three years and then gave birth to my sixth child. The recent child is stronger and well appeared as compared to his older children. After I started using contraceptive service, I have constructed better house. I was in very small house before which looked a kitchen. I got many live improvements since I have been on contraceptive services. I sent my children to school. Two of them are now attending their schooling at grade 10. The third one, a girl is grade 8, her younger one is grade five and the other one is grade 2. I was really poor before starting this service but now I have been able to eat well, dress well, owes animals like cows, oxen which we hadn’t before. Both I and my husband become able to engage in various income gaining activities. On top of his agricultural duties, he does carpentry work and earns more income. I, in my part involved in local trading and generate some income. With these resources, we comfortably care for our children both at house and at school. I wish I had this service long back before much harms had happened me. I still regret that what if I had this service some times in past by the time when I was with full energy. I gave seven births before I started to use the service. I only gave one birth after the service.

I give all births in the hospital for fear that I had problem with labour. I guess my uterus is weak and couldn’t work properly in expelling the fetus during labour. I had serious back ache that interferes with down pushing during labour. In my part I want to stop child bearing with the assumption that I have enough children. I went to hospital with my husband to tubal ligation but my husband was not in agreement. He says, he need more children and still says that” let my wife bear”. Most of the time during pregnancy, I feel discomfort (ማህጸኔ ተንገጫግጮ የለቀቀ ይመስላል) to the extent that my uterus has displaced or expelling the conceptus material. I said to my husband that not to block me from going for the permanent method. I know that you can marry to other woman if I die but leave me for my children to be alive and care for them. I talked to the kebele chairman in this regard to mediate between us.

I am now on loop. The reason for this is that I was sceptic as one day if the injectable or the implant wears off from the stock, what could happen. As I know I have major trouble with pregnancy and as I get older, the problem would be more serious. Thus, I told this to the health extension workers in the health post and they referred me to the health center where I have received the loop. Now I have no fear of pregnancy for 12 years. It is about a year since I am on the loop. I have no problem in my health status. All my children are also well now.

Moreover, now I transmit the benefits of contraceptive services to my neighbours, who are not currently using the services. I explain to them by showing my experiences. I told them that how life was miserable for me before contraceptive use. I specifically stated that the time when I hadn’t had to wear single bedspread after giving birth and stayed necked in my room. So I say to them that come on please and use the services and get rid of the problems and burdens of unwanted pregnancies and child births.

My future plan is how to up bring my children in good ways, how to make them competent in their education and attain their desirable career. I don’t want to have more children.

*What has initiated you to send your children to school/children education? Why are you motivated to do so?*

The main reason I have been motivated to send my children is that first and for most I don’t want them to bear my experience. I know that in the future rural land is getting narrower and narrower compared to the population number. I know that they may be troubled with limitation in resources including lack of cash to engage in trade activities. Instead of being trapped in such life challenges, education is a best means for them to escape from poverty. I know that if they are well educated and competent, either the government or somebody will take care of them (the government will prepare their bread (‘ kawoy etta buudenaa ukkes’)). Their livelihood will be better of as long as they are competent. Since I am getting old and weak, I cannot provide them all their life time necessities. Therefore, I have to prepare them to compute for their future. I recognized that education is a vehicle to ensure this dream. *How do you relate your contraceptive use to your children education?* First of all my survival is crucial for them. I can do every support for them if I survive. If I get pregnant and bear children catch bed in post-partum, I cannot help them in their education. I cannot prepare food for them, wash their cloths, and give them some money for school fee.

*What is your experience in going out of your house for various places such as market, social gathering, church etc…?*

I have no very young child who would hamper my movement outside house. The older children after their school take some domestic responsibilities such as housekeeping. Contraceptive service use has enabled me to postpone pregnancy and get more time to spare for outside door activities. My travel to market is also as part of my general freedom to move outside home. I could never go to market if I had similar situation like before contraceptive use (wode wontta baaxia mala gidyakko hanennashin). *Didn’t you involve in trading activity before contraceptive use? If you did so before, what difference you have observed?* Sure, I did go to market occasionally even before contraceptive use. But, unlike this time, it was at the expense of my children comfort. When I used to go to market leaving young children, all of us were badly affected both physically and psychologically. My absence from home had discomforted my young children in lack of proper care. I was terrified what had happened to my children in my absence. But this time I have no as such worry as my children are getting bigger. My older children are caring for their younger ones simultaneously reading their subject. The older ones are taking responsibilities in my absence what to be done until I will back. I have less worry this time comparing to my previous non contraceptive use time staying outside home. I reached to the extent of sharing domestic burden with my children. They help me both in domestic work and sharing their advice so that comfort me psychologically. I do care for them by availing their necessities.

*What is your lived experience in relation to your husband since you have been on contraceptive service? What about the pattern of relation, establishing commonness, ability to share idea, listen to each other and act as support to each other?*

Yes, contraceptive service use has improved the interaction between me and my husband. It enabled us to have more time that is spared for economic activities. Both of us have got chance to generate income in order to fulfill our needs. Instead of working for other neighbours, we are able to work in our land and produce more. We are not looking others hands, lending money from others, and begging. It has improved our confidence to handle our family matters at our disposal and reduced dependency on others. Instead of looking his hands, I became a partner for him in generating income and support him. These all positive achievements have improved the relationship between me and my husband.

*What is your lived experience with regards to your children’s’ health status since the time you have been on contraceptive service?*

Well, I have touched this issue above but to be more specific, they all are doing fine in the grace of my God. Since the establishment of the health post here in our kebele, they have no problem. Even I do not remember whether they had big head ache. I have gained better knowledge how to handle my children since the time of this service. The health post has helped us in providing bed nets which prevent mosquito bites. We also get educated how to improve our personal hygiene and sanitation. These efforts have greatly decreased the likelihood of our children being attacked by diseases. Children born after contraceptive use have got better care than their older brothers and sister. The older children had frequently affected in many diseases. I took them to health institutions many times. The two children born after contraceptive use are healthier and stronger. They had never been affected any noticeable diseases. They have received recommended vaccinations and received certificate for their accomplishment from the health post.

*What is your experience relate d to decision-making at household level? Who is the primary decision –maker at household level? How do you relate your decision-making experience toward contraceptive use?*

We plan jointly for our house affairs including expenses for our children education and other domestic expenses. In his absence I am the one to carry it out. For example, my husband is not with us now. He went away for outside work. Until he comes back, I am the one to do all matters for our family. I decide how to mobilise resource for various household affairs. If we have enough to mobilize from our house, I do so and in case if have shortage, I look for other options. I present all the processes when he come back home. I tell him all the details of expenses including student’s house rent, procurement of school inputs, food expenses etc…, he then reimburse for me. *What is your experience in order to move outside your house without informing your husband?* No! I go nowhere without his knowledge or prior permission. If he says no to go anywhere, I obey him and drop it out. Ones in the past, I used to go to market for trading purpose without his knowledge, and he said no to do so. Finally, I left it and reaming at home. *What is your experience towards your health status lived in contraceptive service?*

By the time when I used injectable method, I had massive non-stop bleeding. I had hided this from everyone except the health extension workers. When I told the problem, they changed method. During that time I was seriously emaciated and people told me to stop the method use. But, I persisted as I knew the problems I would be exposed. The health extension workers put me on the implannon after my request and seen the problem I had. Implannon was nice for me and the bleeding problem worn out after I have stopped injectable. While I was on implanno, I heard about IUCD. As I understood that IUCD has longer capacity to prevent pregnancy, I wanted to shift to it. At the early weeks of the IUCD, I have seen bleeding again. Then I went to the health extension workers and informed them the case. They told me that the bleeding will stop steadily and not to worry. As they informed, eventually the bleeding has normalized and I became well.

*What is your experience in attending various meetings and education sessions?* I have participated in water and sanitation meeting at woreda level. I also closely work in the community gatherings for 30 women. I have no experience of having training in reproductive health. *What is your relation with the health extension workers? How often you visit the health post?* I have smooth relation with them. They teach us about all the health extension packaged. These people are exceptionally strong. They let us know about how to handle our personal hygiene, keep our environmental sanitation, handle our children etc… I convey their message properly. I have constructed latrine and use it properly by fulfilling the superstructure and hand washing facility. People see mine to get experience for themselves.

*Why you stopped your education that time?* My father was not courageous enough in letting us continue our education and I was abducted by. Thus I was forced to stop my education at grade six. If I was not forced to marry, I never stop it. I have still big desire to continue but my huge family responsibilities could not allow me*. If contraceptive service was available by the time you married, what difference did you anticipated?* I tell you frankly that I would stop child birth at three (2 males and 1female). I never go up to 7 children. *So this means that you have at least three unwanted births. Is that? Yes. If you have these much extra births, how do you rate this at kebele and above levels?* I believe this affects family capacity and even the community and the country at large. Let me put this issue like this. I have five male children. If I want to share part of my land when they become adult, no more than a table size land will remain for me. This is at an individual level and if your put together at community and national level this way, you can see how much stressful it is.

*What is your experience in relation to men involvement in contraceptive services use or support?*

They hardly concerned about it. Men in our community don’t seriously take child birth regulation. They think this way. If the older wife gets overwhelmed with children, he wants to marry to new wife in order to get better care. Except few educated men who plan their family affairs, the majority move towards their personal comfort.

*What do you suggest to improve the situation in the future from the government and the community?*

In my opinion the woman has to be smart in handling her husband. She has to be attractive and inceptive for her husband not to push him out. If she becomes clever to tightly handle her husband, she can reduce the chance for her husband to see outside. She has to identify the likes and dislikes of her husband. If her husband continues in contrary despite all her humble and genuine efforts, she has to present the matter to the legal institutions to give decision. *Does your husband have another wife?* Yes, he does. She lives apart from me. He has land near his mother and put her there. He attempted polygamous marriage long back but I accused him and let him dropped it for the first time. But this time he got chance to settle her in his mother’s house after the death of his mother.
