# Supplementary material for: Women's perception about contraceptive use benefits towards empowerment: A phenomenological study in Southern Ethiopia
Source: PLoS One. 2018 Sep 13;13(9):e0203432. doi: 10.1371/journal.pone.0203432 (PMC6136733; doi:10.1371/journal.pone.0203432)
Supplement: S3 File — (DOCX) [file pone.0203432.s003.docx]

**Transcription of FGD, Sidama Zone**

1. **FGD with mothers in Waicho Kebele**

Profile of FGD participants

| S.no. | Age | Education | # of children | Year of contraceptive use |
| --- | --- | --- | --- | --- |
| 1 | 35 | 9 | 4 | 5 |
| 2 | 25 | 10 | 4 | 10 |
| 3 | 30 | 5 | 8 | 10 |
| 4 | 32 | 1 | 03 | 7 |
| 5 | 30 | 6 | 05 | 12 |
| 6 | 25 | 4 | 4 | 6 |
| 7 | 35 | 7 | 7 | 10 |
| 8 | 25 | - | 5 | 3 |
| 9 | 27 | 10+3 | 2 | 6 |
| 10 | 32 | 2 | 7 | 8 |
| 11 | 30 | 9 | 7 | 6 |
| **Mean** | **29.64** |  | **5.1** | **7.54** |

Moderater: Abraham Alano (researcher) and Yamrot Haile (assistant)

Venue: Waicho Health post

Discussion started at 10:50 AM, ended 12:00 AM

Qest. 1. *What is it like to live through contraceptive use towards your status with the respect of your income status?*

- - - *What is your experience about economic status as to fulfil your basic need, your children and the family as a whole?*
    - *What is your lived experience like as far as you started to use contraceptives?*
    - *How you experience the situation in improving your income level at your individual and family level?*
    - *Learn the experiences as expressed by the women using as many probes as possible until information get saturated.*

Discussant # 1. We were unaware of ourselves before we use contraceptive methods. We had no time to clean ourselves. We have no time to think for ourselves. We were untidy; our body smells foully, children urine was all over our rags and bodies. We start ramifying in the morning to prepare breakfast, and then engage into our daily routines such as preparing lunch, fetching water, gathering fodder for cattle, cleaning cattle waste etc. Our day goes as we were swinging here and there. But as of the time we started using contraceptive methods, there have been lots of changes: we got time to give breast milk to our new born children, we got more time to give better care of our children, we clean them properly, clean ourselves. Now, we are doing very well. No one says us now unclean or foully smelling like a mother in her early periperium (አራስ አራስ ይሸታሉ የሚለን አሁን የለም). Now we are like anybody else in the community. No one can blame us for uncleanness. We have now pleasant smell/odour like any one (ጥሩ መአዛ እየሸተትን ነው የምንሄደው). Our life is good as of the time we started using the services. We feel we are doing well and comfortable.

- - - *How you experience the situation in improving your income level at your individual and family level?*

Dis #2. Before this service use our lives were full of fear and challenge. You know we were thinking of subsequent pregnancy immediately after birth. We wished to abandon it but it was not possible as long as we live with our husbands/men. Since we started using the services, we got big relief in our lives. We got peace in our mind. We have peace all the way we move. When we see things in comparison (the time before contraceptive use), we were tight in all our lives. We carry new pregnancy in our wombs and breast feed the elder young children. Thus, we bitterly disappoint God for our unfortunate lives (እግዚአብሄርን እናማርር ነበር). Now we are free from such life challenges. Formerly we had many problems including deep rooted poverty. Moreover, we experienced un spaced/frequent pregnancies and child births which further aggravate the situations. We worried about the situations. As a result, we had no peaceful relations with our husbands. Our lives were turbulent. Now things are different. We use contraceptive services and feel free to do whatever we want. For example, some of us have used contraceptive methods to about ten years. You can understand that how much we have gone in this regard. This is not an easy issue. We got big relief indeed. We are free from worries now. We feel comfortable now. We manage our children with small amount of income today. We are able to send them to school. We keep their hygiene and provide them with basic needs. We lead our lives smoothly and thankful to our Lord. We are now at rest.

Diss #3. As the previous discussants stated it, before commencing use of contraceptive service I had experienced child births within ten months of the previous child birth. Without properly providing breast milk for the older child, I bore the subsequent one. Getting adequate lesson from that circumstance, I have started to use contraceptive service. I spaced for 10 years to give birth again. I got peace and comfort in my life since I started using contraceptive service. I continued my education from where I have stopped. I married when I was grade nine and gave births for three children. After using contraceptive method, I restarted my education from grade eight and completed secondary education. I have also completed college diploma now. Now, we are the living witness for others about the benefits of contraceptive use. Many women are motivating by getting experience from us. A lot of married women who dropped their schooling before have started again. Really we are thankful to both our Lord and our government. Our lives have been transformed from previous untidy/dirty situation to clean ones. The previous life was also challenging not only for us women, but our counterpart men as well (እንከዋን እና ወንዶች ቆሽሸዋል). The reason for that is having many children. The former lives associated with unplanned, too frequent and too many pregnancies exposed us to massive bleeding. Contraceptive use also saved us from such complications by spacing pregnancies. Our beings and lives have been stabilized in the grace of our Lord. Priase be to the name of our Lord, our lives that of our children and husbands became joyful. We got freedom and rest.

- *You have mentioned that contraceptive use has contributed to the improvement of your income. Can you elaborate this how your income level due to contraceptive use has improved?*

Diss #4. Before I have started using contraceptive service, I gave three consecutive and too close births. I was attempting to evacuate the third pregnancy as I used to carry the two older young children at my right and left arms. I carried heavy load to abort that pregnancy (heavy root and bark of inset). After use of contraceptive method I spaced pregnancy to six to seven years. This has given me opportunities to involve in cultivating farm land and produce crops and other agricultural products. By doing so I improved my income and mobilize this for my children use. Therefore, this method/service has greatly benefited me.

Diss #5. Before contraceptive use I got pregnant frequently. Pregnancy took place without my will or awareness. As the gap between two pregnancies was very close, I was puzzled whom to give my breast milk, for the one who born late or for the early one. I was really worried and challenged. Through such ways, I took care of my older children. When I heard about contraceptive use, without telling for anybody I started to use it. My youngest child now became big. Contraceptive service use has given me time to properly handle my children, cultivate my land and produce and generate income by my capacity. Now I raise my hands to my Lord and offer him thanks. My life through contraceptive use became harmonized.

Diss#6. For what you asked us about what difference we have observed /experiences since the time of contraceptive use; I say we have got peace. Before contraceptive service use, our lives were messy. We were home for dirt. We smell bad odor. Because we did not get time to wash both ourselves and our children. They defecate and urinate on us. Since we don’t have time and money to keep our cleanliness, we were harbouring fouls. Besides, we had no enough time to cook for our children. Our experience was to be pregnant one year and to give birth other year. We were busy with such issue (pregnancies and child care). We were isolated from relatives, social affairs and no time even to breathe in well. Recently due to the effort of the government and the will of our Lord we have access to either space pregnancy or totally stop it. This situation (contraceptive service) has created peace and stability in our lives and given us freedom of lives. We live in smooth relationship with our partner at our homes. Before contraceptive use as we were busy with pregnancy and child care, we were unhappy to share beds with our husbands. As a result most nights were quarrelsome. The man leaves house in the morning in desperate attitude as he was not properly treated in bed the previous night. He also refuses to eat lunch which was prepared with many challenges.

Now everything is peace. We don’t have such conflict in bed. We enjoy both. He well come whenever he has a desire for sex. I go to market, attend social gatherings, and move freely to where I plan to go. It is all after we have started to take the contraceptive methods. We really got peace. We go to church and worship our Lord. We are free to participate in trading and raise our income and boost our household economy. All other participants supported these ideas exclaiming.

Ques #2. *What is your lived experience related to your educational status or your family members including your children?*

- - - What is your experience related to your schooling situation, your children, particularly your daughters if any?
    - What is the experience with female education related to the contraceptive use? How do you explain the importance of contraceptive use in relation to sending your children to school?
    - If you haven’t reached your educational destiny, what do you explain for us about it as related to the availability of contraceptive methods by then?

Do you think that it could have been different than what you experience now? In what manner? Can you tell us in detail about that?

Diss #4. My livelihood was devastating previously. I worked as a labourer for my neighbour where I used to process local food item from ‘inset’. This work was really tiresome. What surprises is that I did it in a month time after my child birth. You can imagine how much demanding it was. I used to carry my new born where I did the work. I put him near my working place. When I did that I was also thinking of those whom I left at home. I was worried what had happened to them. I was hanged over two poles; to get something for them to eat and leaving them alone at house. Besides these all challenges, my husband could not understand my problems. He wants to have more children. When I wanted to delay pregnancy, he always quarrels with me saying that “let the day that I saw you be cursed”. We had disagreements for long times. Every day and night we were fighting. No peace at all. When he refused to help me, I started to work any activity in order to generate some money. With this now I am able to send my children to school. I knew how much I was puzzled with life challenged as I am not educated. Therefore, I don’t want my children to face the same like me. I want to pay anything to let my children continue their schooling. I never want them to stop despite all the shortcomings I have. I have three children and their grade is 3, 2,1, and beginner.

We women, we have uncountable problem. For example, if a woman expresses her dislike for sex, she cannot. A man forces her saying that she is his wife. He can do what he wants forcefully. The woman is not in position to disobey him. She cannot say no at all. To tell my experience, I went to my mom in 40 days after birth for fear that not to get pregnant again. However, I couldn’t escape that. He nagged me, brought back. He totally failed to understand my problems. He only urged me to satisfy his desire. I go to my mother and he brings me back. He is not ready to change his attitude. On top of these all problems, I am very young and born year by year and really being harmed. He neither listened nor understood my problems. Now I am using contraceptive services and able to tell others to follow my style. I am working outside and earning some money from the work. I use the money to teach my children. Now we are in agreement with my husband as I am not getting pregnant and no worries related to unwanted pregnancy. Praise the name of the Lord.

Diss #9. I born second child after his older child reached grade nine. After that I continued my education and completed diploma before giving birth to my third child. What helped me is a contraceptive method. I could have given six to seven children births hadn’t there been contraceptive service. There are women with such happenings. If I hadn’t used contraceptive method, my education would have remained at grade 8 where I stopped during my marriage. I thank both my Lord and the government who have given us the opportunity. I also became able to be employed at public sector. Generally contraceptive services have benefited most women. It helped us to work and to learn (commence study or continue our education). One share experience from other and continue to use contraceptive method and share the benefit of it. We are being transformed in our livelihoods. Our previous life burden such as carrying a young child at our backs and working heavy work is now got improved. We felt free, comfortable and enjoying lives. Now we breathe deeply. Sometimes we feel worried hearing that some methods are not available at health institutions. Our worries are what could happen next. What if we get out of method stocks? This time is really good for us. We feel as if we are young like girls. We maintain our cleanliness, live free of unwanted pregnancies worry. Even others have positive comments up on us. They say us as we look like a mother with only one child and act as well to do one (ያለ ስጋት እነደሚዳቐ ሆነናል). We now are able to plan our children number and time. Let the name of our Lord be praised.

Disc #2. Now things are different than before. With regard to contraceptive use related experience to female education, we are sending our female children to school. Moreover, we ourselves are continuing our education as we got time to do so. Our mothers brought us in the manner they were brought. They lived all their lives in many challenges and brought up us through those challenges. Now we should not repeat the same up on our daughters. Now we are cognizant to teach our children including our daughters. Their future will depend on what we invest today up on them. They have to be in better position than us. Our mothers did all that as they did not have means to delay unwanted pregnancies. Now we have opportunity to do so and use this to enhance chances for our daughters.

*It is good that you all mentioned that you send your daughters to school equally with sons. But what is your experience about who helps you in domestic work after school?* There is a reality about sharing domestic work. Female children are the one to help their mothers after their school while sons are playing outside. With regard to their feeding habit, still priority is being given to male children. Female child feed the remaining part with her mother.

*Can you tell us about the feeling of society about female education with big emphasis on the attitude of men? Some may say that educated female may be this and that….*

Disc #3. I was grade 5 by the time when I first married. I just dropped it and married. Then I gave birth for my first child. After that I used contraceptive method for four years. Since I have started to use contraceptive method, I have been to involve in many social affairs including church affairs. Before we access contraceptive services we were not only worried about us but to our young male and female children. As now we have contraceptive services, our worries are reduced. My first child is reached to grade seven now. I also teach other children renting house at town. I have determination to up bring them (ከኔ የቀረ በነርሱ እንዳይቀየር ቅናት አለኝ). I want to continue my study from where I dropped if time and situation favours me. I also confirm that contraceptive services have benefited us in many perspectives. We are safe and comfortable at our house and outside. We feel esteemed and tidy proud of our selves.

Dis #11. Everything is good. Contraceptive services have transformed many things. Let me share my experience. My first child is a female. I teach her now. I dropped my education at grade 6 and married. I started my education again and now I am grade 9. My first child has completed her education and now is a public employee. I also teach all my children. My second child is now studying at preparatory class. The third one was completed secondary school and joined TVET program. On my side I use contraceptive methods and share information with my nearby people. By doing so, we are contributing both for ourselves and our neighbours. Our community awareness toward contraceptive service is greatly improved now. With respect to this service women use the services without significant difference between educated and uneducated. The quality of our life is improving. We got time to take better care of both ourselves and our children. As of the time we started to use the service, we reached to a state of planning our pregnancy and child birth. Some of us are able to continue our educations which were dropped at the time of our marriage. We send our children to school and ourselves attempting it. For example, now I am grade 9 and wanted to continue it but I was unable for this year as I was busy with responsibility at community level and all my children are at school. I have a plan to complete secondary school next year. We have well organized contraceptive services at our kebele which enabled most eligible women using the services. We have also a system to share contraceptive services among our neighbours and help each other planning pregnancies and child births.

Our mothers and older women were unable to use the same services. But we are lucky and able to access the services. *Let’s put the use of education and contraceptive use the other way. How did you learn about your first menarche? Did your mothers told you before or you suddenly saw it when it commences?*

All of the participants said no, it happened suddenly. No either mental or physical preparation was made. One discussant stated that she has a responsibility to inform her girl child about her reproductive signs and how to handle it. I have to spare more time to my girl child on how to avoid unwanted pregnancy.

*What are your feeling/comments about the contraceptive service taking into consideration had the service had in place some times in the past (at the time your mothers were in active reproductive age)? What difference could it have brought in both your life and those of your mothers?*

Diss # 8. Both my parents passed away when I was very young child. My mom died when I was two and my dad died at my 3^rd^. I was born at my grandmother’s house where I brought up by her. My grand mom did not send me to school. She sends all her children to school and assign me to do all house works. I grew up in miserable life. The reason was that I grown without my mother. I used to prepare food, fetch water, evacuate cattle wastes etc… My life career had the same and remained outside school. Now I tell my older girl child all what I have faced and inform her how to be strong and take care of herself. I know that no one has informed me about such issue but now I am curious and tell my children.

- - *Let us all share our experience here.*

Diss #3. If our parents had used contraceptive services at their age, they would have better cared for us. As they had great work burden, they considered as just part to share their workload. Consequently, we were unable to progress in our education and end up in early marriage. Now I don’t want my daughter to behave on my way. I want my daughters equally to compete with their brothers. I encourage them to study. I never disturb them from their study. We should never over stretch them with our domestic work. We have to give equal opportunities like their male counter parts. *What is your experience with regard to sustainably supporting girls in their live endeavours including education? Some mothers’ conflicts with their older girls as if they consider them are competing for some resources?* All of the discussants mentioned their agreement in this regard. It is the push factor for girl to engage in untimely marriage by dropping out her school.

Diss # 7. We advise our daughters by giving emphasis on how to be focused on their future career. We warn about the risks of unwise relation with male. They have to articulate on their study at this moment. Other part of life affairs will follow once they attain their educational career. We show our cases as an example. We say that we were victimized because our time was full of challenges. Our parents were not properly cared for us. Therefore, to escape from such back warded life situation they better focus on their career.

*What is your experience related to your health status?*

- *In terms of your overall feeling good?*
- *Your nutritional status, weight, types of diet consumed*
- *Visiting health institutions*
- *Reasons for the visit*
- *Any illness that made inactive, bed ridden, etc…*
- *What is your experience in your health status in relation to your contraceptive use? How do you explain this in terms of improvement or deterioration?*

Dis #7. Now I am using in injectable contraceptive methods protecting pregnancy for three months. Before contraceptive service use, I got pregnant in six month time post-delivery. I bear the second child while breast feeding the first one. The second one comes before the first one walks. I have started using contraceptive service giving births for five children. Thanks to the Lord and the government, now I lead good life. I am free from burdens related to pregnancy and child births. Our sexual life was not conducive previously. It was full of quarrelsome and disagreement. He bits me as I was not willing to have sexual relation with him. Now I am free and have adequate time to enjoy my husband and myself. We meet each other freely and comfortably. *How do you see about sexual relation with your husbands? Do you think is it something minor or less important?* All the participants mentioned that it is not minor or less important. We engaged in martial relation not only bearing children but we loved each other and wanted to enjoy our relations including sexually.

Dis # 8. With regard to my health status, contraceptive method has enabled to plan my pregnancies but I still have problems. My husband only reflects his desire but not caring for me. You see contraceptive use requires better meal and good care. We live in contingent environment. We have such problems at rural here. When we see our urban counterparts using contraceptive methods, they look like orange. Their lives look comfortable. Their body is well built.

Diss # 10. For the question regarding the peace at our house I want to tell what we mostly sense. Now mostly we feel bright and happy. Our houses are full of loughs. If I say no sex last night, everything in the house get messed up. He never gives me money to go to market. All my children pass the day without food. You can imagine how much discomfort the whole family members bear. Some women may have many children and when they get into conflict with their husbands (refuse sex at that night), all their children may be punished in hunger. The man can eat everywhere. He does not want to understand the reason why the woman refuses sex. She might be hungry or tired due to work load. Household decision should be reached on consensus but most men are not agreeing on this issue. They give more attention to their demand and not taking others’ need in to consideration. As a result other family members denied privileges in the house. Since the time of the contraceptive service became accessible, I can say both the husband and wife acts similarly. Both husband and wife are currently able to discuss their matter and reach on understanding each other. Most of the time now we are in agreement and the disagreements are getting less and less. Now he gives enough money for me to serve our children. The reason is his demand is fulfilled.

Dis# 2. My health status is getting better. Peace, eating and drinking well improve quality of life and enhances sexual desire. Naturally females have high sexual plateau as compared to male. But this was not verified at our level in the past. When you eat well and lead your live peacefully, it is normal to have desire for sex. When human being be it male or female, eat and drink, the body get active and hot, thus initiate sexual desire. But with regards to women sexual desire, it is mostly dormant as she is usually overburdened. It was said that the female sexual desire is more than that of the male. This ideas goes in line with saying by the prominent Sidama woman leader “ Fura” “don’t accept or say ‘yes’ for the newly processed inset and requests of male” (አሁን የተፋቀ አምቾና ወንድ የሚልሽን ሁሉ እሺ አትበይ). Fura also added that if your husband asks you whether the space in bed last night had been adequate:- say him” not at all and try to maximize your gain” was to indicate that women have upper hand in sexual power and stamina. Despite the natural reality in this sense, women don’t make their feeling so obvious. This is common in rural women where they usually submit their feelings to their husbands and their children.

Some women don’t make their feeling obvious and not discussing their feeling with their partner. But sometimes such women are quite at their house and open at outside. As a result she may end up looking for a man better fits to her desire and may end up acquiring sexually transmitted diseases such as HIV/AIDS (ሴት ቤት ዝም ብላ ውጭ ተንኮል ትሰራለች). The disease then is transmitted to the husband. A failure to properly discuss the matter at couple level ends up in messing the life of the family and exposing them to disease and death. Since a woman is not free to share her feeling to her husband, she is exposed to many health risks. However if there is a good relation and love between a husband and wife, she doesn’t fear him, discuss the matter openly.

Diss #8. I agree with the idea of the former speaker/ discussant. Let me say something about my life before contraceptive use. I was forced to bear children very closely. I did not have enough to give for my children and I did not get proper food during my puerperal time. I was badly affected by chronic nutritional problems and no desire for live including sex. However, my husband always urges me to have sex with him. When he comes to me I move out. I have no desire as my body was emaciated, no good food at that particular day. Though I am un educated, from my personal experience, I agree with my sister who talked earlier that it is sure that the sexual desire of women is higher than men. You see marital relation is not only for housekeeping or food preparation but is also about………… all said yes: it is to please her husband in all matters including sexual relation. Men need women more than food for being women (sexual affairs). The reason is that after eating well he needs sex at night. If this fails to happen or I refuse to meet with him that night, he withholds the money to buy food for two to three days. We have nothing to get from our yard. We purchase food from market for two to three days. I bought small plastic bag flour two days ago. We ate it the previous days and when I refused sex only for a night, he punished us by withholding the money for food. Meanwhile he used to eat from his mother. Then, I recognized the situation as it harmed me and my children. Now I started using contraceptive method and able to avoid unwanted pregnancy and saved time for my husband. Now I made myself ready to properly treat my husband and fulfil his desire. I am okay to meet him all the time he needs except natural situation forces.

Since I have no full capacity to take care of myself and that of my children, I realized that I have to obey him and avoid circumstances that put me and my children go off food for two to three days. Now we respect each other and obey each other. There is no such conflict about sexual relation. We understand each other to the extent that to meet at day or night whenever he requires it. I understood that I have to say okay for his request be it day or night. I have to make myself ready to accept his request. My previous disobedience had badly harmed me. Now thanks to my Lord, I am free of such conflicts and my home is full of peace. These all is due to a contraceptive service. I am good now, neat, healthy act as young and attractive. I am free from the worries of unwanted pregnancy and its related complications. Our house is full of happiness as we are in agreement.

Dis #6. After marriage immediately I gave birth to my first child. Next to that my intention was to continue schooling which I have stopped due to marriage. But I became pregnant unknowingly. I was terrified and wanted to abort the pregnancy. I tried all means to evacuate the pregnancy at my level. I carried heavy loads and walked long distances to fetch water. All the attempts failed to miscarriage the pregnancy. People advised me to go to Yirgalem Hospital and consult health professionals about it. Then, I went to Yirgalem hospital and asked the health professional as I need to abort the pregnancy. But the health professionals informed me that the pregnancy was beyond the limit to do so and better to continue it. However, I was not convinced by their idea and went to Leku health center on my way and the Leku’s health professionals told me the same as what the Yirgalem Hospital professional informed me. All the attempts failed and I was forced to bear the child. Now things are different from that time. Below the Lord, the government has brought contraceptive services to our vicinity and I have started using it. All the challenges I faced became a history. My house is peace. We have better life now. Our love status is really growing and satisfying. There is no fear for unwanted pregnancies and distancing from my husband. Formerly, I remember that I dislike when night comes as refusing the bed relation with my husband. We had many quarrelsome nights. My house is near the church and people sometimes hear our conflict at night. There were occasions I disappeared from my home at night for dislike of the night conflicts. These all were fear of unwanted pregnancy, child birth and burdens related thereafter. Life with contraceptive use in this regard is transformed. I am comfortably treating my husband in all matters including bedding (our bed relation is full of satisfaction and accommodative). We pass days and nights peacefully and always thank our Lord for these days.

I learnt that night relation is a key for family building. As my sister mentioned earlier, when there is conflict at house due to disagreement in bed, a man go out looking for other woman who would satisfy his sexual desire, he may acquire disease that further transmit to his wife. When a woman sees her husband talking with another woman in the neighbour, she creates a hostile environment like Ethio-Ertrea conflict. But she may not understand the reason for the conflict is disagreement in bed. When she says no for sex at night, the man right way thinks that she has disgraced him. As a result he tries to see a new match outside or find for additional marriage (polygamous). Now there are no as such issues. Since we have started to use contraceptive methods, our community, our family and houses became stable and peace. We live with our husbands in peace and love. We recently never heard about having polygamous marriage. From these all I can say that for a man bed issue is very critical. Its absence may lead him to the extent he may kill someone. So, God has really freed us from such dangerous things through contraceptive use. Now our husbands remind us our contraceptive method appointments. Our lives are full of loughs.

Dis #1. The reason why I was keeping distance in bed or showed disinterest to bed relation was not due to real absence of sexual desire but the fear of pregnancy. As all the former discussants mentioned, we had passed many ugly times before the beginning of contraceptive services. Now we are comfortable and enjoy our lives.

*What is your experience related to the health status of your children?*

- *What is your experience in terms of child growth and development? Maintaining proper weight and height,*
- *How you explain their health status in terms of acquiring disease or illness? Visiting health institutions or other remedies*?
- *How these contribute to your health and general wellbeing? More probing…..*

Dis# 3. In Sidama culture when a very young child dies for someone, people say that “a worm for Mr. X or Ms. X died”. How do you feel about this as currently through use of contraceptives? Is there any improvement about the health your children?

Dis# 7. Though I have given birth for seven children, I have properly brought up all them well. Using contraceptive methods enabled me to give proper care for them. Those children born before contraceptive use were in close gaps and had some challenges. The children born after contraceptive use were in spaced ways and had received better care. All of them were vaccinated for respective antigens. They grow well following normal growth pattern. They walked faster as compared to the previous children

Dis # 9. The reason why people said a young children death as “death of a worm” is that children born in unplanned ways and parents were not seriously concerned about. Thus, a mother new that her pregnancy was unplanned as a result the loss may not count much on them. On the other hand a loss of children now is followed by deep sorrow because the pregnancy was planned and wanted.

Dis #2.I gave birth year by year. One of my children (the fifth one) was very sick. He had eruptions on his skin which comes once a year following seasonal change. Some says it “evil eye”. I used to apply topical ointments but did not show any improvement. Some said that it was a devil sight. We are Muslim and took him to the religious traditional healer ‘Kalicha’. The Kalicha then said us to wash frequently using the reagent he gave us. Despite continuous washing there was no improvement except it turned to be white and then macerated. Meanwhile I was pregnant for second time. I cared for the sick child being pregnant. I used to carry the older child on my arm and the fetus on my womb for 9 months. Still that child experience red eyes and irritation. The health post helped the child to get rid of this problem. Except one, all my children were vaccinated.

1. **FGD with mothers in Gane Kebele**

Profile of FGD participants

| S.no. | Age | Education | # of children | Year of contraceptive use |
| --- | --- | --- | --- | --- |
| 1 | 25 | 7 | 5 | 4 |
| 2 | 30 | - | 8 | 7 |
| 3 | 25 | 6 | 3 | 4 |
| 4 | 25 | - | 4 | 3 |
| 5 | 34 | 8 | 4 | 3 |
| 6 | 30 | - | 8 | 2 |
| 7 | 40 | 6 | 8 | 6 |
| 8 | 20 | 10 completed | 1 | 2 |
| 9 | 40 | 4 | 7 | 7 |
| 10 | 25 | 3 | 2 | 4 |
| **Mean** | **29.4** | **4.4** | **5** | **4.2** |

Moderater: Abraham Alano (researcher) and Yamrot Haile (assistant)

Venue: Gane Health post

Discussion started at 10:50 AM

Ques 1. *What is it like to live through contraceptive use towards your status with the respect of your income status?*

- - - *What is your experience about economic status as to fulfil your basic need, your children and the family as a whole?*
    - *What is your lived experience like as far as you started to use contraceptives?*
    - *How you experience the situation in improving your income level at your individual and family level?*
    - *Learn the experiences as expressed by the women using as many probes as possible until information get saturated.*

Dis #1. We have great change in our lives since we have started using contraceptive services. Previously we were ignorant for contraceptive services and experiencing to bear children yearly basis. Our lives were about pregnancies and child births. One comes just after the other in nine-to-ten months’ time. That was the time which we hated ourselves and our children. They were emaciated, not thriving well and not attractive to see. We ourselves were not well-fed, hygienic and undernourished. After we got information about contraceptive service both we and our children are doing well. We care for our children better way than before. Our livelihood in general has improved as compared to the time before contraceptive services.

Dis #2. We are fortunate to have this opportunity. We thanks our Lord, this is really great opportunity. I have been working as a volunteer in this kebele and did many attempts to share information with my fellow neighbours about contraceptive services. It is after big battle now we are converting many women towards contraceptive use. At the initial time me and others like me were tried to share information but they were misinterpreting that we were doing so because of the perdiem paid for us. Now there are many improvements in our societies. Some of them want to shift from short acting to long acting. We closely communicate each other about the process of contraceptive use. Some of us call the health extension workers to know about the appointment dates for long acting methods. It is indeed a big progress to see such attitude that our community members want to shift from temporary methods to permanent ones. I my self was not user for contraceptive services earlier. When I became aware of the benefits of the service, I was not eligible for contraceptive methods available in my vicinity. I was worried for about not using the available services. I continuously was asking if anything I can do. Then the health professionals informed me to get a permanent method. At that time surgical contraception was not given in my area and I was not able to afford for transportation and other expenses, thus stayed not using. Now the service is being given at my area and done tubal ligation after having four children.

Many women in this kebele now have got permanent contraceptive services (tubal ligation). We are making network of information dissemination about contraceptive services. One tells for the other and the flow continues this way. We inform our neighbours telling the benefits we obtained by using contraceptive services. Now we have various contraceptive types ranging that works for a month to 12 years. These include pills, injectables surgical implants and loop.

Now I am a merchant working partly in the market. I have no worries like previous time as there is no young child who needs my frequent visit. I send older children to school and then work whatever I can do. Contraceptive use has enabled most of us to engage in diverse income generating activities. Some of us became owner of better houses, others bought cattle. We were totally dependent on our husbands formerly because our times were occupied in child births and related subsequent care. Now we are in position to handle our revenue that has given freedom of decision to mobilize our resources to expenses at our level. Generally, this time we lives in better situation maintaining our cleanliness, safety and comfort as compared to the previous non-contraceptive use.

Dis #3 . I used contraceptive service following the birth of my first child. I started this before Gane health post begins provision of contraceptive service. First,I took the service from Yirgalem Hospital in 1995 E.C. Then I wanted to have my second child and gave birth for this child in 1997 E.C. After that I am on a long acting contraceptive method that prevents pregnancy for five years. Now I am properly handling my young child including breast feeding. We the residents of Gane Kebele through use of contraceptive methods see that our livelihoods are improving. We cooperate among ourselves to improve our lives. Instead of waiting husbands’ hand, now we engage in various income generating activities. Some of us are involved in small scale trading and others also in growing vegetables at our garden. We do well since we stared using contraceptive services. Many of our worries have detached from us. There are many measurable changes in our lives. We access contraceptive services in our kebele easily. We receive the services on our respective appointment dates without any worries and go back to our house to do our works.

In the grace of our Lord and due to the commitment of the government, now we are freed from challenges related to mistimed and unplanned pregnancies and their related burdens. We are now capable of taking our level actions either to generate or expend income for minor household activities. Therefore, we are saved/freed from looking hands of someone and started to exercise our rights and autonomy. Simultaneously, we learnt how to use our resources in economic way. We relate all these to contraceptive use as it averted unwanted pregnancies and created opportunities to properly use our times. We have no as such worries about cries of young children. Our minds and hearts are cool and restful now.

Dis #4. We were not well initially. Contraceptive service use has created happiness for me and my family members. I gave births for three children before commencing contraceptive method use. Then I was on contraceptive method for five years. And then wanted to have another child and stopped using. I use contraceptive method to space pregnancies. By spacing it, now I have reached to seven children. My first child is 10^th^ grade now. The second one is 7^th^ grade. My children are now turned to help me in many perspectives. After having seven children, I went to hospital and done tubal ligation. I put all my efforts and time to look for my children. We all are doing great since the time I started using contraceptive services.

Dis #5. I was not comfortable with both the injectable and pills initially. As a result I used to discuss the matter with health extension workers frequently. They informed me all the available options for contraception and advised me to choose among the alternatives. They indicated the possibility of using permanent method. Consequently, I went to FGAE Aposto branch clinic and they referred me to Hawassa. I got the service for tubal ligation at Hawassa. Now it is okay. I had heavy bleeding before and my life was full of misery. I have many children; my husband is public employee and mostly lives at town. I and my children were facing many challenges alone. After having done the tubal ligation, I am well. My menstrual cycle is now normal. When I think back being on today, I really amazed in comparing the two conditions (before and after contraceptive services). I feel that I haven’t given birth ever. After I did tubal ligation, I share my experience to my fellow women in my vicinity. By the time when I did tubal ligation, I paid for the service but this time it is free. I tell women to use this service and many of them are planning to use it. Now we receive services by our children here at our kebele. This is a wonderful chance for us. When I decided to be on permanent method (tubal ligation) many people frustrated me as if I would die or handicapped. I knew that I gave many births at my young age. When I shared my experience to the health professionals, they really felt sad and about to cry on how I did at that young age.

After tubal ligation, I act as a role model to many women and teach them telling my life experience. Some people say me that am this woman after tubal ligation doing okay? Look, how she behaves? But, I am okay and say to them label me as what you want. That doesn’t make any influence up on me. I know the benefits I got. I can say that this time is the time our Lord has turned his eyes to us, the poor. I did send all my children to school. Some of them are now employed. They generate income and support me at this time. Now they take care of me. The time has come and they are paying back to me. Others are doing well in their schooling.

You see the time when I bear children year by year, no one visit your. I think things are getting better now. That time it was common for us to engage in domestic work immediately after birth dripping our blood all over the floor (ደማችን እያንጠባጠብን እንሰራ ነበር). This time is good. Women are doing well as compared to the previous time. We are happy and joyful. Our health and physical appearance is better when compared to the time before contraceptive services. As a result the difference between the mother and the daughter become very narrow, they look like each other. We live as if we are sisters. People are challenged who is elder and younger. Now I do my work. I have poultry, garden cultivation where I work most of my times. The freedom has created wonderful opportunities for me to involve in income generating activities and use the income to manage my house properly. I am now considered as a grace for my husband who abandoned me and my children by the time we were in miserable life (ያኔ በልጆች ብዛትና ጉስቁልናዬ ሸሽቶኝ ለሄዴው ባሌ እንከዋን ክብር ሆናኛለሁ).

When I started using the services, the providers were strange (የራሴ ወገን ባለመሆናችው) for me. I was a bit frustrates when they surrounded me. I did consider as if they were to kill me. I said to Lord, did you bring me out from my home to kill me? However, the professionals did nice for me. They gave best care, treated me well. Currently, there are many changes. Most contraceptive services are being given by our children here at the health post. We lough with the health extension workers, enjoy with them and get the services. I am not only sharing my experience to outsiders but to my children as well. One of my daughters got married and I say to her; pleas plan your pregnancy. Use contraceptive services. There are various types out of which you can access the one suits to you. Some people consider me as if I am mad. But, the reality is not that. I am really committed to avoid miserable lives from my community which I passed before.

Dis#6. I have three children. My first one is 10 years old. I work in one local enterprise and wanted to stop by two births. But I missed unknowingly appointment date for injectable contraceptive and got again pregnant. When I wanted to use implannon, people told me many rumors about it. They said that implannon is not good. It may move out of its place and may cause pain, discomfort or move to brain. But I was determined to take it and done. Despite all their rumors, I went to the health post and received the service and doing well since then. I am doing all routine works as like before. I carry water, clean house, move to various places, dig and collect firewood etc… Nothing has gone wrong. Rumors remained rumors for me. Everything is okay with me.

Dis #7. I gave birth to eight out of which four were died. I used the therapeutic feeding center and food supply. During this time a health extension worker advised me to take contraceptive method to space pregnancy. I also realized that the problems I had earlier were related to mistimed pregnancies and child births. Then, I was convinced and put on implannon. After 8^th^ day of the insertion, my five months child was died. Now I left only with three children. I accepted that all the advice was to improve myself. Now I clearly understood and appreciate the advice of the health extension worker. I am working properly and earning. I am not looking the hands of others or queuing for food supplies support. My life shows some improvement. *How do you describe improvement in your livelihood?* When I say life improvement, instead of receiving food support, I started working and earning. Thus, I buy food for my children and fulfill whatever needed for my family.

Dis #10. I paid 270 ETB for contraceptive service (surgical implant) at Yirgalem hospital before the access to contraceptive service improved. I also waited for five days to get service besides paying the money stated above. Now the situation is different. Service accessibility was greatly improved. As the former discussants mentioned, we got service here at our kebele by our children. This is really big shift. We tell all our feelings without keeping it secret to the health extension workers. We don’t have problems of long waiting hours for services, no need to go on daily base to queue up for services, no one say us bring your card from card room. We receive services at one stop shot. My first child is 2^nd^ grade and his younger one (second one) is ready to go to school. I lived with surgical implant for four years and left with one year. I thank the Lord, the government and the service providers as we have services at our locality. This service has given us better way of living. Now we have no worry about daily remembering of taking the service or thinking of the appointment date. Through the improvement in access to contraceptive services, our husbands are also largely convinced and remind us if we are about to miss the appointment. *How is the pattern you use the service? Does your husband know whether you use it? How is his reaction?*

Formerly, I use contraceptive method in secrete. I swallow pills at the night without showing him. When I shifted to the injectable ones, I did the same. I went to the health institution secretly and get the service there. Now that time has passed. I use the service obviously with his knowledge. I am not worried about saying whether he sees or not. He reminds me about the appointment and encourages me to use the services now. We do all things at our home collectively. We discuss issue of contraceptive as well. Both of us involve in income generating activities. There is no as such hiding things like the old time. We talk to each other, share ideas about contraceptive use. Formerly, it was full of conflict. If he suspect that I use contraceptives, he says oho… you did not want to give birth and challenged me. Now we established good agreement, we feed from the same plate, we ask each other what to eat, which type of food to prepare. We are now safe and comfortable. I have been five years since I gave my last birth. Since then I am okay. No complaint about health problems, I move freely, work conveniently. My children are doing well. Everything is okay thanks be to the Lord, nothing to say more.

Que 2. *What is your lived experience related to your educational status or your family members including your children?*

- - - *What is your experience related to your schooling situation, your children, particularly your daughters if any?*
    - *What is the experience with female education related to the contraceptive use? How do you explain the importance of contraceptive use in relation to sending your children to school?*
    - *If you haven’t reached your educational destiny, what do you explain for us about it as related to the availability of contraceptive methods by then?*

*Do you think that it could have been different than what you experience now? In what manner? Can you tell us in detail about that*?

Dis #2. I was hesitant when the first information about contraceptive method came to me through the volunteer community member. I saw difference in lives between those who were on contraceptives and those not. I then started to use as of last year. Now my young child is three years. On top of the likelihood getting freedom to engaging various activities, I have also got peace in my family since I started using contraceptive method. *Is there anything that you feel that missed at your level but which you don’t want your children to do so?*

Dis #4. What I want to say is that my mother gave birth for 8 children as she had no access to contraceptive services. Had she known and had access to the services, she would have never done so. You know there was no chance for a married woman to go back to school formerly. Her destiny was limited only to bearing children. The trend about contraceptive service use earlier both at the urban and rural part was by the confirmation of husband or partner. A client should convince service provider about the issue. The ignorance and lack of access to services forced my mother to bear such huge number of children. Formerly only educated women had better chance of using contraceptive services. For most rural women the reproductive decisions were at the hands of their husbands. If they agree to stop births, women do so or if they desire more, women were forced to bear children.

I now at my age have given births for four children and stopped to bear more by using contraceptive services. I wish my daughter only to bear two children. I have started informing her from this time on ward. I agree that the future generation should not be challenged as we did. I know the challenges I have with four children. It is not easy to take care of four children, to give them adequate food, better cloths etc… Our parents had lived in complex situations tolerating turbulent and oppressive paternal environments.

Dis #1. I cut my schooling from grade seven and then married. My mother was uneducated and didn’t know the benefits of education and let me to cut my education prematurely. I pushed to level of grade seven by personal efforts covering my school expense myself. I was engaged in many income generating activities to continue my schooling but when I reached grade seven, things became out of my control and forced to marry. I send my children to school. Fortunately, my first child is female and she is grade six. Her younger one is grade three and the third one is grade one. Others are young for school but will commence when their age due. *How is the trend of involving in domestic work after schooling? Tell this by comparing female and male children*

Dis #10. Our parents prohibited us from schooling by saying that let the female stay at home and support her mother in domestic works like processing food from inset. I was attended only to grade three. However I send my children to school. My first child is grade two. We follow him after his school looking at his exercise books. His father helps him at night in his study and doing homework. Actually I am not in position to help him academically because I am ignorant or my eyes are blind for education. Instead, I do help my children so that their eyes will be open to modern education. My husband says that if I had pushed my education in the past, I would have done better now. This is why he always feels discomfort (ይጸጸታል). I know that my chance for education has already passed and I have to do well for my children. *Tell us your experience how you care for your female children in schooling and support their study at house.*

Dis # 9. In past all the attentions were given to male children in Sidama culture. Little attention was given to female children. Currently we see both male and female children equally in many perspectives. In terms of education also the gap between male and female children is getting narrow. I encourage my daughter to proceed on her education and never let her to miss her class attendance. I stopped my schooling from grade four. I have great concern and feeling about my education. Taking experience from mine, I encourage my children to be strong on their education. One of my daughters is grade 8 and her younger one is grade 7. I tell them to continue in schooling and never think of marriage at this age. I emphasised to them even not to see other girls who marry cutting their schooling and keep gazing their eyes on their education. I never differentiate between male and female children. I equally treat both in their education. I encourage all of them to study. I prepare coffee and call them to drink and then to go back to their study. I understand the long lived discrepancies between male and female that offer more emphasis to female education.

Dis # 8. I was grade 10 when I got married. I have one child and started using injectable method. Some people say me why do you take injectable only having one child. I say such question comes from ignorance to the issue.

Dis #5. I gave to twin births when I was so young. Immediately after that I got pregnant again as by that time contraceptive service was not accessible like this day. I started using contraceptive service after giving third birth. People influenced me not to take the injectable one and I also stopped for a while where I became pregnant for fourth time. After that I continued using the service again. Now I am okay and my last child has grown well.

*Q # 3. What is your experience related to your health status?*

- *In terms of your overall feeling good?*
- *Your nutritional status, weight, types of diet consumed*
- *Visiting health institutions*
- *Reasons for the visit*
- *Any illness that made inactive, bed ridden, etc…*
- *What is your experience in your health status in relation to your contraceptive use? How do you explain this in terms of improvement or deterioration?*

Dis # 2. It is known that human being eat whatever he/she needs as long as he/she works. If he doesn’t have enough, he eats whatever he gets (ቂጣ ያለመበያ ይበላል). Currently people started saving. In past time our people eat well when they have but not worried for the future. Now we have changing our feeding habit and exercising to eat three times a day. Our working culture is also improving and productivities as well. We also discourage harmful practice such as wasting work time and extravagant expenses. Both I and my husband engaged in income generating activities, and cover our domestic expensed collaboratively. This gives us better chance to fulfill our food expenses. The number of family members was large previously reaching 8 to 9 but now it is decreasing. At that time only one person acts as a bread winner. Now the family size is relatively decreasing. This creates good chance for household to get better and enough food. This time we discuss all matters openly at household level and in the community how economically use our available resources. In the past when the coffee ripens, it goes to market by many routes. Father officially takes to market, mother on her way and children. These have opened means for unwise /extravagant expense and missuses. The discussion forum has helped us to keep our resources and save for risk times. Thus, we use properly and distribute evenly for our basic necessities. Unlike the previous time no one says distant women from economic decision.

*Can you tell us focusing to your health status related to the era of contraceptive use?* I have done tubal ligation and physically doing well. My body weight and appearance is normal but I have no peace at my home. My husband is always nagging me and full of conflict as I did the tubal ligation by my own. I did it because I have eight children and the 9^th^ one was died. I am convinced with this number but my husband is not. Because of the continuous conflict, sometimes I think of suicide myself. He wants to marry another one and I said go on.

Dis #4. My husband was poor when he married me. Through the joint efforts I made him prosperous. He was protestant Christian before but when he got money and bought a car, he denied me and married to other wives in town. He felt that I am not matching him at this stage as he is educated and prosperous. He married to educated and prosperous women. He was from poor family but I made him prosperous leaving out all my privileges and begging my father to give me some money. I worked day and night to make him a full man. I never thought of my personal comfort and safety. I have invested all my time and resources on him but the final return has been denial.

Dis # 6. We have many challenges. We don’t have time to mention all these. Let’s keep it away and concentrate on our topic. All agreed on this directives and turn to the discussion point.

*Was there no benefit of contraceptive service since its inception here? Do the side effects out way its benefits?*  We haven’t faced major problems. We know that its benefits out way risks or side effects. All the participants collectively agreed and said, there is no major harm.

Dis # 9. My experience is a bit different than what you all say. I experience some side effects related to contraceptive use. I have continues head burning. I feel pain at my hand and feel weak. I use it just because instead of faced problems of pregnancy and child birth, I chose to tolerate these.

Q*. How do you relate contraceptive service use with sexual health? How is your sexual relation pattern since contraceptive use with your partner?*

Dis # 1. Marriage is primarily targeted not only child bearing but to fulfil once sexual desire. We all have sexual desire. *How is your influence to have sex relation with your husband? Do you take initiatives sometimes or always wait until your husband asks you?*

Dis #7. I should not be ashamed on this issue and speak it out. I tell all my feeling to men. I feel that men keep secret better than women. When I went to have tubal ligation, they faced difficulties in the procedure and stopped doing it. Then they told me to use injectable one. I said why you tell me about injectable and back asked them. And then I told them that leave it, my husband visits me very occasional, ones every eight days, thus no problem as I have no sexual desire as such. The health worker said at outset there, you will have more sexual feeling as of this time. He informed me everything in calm manner. After that, as the health worker told me that my sexual feeling has greatly increased. You know I am puzzled that my desire has been high. I say this may be a devil spirit, why did it get weak when I was relatively young and now create a trouble up on me. I reminded days back when I said no to sex to my husband. I think the reason for his new marriage was my unresponsiveness to his sexual request. The day came to me and now I face over feeling. So, where to get him (በስተርጅና ጉዴ ፈላ እያልኩኝ፣ እናቱ አፈር ትብላ)? When he visits once every week, he understands that my feeling is highly improved. He says, you are really changed since started using contraceptive methods. He also says to, “now you are my best” and I say him keep quiet, I say to him, “you behave as urbanized man” indicating that I was abashed. I have better sexual desire than before and when my husband asks I say to him I became better in this regard than you who is well fed and looks strong physically. I need more (አልበቃ አለኝ), but his absence from my home for a week is discourages me. I tried to tell my husband ironically about my desire that at my old age I am getting hot. But he yet didn’t clearly understand what I mean about. I am shy to clearly tell him my desire.

Dis #3. I married to old man not matching with me age wise. I am very young when I married to him. Before tubal ligation I was very cool with regard to my sexual desire. But now things are changed, I became very hot. I am not able to tell clearly to him as he is old enough. Had he been young as me, I would have mentioned my feeling openly. He knew me as I was refusing for his request formerly and if I could raise the issue now, he may be amazed. I am worried that if I tell him he may consider me promiscuous and spoil my personality.

Dis #5. When I compare our sexual feelings, my husband is superior. He is strong and usually I feel discomfort during sexual relation. He has limitless desire, but I dislike it as I feel discomfort. I prepare to isolate myself when he wants it. I feel to extent of crying. Whatever feeling of dislike I have, I have no power to discourage him. He does his feeling. When I tell him that I feel pain and discomfort during sexual intercourse, he says where to go then. He advised me to seek medical help but yet I haven’t found it out.

Questions related to children health are already discussed above.

1. **FGD with mothers in Korangoge Kebele**

Profile of FGD participants

| S.no. | Age | Education | # of children | Year of contraceptive use |
| --- | --- | --- | --- | --- |
| 1 | 28 | - | 4 | 3.5 |
| 2 | 27 | - | 5 | 7 |
| 3 | 23 | 10 completed | 1 | 2.5 |
| 4 | 25 | - | 7 | 4 |
| 5 | 30 | - | 4 | 10 |
| 6 | 30 | - | 6 | 8 |
| 7 | 25 | - | 4 | 8 |
| 8 | 20 | 4 | 3 | 4 |
| 9 | 30 | - | 6 | 6 |
| Mean | 26.44 |  | 4.44 | 5.88 |

Moderater: Abraham Alano (researcher) and Yamrot Haile (assistant)

Venue: Korangoge Health post

. *What is it like to live through contraceptive use towards your status with the respect of your income status?*

- - - *What is your experience about economic status as to fulfil your basic need, your children and the family as a whole?*
    - *What is your lived experience like as far as you started to use contraceptives?*
    - *How you experience the situation in improving your income level at your individual and family level?*
    - *Learn the experiences as expressed by the women using as many probes as possible until information get saturated.*

Dis #1. Our children before contraceptive use were not as strong as the time after the service. We bore them in close gaps and they had not get adequate attentions. We couldn’t give enough breast milk to and better care. The reason was, they born in very close gap. After contraceptive services not only my children are attractive but also I look healthy and attractive.

Dis #2. I got enumerable benefits from the contraceptive services. For example, before the service I gave birth to my elder child whom I did not breast fed adequately. The reason was that my next pregnancy happened suddenly. My second child is stronger and healthier than my first one as he got better care than his older brother. That is why I persist despite some women telling me to stop contraceptive use. They say that contraceptive methods make some users tiny, unhealthy etc… But, I tell them back, I know the benefits I got since being on contraceptive method. I say to them that it is from Our Lord that this service came to us, the poor. Before contraceptive use it was not possible to easily move out of my house as I have to take care of young children born year by year. I am using the service as I well understand its benefit in my life.

Dis #8. Formerly, it had been our experience to get pregnant year by year. As a result, children born that time were mostly unhealthy and malnourished. Thanks to our Lord now we got rest. Our children grow well and our mind is in peace. In past times they look clumsy, malnourished despite feeding them. Now the situation is different and our children are healthy. They perform well in their schooling and growing well. First, I received implannon for three years and when the time due, I removed it and now on jadle, which works for five years. It is four years since I put on jadle. The benefits of contraceptive service are beyond our imaginations. We have got remarkable life benefits. We have narrow land plot. Using this service, we are trying to balance our children number to our land holding capacity. Thus, the service helped us to regulate our children number, in averting untimely deaths of our children and improving their educational performance at school.

Dis #4. I took contraceptive method from Yirba health center first. After well recognizing the benefit, I said to myself what if I started earlier at the level of two children. The service has helped me in many ways. I had one difficult labour which had threatened my life. If I had not started contraceptive method use, I would have died this time due to pregnancy related problems. Thus, above all it has saved my life. During the difficult labour what I faced was retained palcenta. Nobody has taken me to health institution. The retained placenta expelled by itself the second day and bleed massively. That has clicked me to be on contraceptive for fear that subsequent pregnancy would carry more risk. I know similar incident which had ended in life loss. My husband’s sister was died in similar condition. They took her to Yirgalem hospital after long delay with retained palcenta and at her arrival in hospital she lost her life. You see, God has brought this service to avert such premature death of mothers. I really understand that we have got many benefits and averted maternal death that may occur as a result of unwanted pregnancies.

*Tell us your lived experience of contraceptive use towards owning resources, improving your livelihood and engaging in income generating activities.*

Dis #5. It is clear that when I have many children I am forced to remain in home taking care of them. But in contrary if I have limited number of children I can go out of my home to market and involve in trading to generate some income. I also can involve in other income generating activities such as growing garden vegetables and sell it to boost my income. Since the time I started using the contraceptive method, I got enumerable benefits. It is about 10 year since I have started method use. Since then I feel healthy except that I got malaria attacks occasionally. Before the service use, I used only to watch others who went to market and generate income. Now I am also doing the same to what others did before. My children are now grown big and I am free to move to market place to involve in merchandised activities and generate what I can. I also involve in other local and private labour work from which I earn some income for my family. Earlier we were not comfortable to go out of our house as we were emaciated and shy to be seen before people. Now we feel free to work, move or go to different place such as social gathering. Before contraceptive use one of my neighbor ladies calling me “tutuluma, ‘xuxulumma’, Sidamigna vernacular equivalent to “putting in close sequence or order”, as to indicate how close my births were. Her intimidation like ironic speech has awakened me to use the services. Now I am happy that my last child is in school, am free from the burden of pregnancy and child birth. I really thank the government to give us this opportunity.

Dis # 6 My early children were all females and I was going for male which ended up me in having many children. I gave five consecutive female children births and annoyed on the happenings. Then, after the fifth birth I started using contraceptive method. Immediately I got peace in my life. When I return from outside, all my children want to be hugged but whom I can give priority or whom to leave out, that was puzzling situation for me earlier. After long gap, I got pregnant again with desire to have male child. My father also was urging me to have more children out of which to see male child. He thinks traditionally as female are outsider, they will go to their husbands, and thus the house will be empty. My father wanted to curse for the fact that I was using contraceptive method. For fear of and respect to him, I discontinued contraceptive use for two years and got pregnant. Then fortunately I got baby boy. It has been four years from my last birth. Had I had the same level of awareness about contraceptive that time, I would have stopped child birth at my second birth.

When I gave birth to a baby boy, my entire neighbour surrounded me as if I have never given birth before. There was big celebration, worship and thank giving program. I thought by saying that is this only time that I started to give birth? What about the former six times burden I had? I praised my Lord giving this chance. Now my son is at school. I have really benefited from contraceptive use. When I gave my ears to ill- advice of my neighbours, I was harmed. As of I got awareness toward the method, I am enjoying the benefits of the service. I had experienced being pregnant within six months of previous birth. You can imagine that having a six months child at your arm and another in your womb. I used to cry why God has done so that time. That time has passed and we are now able to space our pregnancies.

Dis # 7. I gave birth to five children and said to myself why I should bear more than these children knowing I have no enough land. With this feeling and hearing the information from the health extension workers, I started using the services. Before this service, if we went to market, we retuned half accomplishing our tasks as we thought of that our young children were crying. Now we are thankful to our Lord. Earlier time we were unable even to wash our clothes. Some people say that why do you take contraceptives, once you bear, a child can grow on his own way doing labour jobs. However, I said no to their ideas as I know my capacity. Others may do so because they have enough land, where as in my case I have no enough. I take care of my children doing small businesses. Therefore I am benefited by using contraceptive method. *What visible benefit can you mention for us that you hadn’t had before but you have now?*  I provide all my five children with basic supplies which I get from the small business I have mentioned earlier. This means unlike the previous time, I have got adequate time to go to the market and generated some income.

Dis # 3. I have three children now. After giving birth to the first child I took injectable contraceptive for seven years. Then I gave birth to my second child and after that I also used the same contraceptive method. I got pregnant for third time after stopping contraceptive use. After I gave birth to the third child some people misguided me to simply stay feeding her child without contraceptive method. I gave my ears to their suggestions despite my good contraceptive experience. Hence, I got pregnant unknowingly. I became furious and wanted to abort it. By the time when I went to the health institution, they said that the pregnancy is beyond termination stage. As the will of God that pregnancy ended up in stillbirth. I saw that women who bear closely do not have to eat even during the time of their puerprium (post-partum time). They eat maize porridge which has little nutritional value for such woman.

In our culture those women with good standing economic status fed well, their husbands slaughter animals for them. They eat well to maintain their health. But poor women do nothing. When we space pregnancy we get time to prepare all necessary inputs for the time of our puerperium. Poor women only eat dry bread made of maize floor and drink coffee. They have no milk and or butter. I see very great change since the last 10 years. Most women space child birth to seven to eight years. This gives us chance to properly take care of ourselves. We clean ourselves. A mother in her puerperal period smells smart does not have bad odour ‘ su’neadote’ now as she cleans herself well as compared to the previous time. Formerly, a woman in her puerperal time smelled foully but this time there is no difference between other women. Most of women here space pregnancy but not totally stopped. However, very few have done tubal ligation. *Can you tell your experience how you started using injectable for seven years before giving birth for the first time? What challenges you came across?*

Sure, many people have challenged me. My father was particular person to greatly challenge me. He got male children at his late age. The former wives gave him female children and after marrying to my mother he got male children. Taking his experience in to account, he never says enough to male children. Now I have two male children but for him these are nothing or less. Other neighbour people also oppose contraceptive use before any child birth. More seriously, they oppose injectable ones as they feel that injectable may make sterile or infertile. By with standing all the influences, I used injectable method until I complete my secondary education. After that I temporarily stopped the method use and without any problem I got pregnant and safely gave birth. *Do you mean that null para women can use contraceptive methods?* Yes, one has to plan when to get pregnant and give birth. Completion of education and other urging life events before giving child birth worth in many ways for women lives. Therefore, I encourage others to follow my experience. I know many women like me took injectable methods and when they wanted to be pregnant, they did so. I share information for those women who frequently give birth to use contraceptive methods including injectable ones. But I am not stressing the issue for null para mothers. *Why are you ambivalent about teaching the null para mothers? Are you not confident on its outcome?* Though I haven’t faced any problem by using injectable method for seven years before my first birth, I am not 100% sure about its outcome in null para mothers. Thus, I am not as strong as I inform for the mothers who have already given their first child birth.

Que 2. *What is your lived experience related to your educational status or your family members including your children?*

- - - *What is your experience related to your schooling situation, your children, particularly your daughters if any?*
    - *What is the experience with female education related to the contraceptive use? How do you explain the importance of contraceptive use in relation to sending your children to school?*
    - *If you haven’t reached your educational destiny, what do you explain for us about it as related to the availability of contraceptive methods by then?*

*Do you think that it could have been different than what you experience now? In what manner? Can you tell us in detail about that*?

Dis #2. I am really aggrieved for the fact I was not educated and as a result all my children both females and males go to school. My first female child and her younger both are grade six now. I do not want my children as illiterate as me.

Dis #4. I am not educated and unable even to sign. This is true for many of my age women. We send our children to school for the fear them not to be back warded like us.

Dis# 1. When you see us, we are young but as we are illiterate, we know nothing. I don’t want my children to be as I am. Thus, I send all my children to school. One of my children is grade 10. He attends his school at Yirba. His younger (female) is grade seven; she attends her school here in our kebele. My third one is grade six. Despite our limited resource capacities, we send all of them to school. We have recognized that educated person is not abandoned simply.

*What is your experience in treating both male and female children in equal perspective? At your young age it was known that you were serving your brothers instead of caring for yourself. What is the experience now?*

Dis #5. When I planned to send my only female child to school, might people around me say who would help you in your work? I was convinced by their words and leave her from sending. When her father came from his way asked me why I did not send her. My child on her side said to me why you resist me, do you want to make me as illiterate as you? She bought her school materials by herself and started her schooling. We are sending our female students like that of our male ones.

Dis #4. In my case leave alone prohibiting my female child from schooling, I have great dissatisfaction in my life as I am illiterate. I feel that why I remained ignorant to challenge my parents about schooling. If I had challenged them to send me to school that time, I would have done better in my life. Taking a big lesson from my life, I never let them miss their class. I encourage all my children go to school and seriously follow that. I have four children, two males and two females. All of them are in school. They are grade 8, 6, 4 and 1 respectively to their age. *To what extent do you care for your female children other than schooling? What is your experience in instructing and guiding in their reproductive health issues?*

Dis # 5. My first daughter is grade 10 and I made her youngers to repeat their classes twice with the intention of making them understand well before they progress ahead. I have great ambition to send my children to school. I got big lesson from my life. I was encouraged to send my children to school looking into my cousin’s support in reading for me my vaccination appointment. As I was illiterate usually take my vaccination card to my cousin in order to remind me about my appointment. From this I wanted to send my children to school. Now I am not going to any one’s house for such services. My children are able to read everything for me. Despite some economic challenges I have, all of them are at school now. *How do you relate contraceptive use to sending female children to school?*

Dis #9. My first daughter married when she was grade seven. Her younger sister also married like her when she was grade seven. My third son is now in school. We send our children irrespective of our life burden. I came to this meeting after sending them all to school. *How do you explain the status of women before contraceptive services including reproductive life of your mothers?*

Dis # 9. Our mothers bear children by being covered by heavy blanket with the assumption that they have to deliver by sweating. Our mothers gave all their births at home. Labour took longer for them. By their time they were not allowed to open their thighs during labour which may suffocate the fetus and my danger him. But this time when labour sign starts, we go to health center. Old mothers still say to us that opening thighs during labour at health institution is embarrassing. They say this because they don’t know the consequences of doing so. In contrary to the old days, this time most people have good understanding about modern health services to the extent that our husbands are reminding us about appointments for either ANC or FP services use. *Culturally Sidama men have experience of engaging in multiple marriages formerly. In this regard, what is your current experience to discuss the issue with your husband openly and reaching to mutual understanding and limiting to single marriage?*

Dis # 6. When my husband married me, he had strong love. But when I started to give birth, he was keeping him distant from me. After use of this service, I saw big difference. Now I got time to take care of myself, maintain my cleanliness and seem attractive than before. In past when I deliver, I was forced to stay away of our regular bed. Temporary bed is being prepared from the leaves of ‘inset’ “hashuchcho”on ground. Due to baby’s urine and uncleanliness, worms are growing beneath us. Since I started using contraceptive method I have been free from such circumstance. Now I stay in clean bed with my husband with wonderful peace and stability. My husband is happy and his love and respect to me has immensely improved.

*Tell us your sexual practice with your husband. Do you discuss the matter about match vis-à-vis mismatch? The mismatch may lead him looking for other wife.*

Dis # 2. I abstain from sex for about ten days after initiating contraceptive use. I have no problem and got enormous relief.

Dis #3. When a lady first lie on the ground bed, it is shameful for her to go to bed (to her husband), unless he invites her. When she stay away from him, he may look for other sexual concordant outside wedlock or look for additional marriage. But if he invites her to his bed, she never refuses. In our culture a woman never approach her husband in two months’ time post-delivery but this time she shares bed with him after forty five days. Some people say that there is no problem for sex after 10 days post-delivery but in my opinion it has to be at least forty days. Contraceptive use has created nice relation between husband and wife.

- *What is your experience about having better nutrition and timely feeding status after contraceptive use?*

Dis # 1. With contraceptive use we have better time to run here and there engaging in income generating activities. We eat what we got from our efforts. I can say that now I am doing well in overall status including my health. I feel I am charming this time.

Dis #2. In old days people had better food storage but no nutritional literacy. Though we don’t have huge food reserve as past time, we have better awareness and eat properly what we get from various sources. Contraceptive use has created better opportunities for us to properly use our resources and feed better.

- *What is your experience in visiting health institutions and why you visit them? Tell this in relation to contraceptive service in particular and over all aspects in general.*

Dis # 3. I visit health post to receive injectable contraceptive every three months. Otherwise I go to hospital to ask sick people. I am okay for my health.

- *Tell us your health experience including your family with more focus on the health of mothers and children.*

Before the commencement of contraceptive method we had rampant maternal and children deaths. Now there is no such issue. Even we see pregnant women very occasionally. Some women feel ashamed when they get pregnant this time. In past you may not get these numbers of non-pregnant women. This is good indication that to what extent contraceptive method has eased us.

Dis #3. Child death is significantly decreased. We have never heard such deaths this time except that a young mother referred to Hawassa and her newborn was passed away. Her mother had retained placenta and stayed longer at her home being attempted by traditional birth attendant. Finally she was referred to Hawassa leaving her newborn at home. The new born might have died due to hunger.

Dis # 8. In my opinion, contraceptive services have far –arching benefits ranging from mothers, children, family and the nation.

1. **FGD site: Watera Gendo health post**

District: Wondo Genet

Profile of FGD participant women

| S.no. | Age | Education | # of children | Year of contraceptive use |
| --- | --- | --- | --- | --- |
| 1 | 28 | - | 6 | 3 |
| 2 | 27 | - | 5 | 7 |
| 3 | 30 | - | 4 | 3 |
| 4 | 35 | - | 3 | 1 |
| 5 | 30 | - | 6 | 4 |
| 6 | 30 | - | 8 | 5 |
| 7 | 25 | - | 5 | 2 |
| 8 | 30 | 3 | 5 | 2 |
| Mean | 29.4 |  | 5.25 | 3.34 |

Moderators: Abraham Alano, researcher and Yamrot Haile, assistant

*What is it like to live through contraceptive use towards your status with the respect of your income status?*

- - - *What is your experience about economic status as to fulfil your basic need, your children and the family as a whole?*
    - *What is your lived experience like as far as you started to use contraceptives?*
    - *How you experience the situation in improving your income level at your individual and family level?*
    - *Learn the experiences as expressed by the women using as many probes as possible until information get saturated.*

Dis #1. Our life after contraceptive is cool. We have got peace at our houses with our husbands. Before the services, we used to give births yearly base. That time the gap between two births was narrow. Subsequent child comes before the older one getup from the earth. Life after contraceptive use is different. A child born after the service has got enough time for breast milk. He/she brings seat for me to sit down and give him breast milk. Now I am able to space child births for more than four years and my neighbour got big lesson from my life. My children are doing well in schooling and their health status. They have got adequate breast feeding.

- - - *How you experience the situation in improving your income level at your individual and family level?*

Dis #1 Cont. Our life situation is improving. My income level is better now as compared to the former time. We have enormous change in our livelihood. You see… um… before this service in two years’ time we give two children births. After the government has brought this service even our blood has changed (appearance, wellbeing, overall lives). We had no time to take care of ourselves before this service. We hardly clean ourselves. We eat little as we have to give priority to our husbands, children and we were the last to take our share if we could access after all. Former life was untidy both ourselves and our children. We barely provide enough food for our many children. Just to put this in example, if you have 8 or 10 children, your worry is immense as compared to a mother with less number of children. Many things have changed recently. Government has helped us in many ways. We have got many changes. All our children were used to surround our kitchen formerly but now able to attend their school now. Our children grow and develop well. Their minds are developing nicely as I compare to the former time.

Dis #4. I have seen improvement in livelihood since I started using contraceptive method but I have heavy bleeding.

Dis # 7. By the time when I gave births to many children in close gaps, I was unable to go to market. After I have started using this method, I am not waiting my husband’s hand only. I grow vegetable in my garden such as cabbage and others. I sell some part of these and earn some money and use to eat part of these. I have got time to give attention to myself where I wash my clothes and body. Contraceptive use has also helped me to assign my children properly and provide their requests. Before contraceptive use, I had many children in my house that surrounded me in the kitchen. Now they have grown and moved to various places. No one now burdens me like before as I spaced child births. We graciously care for them unlike the previous time.

*Please try to relate how contraceptive service contributed to the improvements in your livelihood?*

Dis #4. My health status was not good before. I had poor health and felt sick. Now I am okay. Before contraceptive use, I experienced lengthy menstrual bleeding that stay up to 10-15 days. Since I started using contraceptive method this problem has gone and my health status is good. My skin color and appearance is improved and I feel healthy now. I have faced no problem as I lived in contraceptive method.

Dis # 5. First of all I want to praise our Lord for the availability of this service in our vicinities. Next, I also thank the government for giving us this opportunity. We have got many benefits from contraceptive services. Before contraceptive service we mostly give 2 births within two years. This service has greatly helped in improving our livelihood and our family’s. All my children are now attending school. I used to give breast feed only for 8 months before contraceptive use but now I give 3 to 4 years. We live as we plan these days in the era of contraceptive method.

Dis #6. I have got numerous livelihood improvements since I started using contraceptive methods. I saw many pleasant things. I also wait many positive situations in the future. With the help of our Lord, I have improvement in my body weight. With regard to involvement in income generating activities, I equally involve in agricultural production. When my husband produce one quintal, I do the same. I sent one of my sons to Hawassa to attend his school there. If I am pregnant, I cannot do this. I do many things, produce many things and sell them to boost my income level. All these have been materialised as I am living in contraceptive use and averted unwanted pregnancies.

Dis #2. I got rest since I have spaced pregnancies. I have poultry farm. My son is helping me. We are in good situation. The number of cattle is increased now. I grow garden vegetables which boost my income level and I use for my family feeding. I only buy oil and salt from outside market.

*What best practice do you share with your neighbours about contraceptive use?*

Dis #3. Yes we discuss about contraceptive services among our neighbours during coffee ceremonies. We openly discuss what benefits we got from the services. By doing so we are converting many non-users to the services. My daughter is now using contraceptive service and continued her schooling which she dropped earlier. There are some also with intention to use the service.

*What is your lived experience related to your educational status or your family members including your children?*

- - - *What is your experience related to your schooling situation, your children, particularly your daughters if any?*
    - *What is the experience with female education related to the contraceptive use? How do you explain the importance of contraceptive use in relation to sending your children to school?*
    - *If you haven’t reached your educational destiny, what do you explain for us about it as related to the availability of contraceptive methods by then?*

*Do you think that it could have been different than what you experience now? In what manner? Can you tell us in detail about that*?

Dis #8. I have both male and female children. My older child is grade 9, his younger is grade 5, and the third one is grade 4. I remained illiterate due to my parents’ ignorance to education. But I got enough lesson from my life and encouraged to send my children to school. I am waiting to send young children to school when their age allows.

Dis #7. Since I started using contraceptive service, I send my children to school. They attend school properly and I support them to be good in school. Everything is going well since I started using contraceptive method. *Can you tell us your experience in detail about female children education as your area is high land?*

Dis #8. I have three female and five male children. All of them are attending schools equally. I equally encourage and support in their educational endeavours.

Dis #5. You see…, as I am illiterate and use my finger to sign. I said this should never happen to my children. Therefore, I send all my children to school and treat them equally. If I am pregnant or have young child, it would be difficult for me to send all of them to school. Some of them may be forced to stay home helping me. Now I am free and take care of domestic work by myself and sent my daughters to school. Another important issue is the school is in our kebel and schooling is based on shift (half day). This is also conducive for us as children stay half day in school and half day in house supporting me.

Dis #6. I also sent all my children to school. My first child is grade 10. Two daughters following him are grade 8 and one is grade 5. All of them are treated equally. I see both males and females equally. I learnt enough from my life. In our time parents were saying that female should not go to school. Investing in female is considered nothing or enriching/ prospering outsider. This had left me in illiteracy and forced to sign by my finger. I feel ashamed when signing by finger and being illiterate. We said let the situation end here and we support our children in their schooling strongly (ወገባችን ታጥቀን ነው የምናስተምረው). We say don’t be as blind as we are to our children (የኛ ዓይን እንደጠፋ የናንተ እንዳይጠፋ).

Dis #1. We encourage our female children not to miss their school even at the time when we are in our puerperal time. We look for someone who would support as in stead of letting our daughters miss schooling. We took into consideration that missing one day’s school has great impact on their academic performance and if they miss exam it is difficult to cope.

Dis #3. As it has been said earlier, they support us in our domestic work. But they do this without interrupting their schooling. As schooling is a shift base, they help us in the morning if their shift is in the afternoon. They work collaboratively and nothing harms their schooling.

- *It is good that female children are attending school but we want to learn more about how the level of support and follow up as compared to their male counterpart and evenly distributing domestic work and arranging study time*

Dis#5. I never hold against any child from going to school. I also never order my daughter to work giving up her study. My house is close to school. They all study after school. I offer food for both my daughters and sons equally. I advice my daughter saying your brother’s education will serve only him. It has nothing to do for you; therefore, you have to strongly handle your study.

Dis #2. I made my daughter to discontinue her school for one year when I gave a birth. She cried as she was forced to quit her schooling. Her friends came to me and complained on her friend’s situation. I promised for my daughter and her friends not to repeat the same mistake even I bear twins. Thus, our daughters are highly encouraged on their education. We want them continuous this way.

Dis #7. My children are young. I mean they are young to share domestic responsibilities. They are in school now. I do all domestic activities alone. I do not require their support at this time. I encourage them to focus on their education. I prepare food for them on time and keep my cattle. So far I am doing well and nothing happened against their education.

- *Could you explain status of female education before and after contraceptive service use taking into account the time of you mothers and yours?*

Dis #4. I heard that my grandmother died on labour. As a result she grew in difficult situation. She shared her challenges of growing without a mother. I made my contraceptive use secrete for her and disclosed recently. When she heard that I am on contraceptive methods, she appreciated it. She also mentioned that your era is wonderful. If we had contraceptive service long back, I would never miss my mom untimely. Therefore, contraceptive service use enables women to avert unwanted pregnancy thereby create opportunity to use their time wisely and let their children stay in school. When a woman is overloaded with domestic work, she never thinks of sending her children to school. The situation is worst for the female children as they are close to their mothers.

Dis #1. Currently labouring mother visits health center when she sees a labour signs. In the past women stay at home up to 8 days in labour without any medical helps. What they did was only massaging her abdomen. With contraceptive service many things have been improved in our livelihoods.

- *Do you have any sort of regretting that you haven’t educated?*

Dis #2. For example before contraceptive use, I stayed many days on my first labour. My second labour was also delayed. I stayed for two days with the head out. We have no as such experience recently. We have heavy bleeding after births previously. Many complications related to child birth have claimed the lives of many mothers before the inception of contraceptive services in our area. Many of them live with the squeal of untrained traditional practitioners.

Dis #5. My first birth was complicated and stayed for long time. That day we had no health institutions nearby and only to be carried by human shoulders. Now situations are different than the former time. We have passed many miserable conditions. Now we really thank the Lord and the government. We are free from such problems and complications related to labour as we are on contraceptive use. The service has created better environment for us. It has been a history to stay in labour for 10 days. I would never face this day if my life had passed away that time. Thanks to the Lord, now I am seeing the difference. By the time before contraceptive, we were largely ignorant for many things and lived in substandard means/ oppressed way. We didn’t have enough time to think of our personalities, desire and needs. We lived in situations like having too many pregnancies and children were we were overburdened. As a result, we were forced to submit our humanness to our problems and neglected our personhood.

*What is your experience related to your health status?*

- *In terms of your overall feeling good?*
- *Your nutritional status, weight, types of diet consumed*
- *Visiting health institutions*
- *Reasons for the visit*
- *Any illness that made inactive, bed ridden, etc…*
- *What is your experience in your health status in relation to your contraceptive use? How do you explain this in terms of improvement or deterioration?*

Dis #8. I live better life with contraceptive use. I feed better and live in hygienic condition. I wash my body and clothes regularly. Our lives were not like today. In past, Sidama men marry up to seven wives. Some of the reasons for this were the older wives became untidy as caring for their young children. Their time all occupied with the care of children and gave little attention to their wellness as well as that of their husbands. Women’s’ lives were overwhelming where they have little time to take care of their cleanliness and remained unattractive to their husbands. Consequently, husbands were looked to marry new wives. But situations these times (in contraceptive era) are different. We maintain our cleanliness and give value to our personalities. We also keep our home clean and attractive. Our beds unlike the previous time are neat and comfortable. We are able to care properly to our husbands providing food in time. Overall we are doing better than the past time and converted our husbands stick to us. We are lucky when compared to our mothers. They lived boring lives, bitten by their husbands, rampant domestic violence and huge domestic workloads.

- *You said with the intention to avoid pregnancies, you also keep aside yourselves from many issues. Is that true?*

Dis #7. It is true that our lives formerly were not satisfying. We were strange to our husbands as we were not attractive enough to feel the desire of our husbands. When they failed to get their desire from us, they tempt to other women. Some start adultery outside and others marry more wives. After we used contraceptive methods, a woman spaced pregnancy, maintained her cleanliness, put on fragrant ointments, cosmotics and perfume, eat well and built her body, has good mattress thus pass her time with her husband in bed properly. Now we understood why our men married to seven and more as the one in the house looked wildish, unattractive and skinny, as result swing their eyes to outsiders. Today we give time to ourselves and our husbands receive our contraceptives on time and avoided unwanted pregnancy, thereby we live peaceful and lovely lives.

Dis # 2. Before contraceptive service, we lived almost in pregnancies and child births. We were unable to keep our cleanliness and health properly thus we were never motivating our husbands for sex. They seldom require us for sexual relations. Even if they want us for sex, they were not happy upon us. Now we are free and do whatever we want or in any way we want with our husbands.

- - *Did you ever visit health institutions before? If so why you did it?*

Dis # 1. Yes I visit health institution for vaccination and contraceptive service. All other participants said the same. *Any of you have visited to hospital for medical problems?*

Dis # 3. Sometimes I feel pain and discomfort around ribs. When I went to health institutions, they said that it was gastritis, and kidney stone. Yes I have some medical problems.

Dis # 7. Yes I had cough which finally diagnosed as bronchial asthma. This condition is aggravated during pregnancy. Now I am okay as I am not pregnant now and not want to be pregnant soon. Therefore, I testify that contraceptive use has helped me in avoiding such symptoms.

Dis #6. I was also said that my lung was full of fluid and undergone surgery. Since then I am doing better though have some discomforts.

Dis # 4. During pregnancy I feel chest tightness and bulging of eyes. After contraceptive use all these problems were fading.

*What is your experience related to the health status of your children?*

- *What is your experience in terms of child growth and development? Maintaining proper weight and height,*
- *How you explain their health status in terms of acquiring disease or illness? Visiting health institutions or other remedies*?
- *How these contribute to your health and general wellbeing? More probing…..*

Dis # 2. Our children are now feeding well and growing well. They are able to feed at least three times a day. *Can you tell as any experience related to neonatal and young children death?*

Dis # 1. I haven’t experienced child death so far. They grow well and health. All they have received vaccination given for their ages.

Dis # 4*.*  I lost one child at the age of eight months. I regret that if I had followed vaccination and growth monitoring services properly, I would have never lost her. Since contraceptive service use we get pregnant only when we want to be so. Therefore, we follow antenatal care and when we give birth follow all child health services including vaccination. Now they grow well. No major health problems affecting them. Generally our children health status is well as compared to the previous time. All other participants shared the same idea as stated here.

1. **FGD site: Degara health post**

**District: Dale**

**Profile of FGD participant women**

| S.no. | Age | Education | # of children | Year of contraceptive use |
| --- | --- | --- | --- | --- |
| 1 | 25 | 5 | 5 | 8 |
| 2 | 22 | 8 | 3 | 4 |
| 3 | 20 | 4 | 4 | 3 |
| 4 | 20 | 9 | 2 | 2 |
| 5 | 25 | 6 | 5 | 5 |
| 6 | 26 | 6 | 5 | 9 |
| 7 | 20 | 4 | 2 | 3 |
| Mean | 22.57 | 6 | 3.7 | 4.86 |

Moderators: Abraham Alano, researcher and Yamrot Haile, assistant

*What is it like to live through contraceptive use towards your status with the respect of your income status?*

- - - *What is your experience about economic status as to fulfil your basic need, your children and the family as a whole?*
    - *What is your lived experience like as far as you started to use contraceptives?*
    - *How you experience the situation in improving your income level at your individual and family level?*
    - *Learn the experiences as expressed by the women using as many probes as possible until information get saturated.*

Dis #1. When we compare situations in the past with today, there are many changes. Before contraceptives use, women bear children closely. They faced many problems to the extent that until worms appear from their bodies. After the government has introduced this service, we and our children live healthy lives. We are clean and health so as our children. Now their appearance attracts us. They are healthy and well. When one’s life is improved, others follow by saying what has contributed to the betterment of Ms. X’s situations. I spaced pregnancies and child births naturally. Even before contraceptive service use I gave births every four years gap. Thus, I had good opportunities to nurture my children. Now I lead fearless life as I am using contraceptive method. There is no fear of unwanted pregnancies. With contraceptive methods our live is glowing. We got peace, rest and stability as well as we are neat now. Both ours and our children’s’ lives have improved. *How do you explain livelihood improvements?*

The same discussant:- Really our lives have improved in many ways.

Dis #2. I gave two births in two years. Do you think was that good? Before I breast fed my first child, the second one came suddenly. I stopped giving him breast milk by force. After that by the will of our Lord our government brought contraceptive services. Thus, my life has greatly improved since I have started using contraceptive service. I appreciated the benefits as it enabled me to space or postpone pregnancy. I got pregnant by plan and gave birth for healthy child. After third birth I used the method for the last eight years. Now my third and last child is 9 years old. She is now in school. Before contraceptive service our live were not like this time. *How do you relate your contraceptive use towards enabling women in income generating activities?*

Dis #3. I would like to thank my lord for this chance. Now I am able to delay pregnancy and started to generate my own income instead of looking always my husband’s hand. We are able to send our children to school and provide proper care in feeding, hygiene and clothing. Our knowledge toward contraceptive services has improved that increased motivation of method use. By spacing pregnancy and child births, we peacefully mange our domestic activities and the care to our children is improved.

Dis #4. I was ignorant about child handling when I gave birth to my first baby girl. I faced many challenges on how to up bring her. She is now grade nine. I started using contraceptive method after my first birth and spaced for four years. Then I gave birth for the second baby boy. He is now grade five. The recent life after contraceptive as compared to before it is like the distance between the sky and earth. We were largely challenged with unregulated pregnancies and child births with its subsequent burdens before contraceptive service use. I am now take care of myself, my children and involve in trading and other small income generating activities. By doing so, I live decent life when compared with the previous time.

*Is there anyone who has different idea about contraceptive benefits in users’ life?* All the participants said they have no opposing idea related to the benefits of contraceptive uses.

Dis # 6. After giving birth to my first baby girl, I asked my neighbour who is using contraceptive service what I can do to delay pregnancy. She told me to take my baby for vaccination and meanwhile consult health professionals about contraceptive use. Based on that I consulted health professionals on my way to vaccination and they gave me oral contraceptives for a month. After that I shifted to injectables and delayed pregnancy for seven years. I gave birth for my second baby boy after seven years of the first one and my first child is grade 2 now. After that I also used contraceptive method for six years and gave third birth at seven years. Such spacing has created great chance for me to design my own income generating activities. I organized small group enterprise composed of 8 men and 16 women. We got credit from micro credit enterprise to initiate our actions. We are involved in income generating activities and generating our income. We pay credit and save out of our profit. We also gate some share out of the profit to support our daily lives. We mobilize some money from our saving to our domestic expense and in case we wanted to build our residential house etc… All these opportunities are created for us as we are able to space pregnancies using contraceptive services next to our Lord. I am grateful to my Lord for offering me such chance and opening the mind of our government to do so.

*What is your lived experience related to your educational status or your family members including your children?*

- - - *What is your experience related to your schooling situation, your children, particularly your daughters if any?*
    - *What is the experience with female education related to the contraceptive use? How do you explain the importance of contraceptive use in relation to sending your children to school?*
    - *If you haven’t reached your educational destiny, what do you explain for us about it as related to the availability of contraceptive methods by then?*

*Do you think that it could have been different than what you experience now? In what manner? Can you tell us in detail about that*?

Dis #5. I was grade four by the time when I married. Fifth year after my first birth, I continued my schooling being on contraceptive method. Unfortunately I missed the appointment and got pregnant again. After that birth again I started schooling again. Now some of my children are attending school at urban area and I also do my schooling. I know all these are due to contraceptive use. You see; in the past leave alone to go to school being a mother and wife, you have no time and means to wash your body or your children. I really thank the Lord and the government as well as the health worker for this opportunity. The information health workers given us have greatly helped in changing our life styles including contraceptive uses. Contraceptive service has benefited all the family members. We have peace and agreement in our lives. A husband has got relief. If his wife bears children frequently, he has no capacity to cover all the expenses. Since she spaces pregnancies by using contraceptive service, he feels comfortable by minimizing his expenses.

- *What is your lived experience in relation to female education and contraceptive use?*

Dis #3. I have young age children. No one is in school now but I am determined to send them to school when their age is so. I have one female child and I will also send her to school at her age. I have no differing outlook about female education. I feel she has to be educated like her brothers. *What is your belief about female education? Do you recognize that female can be educated as male?* All the FGD participants said yes aloud. We have enthusiasms to support them in their education even if they marry.

Dis #1. My first child is female. She is grade seven now but I let her to repeat grade six in order to improve her academic stand. I warn her not to be absent from school. I feel regretful for the fact that I hadn’t educated. I see my female children above males. I never say her to remain at home helping me in domestic works. I say to myself if work burden become heavy for me my Lord will be at my best side but not to share it with my daughters. I do these all as I want to see my daughters ahead like anyone else holding higher positions, well paid jobs etc… I understand that education is one of the means to ensure my dream and I do believe God will materialize it.

Dis #2. I also agree that female education is very important. To see female education in relation to contraceptive use, when we bear children very frequently or in close manner, we force them to stay at home helping us by domestic work and caring for other children and myself. Contraceptive use has enabled me to space pregnancy and child birth and spare my time to other domestic activities thus I am sending my children to school including my daughters. I strongly encourage my daughters not to miss classes in any case.

Dis #5. My first child is male, whom I breast fed for three effective years. We send him to school at his seven. I and his father collectively support him in his education. His father was stopped his education from 9^th^ grade but now he continued and completed his secondary school. I know that I am illiterate and lacking but don’t want to be back warded altogether. Therefore, I am courageous to support both my husband and children in their schooling. My son is now a graduate and employed in microfinance organization. His younger is female. I have great feeling about my illiteracy status and determined to send my daughter to school. I said to her; please follow your brother footstep and be strong. Now she is attending second year college education. Her younger one is male and also attending school. The remaining children are very young and I do advice to be strong in their education. I know that in old days our parents did not treat female and male children equally. I took lesson from that as how I remained back, thus giving equal treatment for both my female and male children. I encourage them to study hard. I know that I did all these as I avoided frequent pregnancies due to contraceptive method use. By spacing pregnancy, I am able to handle my children properly: provide better food, maintain their cleanliness, buy clothes etc… It is all about by the support of the Lord and the government establishing the services in our kebele.

- *In past, mothers were proud of their daughters’ marriage or readiness to marry (ደርሳልኛለች)*. *How is your comment on this issue relating to female education?*

Dis #6. Unlike the previous time, we are aware that early marriage ruins the life of a woman. We discourage early marriage and tell our daughters to stay in school until they will go through all necessary steps.

- *How is your stand if someone comes with high level of dowry for your daughter for marriage request? Do you have the strength to oppose this?*

Dis # 7. I never accept such request and I never allow my daughter to accept untimely marriage irrespective of the amount and value of dowry. What I will do is to fulfill her necessities and advise not to raise her eyes those who marry early. I tell her the danger of early marriage in her future life, therefore never to be cheated in such temporary presentations. I understand that family contribution is immense in this regard and as her mother I will do all necessary support and follow up.

- *What is your experience about sharing with your daughters on female secondary sexual characteristics, such as menarche? Did you tell them and where from you heard about this issue when you were young?*

Dis # 3. Nobody has told me about menarche but I saw it suddenly. *What about you? Do you tell your daughters?*

Dis #6. They get information about menstruation. They know how to keep themselves clean and wear clean under wears and sanitary pads during menstruation. This time most girls know about the onset of menarche from school. Even they don’t tell their mothers. They manage it by themselves. My first child is male. He is studying in the university. He will be graduating at the end of this year. His younger is female and now attending grade 10. I am happy sending her to school. Because I want to see my daughters behaving as educated women; handling them in modern styles. I feel jealous seeing other girls having made their hairs, appear charming and beauty. I want also to see my daughters in such appearance. My third daughter is now grade 8. We share reproductive information each other. I give them my personal experience and they bring to me modern thinking. By doing so, we enrich our understanding.

Dis #4. My experience in female education takes me back to remind what had happened in the past. In the past including myself, our parents said to us that female education is meant to equip her how to handle her house. She has to be competent in domestic work and how to handle her house. They said how on the earth a female goes outside. They harmed us in keeping us away from schooling. I am illiterate and end up in marriage. The reason was that my mother didn’t know the benefit of female education and didn’t send me to school. We had no capacity to say no to their decisions. They were saying no female education; “female and dead body should leave the house as early as possible***” (ሴት ልጅና ሬሳ በጊዜ መውጣት አለበት),*** consequently ended up in early marriage***.*** On the other hand, taking adequate lessons from our last time experience, we send all our children to school not separating female from male. We keep on advising our daughters to be wise and strong in their education and be farsighted. Some of us are attending school despite our family responsibility hoping to complete it. Now, the government has put wonderful affirmative action to females as compared to males. So, we encourage our daughters not to miss their schooling in any case. We equally treat both female and male children.

- *What is your opinion about if contraceptives methods were available during your mothers’ time? Would there have been any difference on their lives?*

Dis #2. For the issue about comparing the previous time with the current status, there are clear differences. During our mothers’ time everything was dark. They did not know about such issue. They have passed that hardship time in the help of the Lord. It was a time of ignorance. They knew nothing. Our time is full of lights. We live in shining world. In this my short age, I experienced that female school attendance was considered as a deviant. Those who share this idea say that the best way to educate female is keeping her in house and traditional train how to handle her house. She has to be excellent in food preparation coffee boiling inset processing and other domestic works. They considered our school attendance as if it was signs for stupidity or laziness. When I remind that at this time it is like they were in darkness and now we are in bright light. There is remarkable difference between the past time and the recent one. My mother was bearing children on yearly basis. She sometimes didn’t know even she was pregnant. While she was breast feeding, another pregnancy was already created. She died of massive bleeding after birth. When I think back, had this opportunity was there, we would never lose our mother untimely. Then I start confessing that it was the deed of Lord that I may be in conflict with him. We have seen many positive things. God has given us many pleasant things now. In past, when we were in post- partal period, we smelt offensively that people easily identify us from distant. But this time, we wash ourselves like anyone else, put on pleasant deodorants and perfume. The old time is changed and we are now known for nice fragrance than the old offensive one. Some are about to mistaken that is this one young girl or married one. This is all about the contribution of health services including contraceptive ones.

*What is your experience related to your health status?*

- *In terms of your overall feeling good?*
- *Your nutritional status, weight, types of diet consumed*
- *Visiting health institutions*
- *Reasons for the visit*
- *Any illness that made inactive, bed ridden, etc…*
- *What is your experience in your health status in relation to your contraceptive use? How do you explain this in terms of improvement or deterioration?*

Dis # 2. I married at my young age and gave birth immediately. I was ignorant about how to handle my newborn and care for myself in postpartal time. I couldn’t properly wash either my baby or myself. As a result some larvae (worms) were scrambling over my body. I feel greatly compensated when I think back. Since the time I have started using contraceptives, I have seen no health problems. I feel okay and healthy.

*In connection to this, in past times it was said that when a woman gives many births and become weak, the husband left her with her children and marry to another woman. Is that true?* **Yes! All exclaimed.** *What do you think he went so? What are the situations/ experiences after contraceptive service use? What is your sexual health life this time as compared to the previous one?*

D*is #6.* Our sexual life before contraceptive was hectic and full of doubts. We never pass nights in bed in peace with our husbands thinking of the burden of pregnancies and births. Our lives now are comfortable and we have good sexual relations with our husbands as there is no fear of unwanted pregnancies.

Dis #3. There is change now as compared to the former time. Unregulated and close births don’t give a mother to take proper care of her and as a result she smells offensive. She shares bed with her young children and they urinate over her. This bad smell does never attract her husband. He develops disinterest up on her and may look to other woman. Since the inception of contraceptive service use, we, women have been able to space pregnancies and delay child births. We keep our personal hygiene and that of our children. There is no as such foully smelling like the previous time and our husbands are not distancing themselves from us. Now the woman appears attractive and neat that a man is also motivated to have good sexual relation with her. We, the women also desire sex when we eat well and feel healthy. But we don’t want to have sexual relation when our body is emaciated. Husbands are also selective in this regard as they do not want to approach us when we are unclean, emaciated and clumsy. Contraceptive use has created golden chances for us to avoid undesired pregnancies; hence our body weight, makeup and appearance seem well.

Dis # 7. We approach our husbands when we feel hot and they understand that we are ready. But most of the time sexual initiation is taken by our husbands. What has contraceptive service done for us is that it has given us time to care for us and avoid unwanted pregnancy. When we are well, attractive and free, our husbands are motivated to be with us and ready for foreplay.

Dis # 5. It is true and I share the idea of former speakers that when a woman keep on bearing children year by year and stay busy with them, she has no time to feed well and care for herself. This time her body is emaciated and she smells unpleasant. She also forced to feed her young child despite her emaciated body status. At this time a woman dislike/hate not only to have sex but to see men. The man may come to home after eating and drinking outside, being sexually aroused, but the woman in the status mentioned above would not be in desire. As a result they end up in conflict at bed. However, now we are in different world in the time of contraceptives. We are grateful to our God and the government for having this service. Now I have stopped breast feeding my 4 year old youngest child. I did this because he has fed enough. Now he plays with other children outside, eat whatever we have. There is no as such problem like former time. My health status and that of my child’s after I started using contraceptive is well.

1. **FGD in Konsore Fullasa Kebele**

Profile of the participants

| S.no | Age | Education | # of children ever born | Period of contraceptive use | Remark |
| --- | --- | --- | --- | --- | --- |
| 1 | 25 | 5 | 2 | 5 years |  |
| 2 | 25 | 4 | 5 | 7 |  |
| 3 | 22 | 7 | 1 | 1 and half |  |
| 4 | 35 | 3 | 7 | 5 and 3/12 |  |
| 5 | 30 | - | 4 | 4 |  |
| 6 | 30 | - | 6 | 3 |  |
| 7 | 22 | 10 completed | 1 | 2 |  |
| **Mean** | **27** | **4.14** | **3.7** | **4** |  |

Moderator: Abraham Alano, Translator and note taker: Yamrot Haile

Date of the discussion:

Time started: 9: 30 AM, time ended: 10: 10 AM

Place of the discussion: Konsore Fulassa Health post

Question 1. *What is it like to live through contraceptive use towards your status with the respect of your income status?*

- - - *What is your experience about economic status as to fulfil your basic need, your children and the family as a whole?*
    - *What is your lived experience like as far as you started to use contraceptives?*
    - *How you experience the situation in improving your income level at your individual and family level?*
    - *Learn the experiences as expressed by the women using as many probes as possible until information get saturated.*

Dis #1. There is clear difference between before and after contraceptive use. Before contraceptive service use I had problems related to child births and workloads. I have also problems related to handling my family members. I was undermined before by many people. Things are different today. Instead of going far to look for the service now I am accessing contraceptive service here in my kebele with minimum walk. Before the health extension program if one needed to use contraceptive service has forced to walk long distance and time and financial barriers were another bottle necks hampered from service use. Now thanks to our God and the government, we receive health information in daily basis. The health extension workers advise us on how to keep our personal hygiene, different types of contraceptive methods available in the health posts and elsewhere, inform the benefits of contraceptive use about spacing pregnancies there by improving the health status of both mothers and children. As a result now I use contraceptive services. I gave too many births before contraceptive service use. I have regretted for not using this service earlier. The reason is that I would have been able to postpone some of my very close pregnancy if I had used the services. Having many children at family level has ruined (አጎሳቁሎኛል) my welfare including health and economic status. It has been a big challenge for me to properly manage all my children. You see how puzzling it is to buy a uniform for one, shoes for the other and sending them to school fulfilling all materials they require. It has greatly affected our integrity.

Contraceptive service has enabled us to get rid of such problems mentioned above. Now we can plan the number of children we want when them to have. If we want to stop at all, we can also choose to do so. This has enabled us to give better care for our children now. Our livelihood has improved as f the time we started using contraceptive method. We dress well and clean our body and cloths frequently. Our health status and general body image have improved. Even we appear charming and seen as new for our husbands who were disrespected and undermined due to our earlier situations (untidy, skinny and foully smelling due to babies’ soiling). Now time has come for us to dress well and match each other and move together. We are renewed, our lives have improved and we feel comfortable (አምሮብናል).

Dis #2. We have seen numerous changes. When I consider our non-contraceptive use time and our mothers’ time together, there were many life problems and bottlenecks. Our mothers had suffered many ill health. They had faced nutritional problems both for their children and themselves. When children born year by year, they had no enough to give them. Every child looked her hands whether she had something to give him/her. The one in the womb also requires her care and support. Lives for women were miserable. But after the introduction of contraceptive services in our locality, women are able to bear children in spaced and planned manner. The older child grown well before the subsequent one comes. He/ she reached to the stage of walking, running and playing with neighbour children before their younger born. We have got adequate rest between pregnancies; the amount of bleeding during labour has become normalized. We feel healthy stronger physically and knowledgeable about various issues including reproductive health. While receiving contraceptive service we have also acquired better awareness and skills an how to care for our children in terms of complementary feeding, providing balanced diet from various sources, their hygiene. Both our mothers time and in our time before contraceptive service use, we had no exposure about proper children feeding practices.

*What is your lived experience with respect household and your economic t as you lived in contraceptive use?*

Dis #2. I know that I would have given births to more children if I were not on contraceptive method this time. As I know its benefits thus timely started to use it.

Dis # 3. What I want to tell you is that I have six children already. I gave three births before I started using contraceptive service and three after I started the use. I did so as I recognized that if I use a service I can plan when to become pregnant. Accordingly I have managed to postpone pregnancies after using the services. When you have space between pregnancies, you really love your children and become eager to see them. When you get rest between pregnancy means you get comfort both physically and mentally. You use your mind to think deeply how to overcome life challenges and set means to generate your own income. Therefore, I tell you that this service has enabled me to go to market and buy and sell commodities and earn some money. More over this time I am able to care for myself, wash my body regularly. I got big rest for myself. When comparing to the previous time, I see many changes in my life. I was disparate in the past due to miserable life circumstances but now everything is good. We have passed those stages and became winner in the grace of our God and the efforts of our government.

In the past when we were untidy and contaminated with our children’s people disgraced and undermined us. Even our husbands did not care about us. They too, undermine and down look us. We were trapped in burdens of domestic work including child care. Our lives were totally engulfed by pregnancy and child bearing. Now, I am happy that there is no young child who will challenge me. As a result I am able to freely move to various social affairs and market. I do so as if I am a young girl. I feel full of energy inside.

*Would you please explain your experiences in relation to economic gain in comparison to your previous time?*

Dis #4. With regard to my involvement in income generating activity since I began using contraceptive service, I involve in various income generating activities and able to feed my children. I go to market to buy and sell in order to boost my income. I also involve in small scale garden cultivation where I produce some agricultural products. These efforts have improved my family income level and contributed to the wellbeing of our children. I am really thankful to my Lord as my children are growing very well.

Dis #5. With respect to our development, sure we have shown great change. Since the time I began contraceptive service use, I strongly involve in income generating activities. As I am not pregnant now and then, I have enough time to go to market and buy and sell different items. I understand that I have to do so in order to improve my family income and livelihood. Contraceptive use has emancipated me from the traps of pregnancies and child care. Thus I run here and there to maximize my efforts to my family members.

Dis #6. If you intention is to hear our experience in terms of economic gain since we have started using contraceptive services, I will put it this way. It is six years since I have started using contraceptive services. For example, during the winter I work in coffee processing site and I also prepare injera for various social ceremonies. I am paid for these services and earn money. By doing so since 1999 E.C., I was organized in the credit and saving association and saved some money. I received some money from the association for house construction. My house is now upgraded from grass roofed to corrugated iron sheet cover. I live a decent life with dignity now and considered humanely both within my family and in my community (ከሰው ጋር እኩል ሆኜ ዘና ብዬ መኖር ጀምሬያለሁ). On top of that, I also directly involve in agricultural production and produce my part. I never look my husband’s hand all the time like as the former time. There is no gain by simple sitting. Is it not? All said “yes”. Therefore, our movement have improved our gains.

Dis # 2. Our parents did never plan the number of their children. Every one of them gave for more than 10 births. They considered that child bearing was the only destiny and sign of development for them. We saw this bright day due to our government and our God. We reached to the level that we can plan the number and time of our pregnancy. Now I plan to have one child this year and post pone/space another one to more than three or four years. When I do so, my capacity to feed my children and for my self is improving. When the number of children is more, the there is little or no food to eat and poverty is at your door. Contraceptive uses not only improved my income status but also my personality, the way I dress, and value myself. I look very young and able to attract my husband’s eyes and heart.

Dis # 1. I born two children. I gave the first child birth before I have started contraceptive use. Then I started to use it. I used for four years before getting pregnant my second child. It is five years for my second child now. Both of them are a support for me now. If I were not on contraceptive use, I tell you I would have given for seven children births. Both my children are attending their school. The older one is grade 2 and his younger one is beginner. What surprises me is that people say to me as they are my brothers. Many people have been confused as if I have never given birth at all. I properly handle my children and follow them while they go to school and come back from school. I also tell them to wash their hands before eating food as a rule every time. With regard to our economic status, in grace of our Lord we do nice. My husband does mixed agriculture. He goes too far place and brings stuff for sale. Then I sell these items in local market and get some profit. I handle the money strictly and when need arise expend wisely. I collect the profit and save it. We have bought goats with the money saved. The goats now multiplied and became many. These all gains are due to contraceptive use as I was able to postpone pregnancy.

*Is there any one with different idea or feeling about contraceptive use saying there had never been any change?*

Dis #7. No there is no differing idea but I want to share my experience. I started to give birth when I was very poor. That time there were no contraceptive services or even no information about it. My first child was fed breast milk only for four months. I started to use contraceptive service after giving births to two children. I started contraceptive use without my husband’s knowledge for the fact that I did not want to be trapped with the burdens of pregnancy and child birth. I gave births to two children after contraceptive use. I gave births to these two children as I discontinued service due to feeling of dizziness. After four births I said to me as these four children are adequate and no more children. But suddenly I got pregnant for the fifth time. Then, I told to my husband that these children are enough for us ( you are not supportive for me) and I wanted to use contraceptive method openly. Since then I started using injectable method openly and now able to care for me and my children properly. I made the previous time life a history. My husband was not supportive and caring for me. When I was overwhelmed with child cares and domestic responsibility and not able to clean, he said “go out, I don’t want you”. Since the time of the establishment of the health extension program in our kebele, our sister health extension workers have been teaching us and capacitating us how to use the services and act in proper manner. Contraceptive service has given me real rest and comfort. It has enabled to send our children to school. All my children are in school. My older child is grade 6 and others are in subsequent grades respectively.

My husband was strong merchant and we have cattle. Our lives have improved but I saw a little reluctance in my husband. He is not motivated to work as he does earlier. However, I recognized to replace him and work very hard to care for my children. Nothing hampers me from doing so. I have no fear of pregnancy or care of young children. I run here and there to diversify income for my household and properly handle my children.

*What is your lived experience in terms of education in your family taking into account to your and your children as you lived in contraceptive use?*

Dis # 4. Truly our parents were not enthusiastic about our education and hadn’t sent us to school. This happened as they themselves were not educated. Tough we are also not educated; we are trying our level best to educate our children. Two of my daughters ended in marriage by dropping out their school from grade seven. I was not in position to tie their legs at home ones they feel they are adequate for marriage.

- *What did you do any attempt not to discontinue their schooling and ended in early marriage by providing proper information ahead?*

I didn’t expect that to happen. It was sudden for me. *At least we have to be better than our parents in sharing information with our children and equipping them in such decisions. Don’t you think so?* Yes you are right. We, the parents, should advise and follow them. But the problem is that, this day children are not like the former one. They want to go on their own way and give little room to listen their parents. They are largely influenced either by their peers or their own thinking. You know, in my case, I have given enough advice for my second daughter as I came across the untimely marriage of my older daughter. But, the opposite was happened. I have one brother in town. He usually says to me that “you remained uneducated” thus you have at least to teach your daughter. My second daughter married on the way to church. We tried to evict the marriage by law but the local elders and church leaders ended in reconciliation.

Dis # 3. I always sense a sort of regret I was not educated. Thus I want to see the fruit of education in my children including my daughters. I know that my chance has already gone. I usually say that oho Lord give me strength in order to educate my children. I always put their education on top agenda. *What is your experience in relation to female education and domestic workload as compared to their male counterpart in your family?* Yes it is true that our daughters have increased load than their brothers. I remember that one’s when I was in postpartum period, I let my daughter to drop her school and care for me and others in the family until I got ready to take over domestic responsibly. After that she was offended and torn her credentials and decided never to go back to school. I am challenged how to let her join school again. Her condition still burns me (የእግር እሳት ሆኖብናል). Still our culture somehow oppresses females. Even after school females enter to the kitchen while their counterpart males go out to play.

Dis # 5. In Sidama culture there is little attention is being given for females. Though it seems improving the progress is sluggish. People mostly say that female child is for outsider, meaning that she move out by marriage, therefore, of her husband’s. As a result attention is given to male child. If a lady born female child repeatedly, her husband disgrace her. However, a mother does not totally dislike female child as she is going to help her in many works. In my part I have recognized the importance of educating female children. I saw practically when I gave my first birth in Yirgalem Hospital by C/S, a female physician was managed me. That has created big enthusiasm in me to send my druthers to school.

*What is your feeling about contraceptive service availability if it was in place during the time of your parents? What difference could you observe?*

Dis # 7. One day on my way I came across rain and wanted to shelter in one house beside the road. When I entered into the house I saw one old man and very young woman. When we started talking, I asked the man how many wives he had and whether this one is his wife. I added also how many children do they have? The man replied to me saying she is the only wife and has given birth to eleven children. And he also informed me that all his children now have attained better positions. Then I asked him what was the secret of keeping a woman young, after having all eleven children. He told me that he has taken good care of his wife. He acted a good support in all domestic activities while children move to school. The man was not only supportive for his wife but was good father for his daughters. He sent all his daughters until all of them posted in various positions. Some of them are teachers and others work in various administrative positions. The woman looked young as now she is free to care for herself and got rest now. In addition, our income is improved that she feeds well (eat and drink adequately). Since she lives comfortably, she confuses people whether she is his daughter or wife.

*You said that you feel good now in many aspects including your health. Let me take you to the indirect way of expressing the health status. What is your lived experience in expressing your feelings, sexual desire to your husband? What is difference you have observed after you started using a service?*

Dis # 6. In the past we lived in a messy conditions. We born children in a close gap, thus soiled with urine and stool of our young babies. When we go to somewhere, our surrounding smelled as if anything spread around. We smelled foully. Thanks to the Lord now there is no such thing. When we started to use contraceptive service we got time to clean ourselves and our cloths. As a result we are able to share bed with our husbands. There is no worry now.

Dis # 2. In the past as a result of ignorance we were harmed by having many births. We are unable to care for ourselves, no time to think of our personalities (what to dress, eat, keep cleanliness). Now everything is okay. Whether we want to go out or whatever we want to do, we can do it. We clean our body, dress well. We make our hair properly. The time we were labeled as foully has passed. We care for ourselves knowing that we were the one who undermined ourselves.

Dis # 1. The matter of sexual relation and love between couple is now a day has received attention both in the church and government sectors. I am aware of handling my marital relation. I have to treat my husband in polite and attractive manner. If I stay away from my husband stating many reasons, I am responsible for disputes in the family. Formerly when I was not clean, I afraid to approach him for the fact that he might not be interested in me. Thus, I had to wait until he calls me. When I behave this way, he may look to other lady. It is common for a man despite having good dinner to quarrel if he had no bed relation (sexual intercourse) in the night. The next morning is full of disagreement. He never says yes if I call. He never eat breakfast or drink coffee. He never supports me in any condition. If a wife disobeys his sexual request a night, a man starts thinking that oho, the lady might have begun to adulterate. He automatically considers it as she disrespected him.

However this time we space pregnancy by using contraceptive methods, we never share bed with our children and no worry about urine and stool soiling, thus I share bed with my husband and give time to each other. Now we are in peace even we fail to have dinner at night. He never looks outside and never wants to have another marriage as long as he gets what he need from me. We live now lovely and smooth life. Our love has heightened. This is in line with the saying of our elders in handling dispute between a husband and wife. After trying their best, they final say that let the night and bed would mediate the issue (መኝታ ያስታርቃቸሁ ይላሉ). This is clear that the neither blanket nor the bed speaks to mediate the dispute but…. creates environment to know each other.

Since we have started using the service our feeling has improved for most women. I know that the peace of my family is largely contingent on the sexual relation we have. If I want to take care of my children, first we have to understand each other in this perspective.

Other discussants: yes there is no fear about that. We move out of our parents houses to him knowing what could happen. We approach each other necked, thus give each to the other to understand each other and fulfil ones desire. The bed knows what happens after that. There is nothing other than this. This is the core of our marital relation health and lives.

Dis # 3. In my case I had never changed bed even during a postpartum time. I just sleep under his hug totally submitting myself. There is nothing ashamed me. I am aware about it from my six grade science classes. I know what it means and what is sexuality and sex organs. In our culture when men feel unsatisfied by their wives sexual act, they tell the elders ironically saying that she never makes bed for me and never motivates me. Here it is not about making a bed but the issue is different. This is a clear message for a lady to warmly handle her husband otherwise he will go out in search of the one who can properly make a bed.

Dis #5. I do also agree on what have so far discussed. Except for the first week after birth, I never want to be alone in bed. I tell him the proper time to have relation after birth as the reproductive structure resume normality. It is well understood that when a husband and a wife are one person is to indicate that they share intimacy/love and affection but doesn’t mean they are brothers or sisters. Sexual relation is a good sign to express this situation. In case if I fail to treat him in this matter according to his desire, he refuses food for about ten days. What contraceptive service has created to us is that we have good time to care for ourselves and listen to our feeling now. Thus, we also express our feeling to our husbands, Sexual desire and health has become a matter of the two now.

*What is your experience about the health status of your family members in general and your? Most of the issues have been touched along the way in the above discussions but if anything you want to further explain.*

Dis # 7. My family members and all my children are well. If anything happens, I bring them first to the health post here. They manage them and if beyond their level, they refer to the health center. We receive new health education and vaccinate our children here. They are good and growing well.

Dis # 6. I have one child most of the time gets sick. He is now fourth grade student. The health personnel said him having bronchitis and gave him treatment. He becomes well when he gets treatment. During his sickness, he gets emaciated and appears critically ill.

*Let us take you to the final point of discussion. Tell us your general experience in summary about the life situation before and after contraceptive service use either positively or negatively*.

The will of God is always perfect. I have time now to participate in various activities. I fairly distribute my time for both domestic and outside activities, thus helped me to generate better revenue for my family. Our health status is very good when compared to our non- use time. We eat and drink well. Our garden gives us better yields. In general, contraceptive service is useful and made us feel happy. We have never faced big problems. Our lives have greatly changed.

*What is your desire and ambition for those who are not currently using this service in your community?*

In general we have very few not currently using the service. Many women are on the service. One seeing her neighbours lives and practices converted to use the service. We never keep ourselves quite even for one who is not using the service.

Thank you for sharing your deep experience by giving time for us.

1. **FGD in Gassara Kuwie Kebele**

Time started: 1:30PM and ended at 8:10 PM

Participants’ profile

| S.no | Age | Education | # of children ever born | Period of contraceptive use | Remark |
| --- | --- | --- | --- | --- | --- |
| 1 | 36 | 8 | 10 | 5 years |  |
| 2 | 28 | 5 | 3 | 1 |  |
| 3 | 30 | 2 | 6 | 8 |  |
| 4 | 30 | 5 | 4 | 1 |  |
| 5 | 34 | 2 | 4 | 3.5 |  |
| 6 | 32 | - | 4 | 3.5 |  |
| 7 | 35 | 7 | 5 | 11 |  |
| 8 | 30 | - | 2 | 4 |  |
| 9 | 25 | 3 | 3 | 5 |  |
| 10 | 30 | 7 | 5 | 9 |  |
| **mean** | **31** | **3.9** | **4.6** | **5** |  |

Q.1. *What are your lived experiences in terms of your general livelihood situations, social, economic and health perspective since you have been on contraceptive services? What change you have observed so far?*

Dis # 1. The change in our life can be expressed in this way. Before the service use we have experienced bearing children without space. The first before reaching the desired age, we bore other children. We catch hands of the older who hasn’t reached to walk and carry his/her young at our hug. Life after contraceptive use is different. Before we aim to have another child, we make sure that our older one has grown very well and able to go outside with cattle independently. We wait until the earlier child receives all possible care and grow well without frustration. We also wait until the older children grow to the extent they recognize what they need, they call mothers by their names and ask them to give what they need. Our children now grow fast and we are happy by seeing this. By the time when we were forced to bear children in closed gap, our body get emaciated and we easily get fatigued. Now we have got adequate rests and never over breastfeed ain out body don’t go emaciated like the former times. We look good and healthy. This is revealed that when we walk we put our legs on the ground with good forces. We have better energy to engage in various activities. During the time we gave frequent delivery and fed on breast milk without rest, our health status were devastated. We looked unhealthy and poorly nourished. Unlike the previous time we plan to bear children when the older child requests as” mom please buy and bring a brother/sister to me”. We mean, this time we are able to plan our pregnancy and child birth. We live comfortable this time (after contraceptive use).

*Tell is in detail, what area of livelihood improvement you have observed such as education, revenue and in general what you owe this time which you hadn’t previously?*

Dis #2. What I have used from the service is that I could discuss about the number of children we want to have with my husband and able to space pregnancy when we want to have. Thus, we have got time to accomplish our plans in time.

Dis #4. We have so many issues that differentiate our lives before and after contraceptive services. Before contraceptive use, we mostly experienced to have closely born children one after the other. I used to carry one on my lap and the other on my hands at the same time. But, contraceptive use has made life different. We are able to plan our pregnancy and child birth. Therefore, we could use our times properly whether for domestic work such as child care, washing our clothes and body, go out of house to generate some income, we are benefited. Our children grow charming and strong and we ourselves appear as strong and attractive to the sights of others. We have better strength and energy to involve in activities that require labour forces as compared our previous time. If we hadn’t seen its benefit, which do you think stay here for longer time looking for the service. It is an indication that we regularly visit the health post to receive our services means it is our priority. We are highly benefited as we reached to the condition of planning our pregnancies and births. We bear when we need and stop otherwise. This is all about the contraceptive services. Before the service both we and our children were weak and unproductive. But this time we perform what we plan. *If anyone wants to add more on what have been stated above?*

Dis #5. What I want to add on is that since we have started to use the service is that we have been able to handle our children and also able to make our husbands happy. We are also happy. Our non-contraceptive use time lives were full of misery and untidy. As already said above, repeated pregnancies and child birth have consumed our energy and weakened us. Lack of cleanliness due to leaking of babies’ urine and stool made us unclean and smell offensive.

Dis #7. Unlike our previous time, whether we go to market or to fetch water, fuel wood, we take time to properly accomplish what we are supposed to do in relaxed manner. The reason is that we have no urging young baby who could be discomforted or harmed in our absence for a while. During my absence my older children also support me in taking part of domestic responsibilities with light works. They do cook, boil coffee. Just to conclude, contraceptive service has greatly benefited me.

*In our discussion so far you say that contraceptive service has benefited you, improved your comfort and overall sounds wonderful. Do you have anything more to add in direction of your relation to others, anything that you got which has been your big dream etc…*

Dis # 3. During the time when I gave closed births, I was unable to offer my child with basic minimum. More specifically I was un able to offer my first two children better food or milk. After that I started receiving contraceptive services and took some times before becoming pregnant. During a non-pregnant time, I have owned cattle. When I gave both the third and fourth births, I could get milk at my house from my cows. Thus, I avoided worries related to what to provide for my children with owing these cows. Don’t you think that these are the contribution of contraceptive service use for me?

Dis # 7. Yes the rest of our idea is similar. We all are getting what we were deprived off before the services use. In my part, I have benefited enormously in area of caring for my children properly, maintaining their health status, providing them better cloths, keeping their cleanliness, sending them to school and upgrading their educational capacities. I recognize that this service has also given opportunity for me to strengthen bonds with my children. When I born year by year my mind was full of stress, no time to relax and thus show little interest on my children. But with contraceptive use I became able to postpone unplanned pregnancies and bring about planned births. Therefore, I have established strong bond with my children born after service use. I never annoy them now and then for simple things. I remember the time when I became tough for my child while he was begging me to keep on breast feeding. But this time he/she has got enough time to breast fed to the extent he/she needed it. On top of that I am able to care for them hygienically. They dress neat cloths, wash their body timely and eat well. I have also got time and capacity to plan for my children’s future life and development.

*What is your lived experience in moving outside your house for various social, individual, economic and political affairs? What experience you have with regard to receiving permission from your husband to do so or anything change as you compare with your previous time?*

Dis # 2. When I came to this appointment, I told my husband as I have issue here. He supports such meetings and my travel to the health post to receive contraceptive services. He never want me miss my appointment. If I show any reluctance, he warns me saying “your reluctance means to have more children; it is your responsibility, I am not accountable for this”. These days we never hide while coming to here as the former time. In past time when we wanted to come to health post for contraceptive service, we never made it obvious but cover it as we went to market holding some items at our hands. I do also share the information I receive here to my husband when I return to home. There is no hiding this day. When we inform them about the use, they become supportive in case if we need better food or care.

Dis # 8. Our husbands are supportive in all social affairs participations. When I come for contraceptive service appointment, he stays in house keeping it and caring for children. He also shows better care than before by supplying nutritious food items. In my part I have no problem. We do all in agreement and consensus. With regard to hosting gusts at our house, I handle smoothly. I go to market to buy and sell what very seem profitable and boost our family income. I do prepare food and drink and take to social gatherings and participate.

*It is common to have some supportive and some oppositions in various households. In this regard what is your lived experience as having different levels of support or oppositions?*

Dis # 9. In my part, I have no problem. Our country is in peace. I am housewife and do domestic work. Many od women in our kebele including me are not engaged in trade activities. Our major duty is domestic work. It was true that we were in great trouble before contraceptive services. This time we have no major problem.

Dis # 6. It is true and clear that our major duties are handling domestic responsibilities. We, the women are the prime responsible for these parts of duties. Sometimes there are dispute between our husbands in case of contraceptive use. But the good thing this time is we never stop using the service as a result of the dispute. I am housewife and never participate in trade activity. With regard to domestic work, I share with my children.

*What is your lived experience in sending your children to school in general and female children in particular as seen before and after contraceptive use?*

Dis # 10.We send our children to school. We do so as we understood that if we fail to send them to school, they will face the same as we had. We want to see the future through them. We don’t want to see our daughters suffering as we did and to some extend doing now. Many of our daughters are attending school and their attendance increase from time to time. In my part I have great dream to see my daughters’ capacity strengthened/reach higher level by education. I am committed to see in their life which I have missed. I missed as I was ignorant but I don’t want to repeat the same mistake up on my children.

*What is your experience in offering support in light of their education at home by letting them study? What is the level of support to both sons and daughters? Sometimes it is common in rural community as a daughter is expected to help her mother and frequently distracted. What is your experience in this regard?*

Dis #5. In my part consider both in similar manner. I send both male and female children to school.

Dis #2. I have different opinion in this regard. Our community do not treat males and females equally. A daughter is easily sent everywhere. She goes to fetch water, collect fire wood and where ever the mother wants to send her. When a mother moves out of her house, all the burdens are shouldered by her daughters. Sometimes females are considered as outsider. If one see that a female student drop out her school and marry, then they say to their respective daughters in school, you better remain in house until you move out by marriage.

Dis #4. Even if it is not uniform across all households in our community, unequal treatment between male and female children at their education exists. This doesn’t mean that there are no family equally treat their children equally. Some mothers are bottle necks for their daughters’ education. They undermine their education by considering that they end up in marriage. Many of our society do not value female education. Some parents are totally not willing to send their children to school by saying that what could I get out of your education. It is all about yourself they say. Many of children at school do all by their efforts. This is also true outside education as well. Female children are the one to feed at last. If good food get prepared, precedence is given to husband, sons and lastly to the females. After school sons go out to play ground and pass playing with their friends while females immediately enter to the kitchen to prepare food for other family members. Here you can see how differently they are being treated.

Dis # 8. This time female child also acts freely as compared to the former time. The work load for them has reduced. Sometimes the problem is from females themselves. For example, female circumcision is known to be harmful traditional practice. But when we inform them about the issue, they go outside the house with their peers and end up in circumcision. Our problem is that they are influenced by their peers and their parents.

*What is your experience in relation to contraceptive service use and female education and treatment at household?*

Dis # 9. I was educated up to grade three before I have married. My fellow female classmates who have completed their education were employed and earn salary. Had I persuaded my education like them, I would have been employed and earned salary. I advise my daughters not to be like me but to be strong in their education. My small education helped me to take care of my children, maintain their cleanliness, health and myself as well. Contraceptive service use has enabled me get better time so that send my daughters to school.

Dis # 8. I send five of my children to school. I never differential treat them based on their gender. Though our parents hadn’t sent us to school, I send them to school. I feel comfortable by doing so. I know I missed many privileges due to the mere fact I was not educated. I was eager even to attend the adult education classes but not happened. I really feel bad about it and always regret. I think that if our parents were able to use contraceptive services and born children in planned manner likewise we do, we could have got educational opportunities. My ambition now for my self is if the kebele start the adult education, I want at least to identify numbers and some Amharic words so that to use cellphone to communicate with others. I know if I dream to the highest education level attainment, it doesn’t seem feasible as I am getting old.

*What is your lived experience in terms of your overall health status as you lived through contraceptive service use in comparison to your previous non-use time?*

- *Tell this in terms of how you feel physically, psychologically, emotionally, socially and sexually?*

Dis #3. I feel comfortable in all aspects now. With respect to sexual desire and feeling, I have no problem. Before contraceptive use I fear sexual relation with my husband for unwanted and mistimed pregnancy. Now nothing as such will frustrate me as I am able to protect unwanted pregnancy. We share all our feelings each other. I have a desire as a human being that has to be fulfilled. I feel joyful with regard to sexual intercourse this time. In order to satisfy each other, we discuss the sexual matter like any other household issue together. Contraceptive service has improved our health status. We better feed and take good care of ourselves in terms of hygiene and feeding. I have good feeling now as similar to the time I have married. When we were overwhelmed with frequent pregnancy and child births, we were not able to either satisfy our husbands or ourselves. As a result our husbands go for other wives. But, this time it is not a problem. You see, men want to eat better and need better care. They need respect and love. Now we are able to offer what they really desire. Overall since the time we have started using contraceptive service, we also started living better lives unlike the previous time. We reached to the level that we consider our personal health, other needs including sexual one and satisfy each other without limitations. I really thank our Lord for offering this opportunity and enabled us see the hope of life.

*What is your experience in visiting either to the health post or health center in seeking health services?*

Dis # 7. Yes we do. We visit to the health post when we are invited to attend health education and when we have appointment for our contraceptive services.

*What is your lived experience in terms of your children health, their growth and development including nutritional status?*

Dis # 6. This time our health status is fantastic. It was poor before the time we started using contraceptive methods. It was like one time one of our children get sick and the next time another one. The illness was rampant in our family but after contraceptive service use we have really see progress and improvement in our children health status. Pre-contraceptive use time was known for untidy life situation with poor nutrition situation and lack of time to properly care for our children. However, lives this time is different from the previous one as we have got relative emancipation from the burden of unregulated and mistimed pregnancies and child births and able to properly handle our children. We are thankful to the Lord and the government so as we saw this improvement (our children are growing well and their body catches proper weight to their age) in our eyes.

Thank you for giving as your time and shared detailed experiences in terms of the topic for discussion.

Abraham and Yamrot (research team).

1. **FGD from Dilarife kebele**

Boricha district

Participant’s profile

| S.no | Age | Education | # of children ever born | Period of contraceptive use | Remark |
| --- | --- | --- | --- | --- | --- |
| 1 | 25 | 7 | 2 | 3 years |  |
| 2 | 30 | 1 | 4 | 8 |  |
| 3 | 35 | - | 5 | 9 and half |  |
| 4 | 30 | - | 5 | 7 |  |
| 5 | 25 | - | 3 | 3 |  |
| 6 | 38 | 5 | 4 | 6 |  |
| 7 | 30 | - | 5 | 8 |  |
| 8 | 30 | - | 4 | 13 |  |
| **mean** | **30.38** | **1.63** | **4** | **7.2** |  |

*What is your lived experience in terms of overall livelihood and your health status since your have been on contraceptive service use? Tell these by comparing your lives before contraceptive use.*

Dis #1. In the past we never act like this. By itself this is great change for us. Starting from the time I am on contraceptive use, I became a woman with normal blood (ደሜ ረግቶ ሰው መሆን ችያለሁ). As I have started using contraceptive method, it enabled me to have charming baby. Now I hug attractive baby unlike the previous time. When I say attractive, before contraceptive use, I gave births in unplanned and un spaced manner that a baby comes immediately after the other and no time to properly breast feed and offer children their required nutrition. As a result, children born in such closely spaced manner became emaciated, sick and clumsy. It is due to this service as the government is committed to that I am able to see this improvement or change. By the time when contraceptive service started in our area, it charged 2.50 ETB. During that time we had no money to pay and unable to use the service. As a result we were giving many births despite our will even. Some of us against our desire have given many births. We repeatedly presented the matter to the woreda government and now the service is free and many of us have started using it again.

Before some time, we had no health post in this kebele. With the establishment of the health post, many women have started using the services. This service has given us many livelihood changes in our life. We have got big changes. *You have said that there are many changes in your life. That is good. Can you elaborate what specific life change you have attained as a result of contraceptive use?*  I have small plot of land which is also not fertile. What I have done since I started using contraceptive service is that I herd cattle, sell some and earn money out of them. You see, herding cattle is labour intensive. You have to prepare fodder for them. To do so, you have to have enough time. Contraceptive use has averted unwanted pregnancy for me and I am free to use this time for collecting fodder for my cattle. I also sent my children to school. One is grade 8, the next one is7 the third one is grade 6. I am also determined to send all my children to school as I know that unless they get educated they will burden for me. I have no enough land and my rudimentary agricultural activities cannot bear them in the future. Cognizant of the situation, I am investing my small income on my children’s education and they will be a good support for me in the future. By spacing pregnancies and child births, I have able to engage in income generating activities and boosted my family income. For example, I bought one cow by 4000.00 ETB and now using for milk production.

Dis #2. Due to our ignorance in the past, we used to bear children on yearly basis. For example, by the time when my first child was six months, I got pregnant the second one. I was forced to bear a child when the older one was unable to sit. I was badly harmed in such way in the past. I than the Lord and the government this time we are able to space pregnancy by using contraceptive services. By doing so we are able to properly handle our children. There is no fear of bearing children yearly basis and our child this time could never be worried about departing from his mother’s hug untimely. This time we also are able to keep the cleanliness of our children, properly feed them, show our love and affection to them. This time a new born child feed on breast and stop when he/she feel that was enough. There was no as such forced discontinuation like the previous time.

Dis # 3. Before the time of contraceptive service, we had badly affected like our mothers. Our lives were ruined because of unregulated pregnancies and child births. We were deformed/macerated and emaciated. But when the Lord saw our misery and put this wonderful thought in the mind of our government to serve us, we got many improvements in our livelihoods. With contraceptive use, we became able to space pregnancies unlike the former time. We are now cognizant about the burdens related to unregulated pregnancies, births and their untoward outcomes in our lives. Therefore, we use contraceptive services to avoid unwanted pregnancies and births thereby enjoy the fruits of these services. My pre-contraceptive reproductive life was expressed in such a way that I born children on yearly basis before the older child reaches to walk on his/her food, the subsequent child comes. But after I have started using contraceptive service, both my health and other livelihood in my house have been improved. You see, we ,the women, were out of “form” or “action” before contraceptive use but now able to find ourselves as part of society and able to care for ourselves. We are able to clean our body, our clothes and improved our appearance before other group of society and improved our confidence to mix /socialise with others in the community. This time, I have freedom to go out of my house to various places such as market to generate some income by involving in merchandized activities. This has also enabled me to herd cattle and earn money out of it. I can say that we have seen many improvements in our life

Dis #4. I too, have given many births before the inception of contraceptive services here around our vicinity. But, I have seen many improvements in my life since I have started using contraceptive method. The reason to say so is by comparing my previous life style with the current one. Before contraceptive service use, I was forced to have children in close gaps, thus I had no time and other resources to care both for my children and myself I proper manner. As the result all my body and clothes smell baby’s urine. The service has enabled me to get better time, energy and knowledge how to care for my children and me.

*You are mentioning about improvement in your livelihood but can you be more specific and how contraceptive use brought change in your livelihood and by what mechanism. Try to elaborate specific examples such as economic and others.*

Dis #5. In my case let me tell you how contraceptive service use helped me involve in income generating activities. This time I prepare food and sell for my customers in nearby. I generate some profit out of this and use to feed my children. Even though the profit is not surplus, my children eat well. Had I been pregnant these times, I would never be able to do so and earn some money, thus my children would have been exposed to food problems. Contraceptive service therefore, helped me to delay pregnancies and do the same for my family.

Dis #6. Everything has been nice since the time of the inception of contraceptive services. What I mean here is that I started using contraceptive service before the service has been organized in our kebele or this health post. I got information about the service when I took my sick child to one of the health institutions in nearby, the health professionals asked me how many children I had that time. I told them that I have six children. And they again asked my age. I did tell my age at the same time. I said to them that, yes you are right, I am very young to have these numbers of children but as I have no education and awareness about what to be done, I ended up on this. Then, the health professionals said that that number of children is too many for my age and guided me to take contraceptive services. Then I started to use contraceptive service in two months’ time after my six child birth. They put me on depo-provera injection at that time. Since that time on ward I have bountiful peace in my lives. Before contraceptive service use, I was like a prisoned person. I couldn’t go where ever I want like my friends. But now I do whatever I plan and go where ever I want to go. In the past I had no time and opportunity even to visit my parents as I was totally occupied caring for my young children. Now the situation is different. I can stay for more time visiting my parents as my children are now big enough and can care for each other. Thanks to the government which has brought this opportunity to our doors, our lives have improved. In the past when we bear many children, we couldn’t afford for better bedding material. We lay on the dry leaves of false banana’ inset’ (hashshucho), that scratched our body and lacerated us. Added up with the foully smell of young baby’s urine drip, the discomforts were unbearable. Therefore, spacing pregnancy and child birth means getting freedom and rest from messy conditions like stated above. With regard to labour involvement, unlike the previous time I am directly involving in labour work that generates money for me. For example, I contract in mass ‘injera’ making for various social ceremonies like wedding, big meetings and other social gatherings in my community. I, then, use this money for my family affairs.

Dis #7. I also have given two births before contraceptive use. When I see the health status of children born before and after contraceptive use, they have visible differences in their growth and body makeups. I have also observed my health status before and after contraceptive use. My health status after contraceptive use is better than the previous non-use status. This time I have better awareness about various types of contraceptive methods such as pills, injectable and implanon. The health extension workers regularly advices me to use whatever method out of the available ones best suit my situation. If one method become uncomfortable or develop unbearable side effect, I know I can switch to other type and use. Therefore, I have no big worry about unplanned pregnancies and able to participate in income generating activities other than domestic responsibilities. I merchandise coffee and earn some money. I bought sheep, goats and chicken, fatten them and sell to get money. This time we are building big house and I have given some money I got in such process to my husband for construction expenses.

These all achievements are due to the unprecedented government support and putting women’s need as its priority agenda. It has established health extension program and its wide health packages including contraceptive methods. Consequently, we use the service at our kebele without much delay and planned the number and time of child birth. Accordingly, our lives have been improved.

*What is your lived experience toward education and female children education in connection to contraceptive use? What is your perception about accessibility of education at time of your parent’ school age and what scenario could you have been anticipated?*

Dis #6. I have only one daughter. I was keen to see the fruits of her education and really supportive and courageous for her schooling. I closely followed her school attendance as well as behaviour. I used to ask daily about her school performance and with whom she goes to school and comes back home. These all efforts were not to see her premature school dropout and early marriage. She attended her schooling up to grade seven with many attempts of abductions for marriage. Finally, she ended in early marriage as I feared earlier. However, she had maintained my grace and brought a dowry of 8000.00 ETB. With regard to her schooling, she says she would continue after having one child. Her husband has also promised to do so by the time of marriage arrangement. Whatever the case, she has married in right way and kept up my grace in the community. It would have been disgrace for me if she had given birth without formal marriage. I feel proud that she has formally and gracefully married. *Please further explain for me that about female education. Do you mean that female education has to come only to the level of marriage? Or until brings handful amount of dowry?*

Dis # 4. I have two daughters. One of my daughters is attending her school. What I tell her is to proceed in her education being focussed. I know that there are many obstacles for female education in our society. I follow my daughter based on the knowledge I have obtained from various health education offered for us. Now a day many organizations in our community teach about the importance of female education. I also have attended educational session on harmful traditional practices including female genital cutting. Based on the education, now we are convinced not to let our daughters get cut their genitalia. But the challenges come from the side of our daughters. Some girls decide by themselves and want to be mutilated. I also advise my daughter even in this regard.

- *It is good to express challenges about female education but could you be focused more on education in general and female education in particular. Give details of your lived experience here.* *What are factors affecting quality and school attendance for females most importantly by the time when her mother give birth.*

All the participants in one word said the female child drop out her school when her mother gives birth.

Dis # 2. Both I and my husband have strong stand towards female education. We meticulously follow her educational performance. As I have mentioned earlier, I have used contraceptive method for eight years. I properly used my time to care for my daughter. She has completed her secondary education and obtained good result that would let her attend college education. We were curious to see her result and wanted to send to college with strong ambition. Both her father and I were keen to see her graduated from college and get employed and earn money. We were expecting her that she would also help us. However, she was cheated by her peers and married this year. I was greatly irritated by the incident (her marriage) without our knowledge. I tried to end the marriage but some people influenced me not to do so as once she has entered there. We were unwilling to accept the dowry from her husband to indicate that we were in disagreement of the marriage. We said to him instead of presenting the dowry for us let you make our daughter to continue her college education. We want her finishing her college education instead of receiving the dowry money for us. I have two more female children whom I send to school.

Dis #8. I have great interest to teach my children but their father is reluctant. One of my children is grade 10 and the other one is grade 9. The second one has dropped out this year from grade 9 as his father refused to buy exercise books for him. I try my level in their education and provide advice on the importance of education. My first child wanted to go for military service after completing his secondary school for the fact that his father couldn’t pay for his college education but through continuous advice I convinced him not to go. I have two daughters as well. One has marries and the other one is doing her schooling. The problem is my husband is not cooperative.

- *Some of you have said that you are doing close follow up on your female children. What do your daughters feel about such follow up and what did you feel in the past when your parents did the same on you?*

Dis #1. I prefer advice to strict follow up. When the control is too tight, young girls develop heritage up on their parents and by large wants to misbehave in any occasion they get. In my part say that let the Lord give them thoughtful mind and humble heart to follow what is acceptable and what is wrong. If I want to closely follow her, I am single person limited in time and space. She can yes to please me for face value but can do whatever looks good for her. Therefore, instead of doing tight control it is good to leave for the lord to make them righteous.

- *What is your experience about girls in your family as in some community it is common to say let the girl child and dead body leave the house urgently (timely). And how this affects female education? How can we relate this with having many births taking into account the time of your mothers?*

All the participants exclaimed that it is true that having many children forces parents to think in such way and undermine the status of female children.

Dis # 5. Yes , in the past for two reasons (either total absence of contraceptive service or lack of proper information about it) our parent gave births for more than 10 to 12 children. During that time they never encouraged to send their female children to school as they want her to help in domestic work. They said what education as we are being overburdened here with nonstop pregnancies and child births. They pulled us back from our education to care for younger children and help in other domestic work. They have put big scar on our lives. My live history is typically like this. I was enthusiastic to attend school but did never get approval from my parents. By the time when I asked them in order to allow me to attend school, their replies were contrary. I can say we and our children at this time are fortunate enough to get contraceptive services. I am able at least to send them to school and partly improve my livelihood through contraceptive service use. This generation is lucky as we encourage them to go to school by handling domestic matter by ourselves due to the contribution of contraceptive service. You see, this time we can postpone pregnancy, get adequate time to involve in various economic, social, religious and other affairs. But during our parent’s time the situation was different. They hadn’t had such services and couldn’t space pregnancies or child births and trapped in domestic work burdens including massive unregulated children care. They live miserable and messy lives. They hardly thought of themselves, no time to see themselves and appeared untidy, unclean and poor. That was why they were forced (በዚያ ብስጭት) to say their girls let leave my house and push for early marriage. They believed that a girl should marry early. No one would challenge them why they said so as they were in tension. They said so as they lived uncomfortable lives almost all their lives.

*What is your lived experience with regard to your health status as you lived in contraceptive use taking into consideration your overall health status (physical, mental social sexual etc…)?*

Dis # 4. With regard to sexual health and expression of sexual feeling, in most instances in our society the issue is considered mysterious. Everybody knows about it but doesn’t want to express it openly as it is considered a taboo. However, it is inevitable for us to engage in it as we cannot live without it. Everybody laughed by this speech. My sexual practice with my husband has taken various forms. When I was young and no big children, I used to have sex without reservation but this time my children grown older we have to look for safe time and place.

*Tell your experience with regard to sexual life as you have started using contraceptive service. What contribution has the contraceptive service offered in easing sexual life?*

Dis #3. My sexual desire has greatly heightened since I have started using it. Now I am on loop. I guess, loop has created uncontrollable desire as I feel. In the past I have no as such big desire and I never though whether I had such feeling but I don’t know what happened this day, I have heightened desire. I say that life with it is meaningless.

Dis # 7. Before contraceptive service availability and its use, we hated male. We had no good environment to think about what sexual feeling is. Our lives were tapped by many domestic work burden and demanding children care. As I said, we were not in position to care for ourselves and undermined our personhood. Therefore, we departed ourselves from our husbands. We lived in full of conflicting situation as our husbands urged to have sex but we denied their requests. As a result husbands were forced to look to other wives to feel their desire. The situation is now different. We use contraceptive service and share bedding with our husbands. We have no fear of unwanted pregnancy that hampers us from having sex. I am convinced to share my time with my husband in this affair. I don’t want to annoy him and to hide my feeling this time like the previous time. We do relations in agreement and lead peaceful life. *You said sexual issue is secret but you laugh when others talk, why?* Yes, I still say it is secret. Isn’t it? Hahaaaaaa…..

Dis #1. My sexual life is mostly similar as what have already stated. My Husband ( I call him my brother), has never married to other woman than me. I have never been departed from him. We have good love and peace in our lives. Now our children have grown and getting bigger. Thus, we have separate bedroom. We live one for the other. I accept all his requests and he too for me. We share each other’s feeling openly. We obey one another’s request and host the desire of each other. We deeply express our loves and does sexual intercourse in a manner one satisfy the other without reservation. We clearly understood sexual health is one of the important affairs for the family cohesiveness. If partners exhibit sexual disharmony, marital breakage will follow. Therefore, we live descent and lovely lives. These are the benefits we got from contraceptive use.

Dis # 8. Thanks to the government and my Almighty Lord for accessing this service. You know, if I say no for sex to my husband, the shortcoming is for me. He will easily go to another woman but I remain alone. When I say no for sex with him despite reasonable conditions from my part, for him it is equivalent as I put knife between him and me. Men do not want to compromise with their sexual feeling. They want to get it as soon as they need it. No reason would be good response for them other than it. From our part, the women, mostly we pass it covertly but the feeling is there. The desire is there with us even my daughter. I don’t want to lose my husband due to my disobedience. With this recognition, I created good relation with my husband and acts in healthy way. *If you have more, let you add*. We all agree on the points raised above.

Dis # 6. My experience is that unlike the past time (none contraceptive use), this time I start using injectable method at 45 days post- delivery and start to share bed with my husband. There is no reason to have two different beds as I have no worry of getting pregnant soon. Such comfort and convenience for us is achieved through contraceptive use. Peace in my house is established because of this services and I feel comfortable. Having good relation has also contributed to my overall health status.

Dis #5. I have different idea in this regard. Sexual relation between partners should be based on mutual understanding. When I pass all the day without rest swing here and there, my husband passes his day in recreation, we never have similar feeling. I don’t want to do sex with him in such manner. I totally hate the relation. You see, you are initiated to kiss even your child when she or he looks charming and attractive to your eye. Therefore, it is difficult to internalize the one who doesn’t care for you. It is true that we , the women, in this community have little power to influence our men in such relation. I know that if a wife says no to sex, he never eat or drink from her hands and may tell to some elders saying that, my wife didn’t offer me food or drink. But, that is ironic. The issue is not food or drink but about sex. Some say that she never makes bed for me and let me lie on it. This is also another way of expressing the disharmony on sexual relation between the husband and wife.

- *What is your experience in getting balanced, adequate and proper food at your house? Tell us your experience by comparing your pre contraceptive time with the current one.*

Dis # 1. This time is full of challenges for women and their daughters. It is difficult to respond to this question. However, I can say it is relatively good for men. It is difficult to conclude whether it is good or bad but one obvious thing is contraceptive use has prevented us from further worsening the situation.

Dis #4. As I have mentioned the benefits of contraceptive use, it has also freed us from many problems related to unwanted and mistimed pregnancies and child births. Though life situation in our area is not simple, contraceptive use has created good situation for us to postpone pregnancy and plan child births. Had there no such service at this particular time, you can imagine what would happen to us.

- *What is your lived experience in visiting health services institution and if you do so why?*

Dis #3. Yes I do visit health institution for children vaccination, for contraceptive method use and occasionally when any family member has some illness. All other discussants showed agreement.

- *Any of you have experienced defined medical problem*

Dis # 7. Yes I had experienced spontaneous abortion of twin pregnancy. At that time health professional informed me having anemia due to massive bleeding and they helped in this regard.

*How do you comment contraceptive service use in relation to yours and your family health status?*

All the participants mentioned collectively on this point saying that they have benefited from contraceptive use in many ways including their health status and that of their family as compared to the none use time. We got time to care for ourselves and better time to breast feed our young babies. We got time to take our children to health institution for vaccination programs. Over all the benefits of contraceptive service is enormous in our health and other livelihood aspects.

We thank you for very nice discussion, valuable information and sharing your time with us. .

We too!

1. **Focus group discussion of Chama Hembecho kebele**,

Boloso Sore District

*Profile of the discussants*

| S.no | Age | Education | # of children ever born | Period of contraceptive use | Remark |
| --- | --- | --- | --- | --- | --- |
| 1 | 36 | 8 | 10 | 5 years |  |
| 2 | 30 | 5 | 3 | 1 |  |
| 3 | 35 | 2 | 6 | 8 |  |
| 4 | 30 | 5 | 4 | 1 |  |
| 5 | 30 | - | 4 | 3.5 |  |
| 6 | 35 | - | 4 | 3.5 |  |
| 7 | 35 | 7 | 5 | 11 |  |
| 8 | 30 | - | 2 | 4 |  |
| 9 | 20 | 3 | 3 | 5 |  |
| 10 | 30 | 7 | 5 | 9 |  |
| 11 | 35 | 5 | 4 | 5 |  |
| 12 | 35 | 6 | 7 | 6 |  |
| mean | 31.75 | 4 | 4.75 | 5.16 |  |

Moderators: 1. Abraham Alano , researcher and Bayush Moges, assistant

*What is it like to live through contraceptive use towards your status with the respect of your income status?*

- - - *What is your experience about economic status as to fulfil your basic need, your children and the family as a whole?*
    - *What is your lived experience like as far as you started to use contraceptives?*
    - *How you experience the situation in improving your income level at your individual and family level?*
    - *Learn the experiences as expressed by the women using as many probes as possible until information get saturated.*

Dis #1. Before the government introduce contraceptive services, we faced much harm. But after we started using contraceptive methods we have got many gains. Now we understand the difference in our livelihoods between before and after contraceptive use. I have experienced great change in my since I started using contraceptive method in 1999 E.C. Thanks be to my Almighty Lord, my life has greatly improved. Instead of getting pregnant every year, I got rest. When I gave births every year basis, I had no time to breast feed, provide balanced food and over all care. The subsequent pregnancies took place close and deprive necessary care to the older one. Due to such conditions, the children born these ways got sick frequently. I was emaciated as giving births yearly basis and keeping on breast feeding. Contraceptive service use has enabled to get freedom from all the problems stated above. From the time I have started using contraceptive service, I started to space pregnancy for at least five years. I got adequate time to properly handle my children, breast feed adequately and grow well. My health status has improved and gained strength. I reached to the state of deciding when and how to get pregnant in connection to my health and economic status. Therefore, I am comfortable with contraceptive method use. The other one is when I take my children’s’ condition into comparison, there is clear difference between children born before and after contraceptive use. *How do you explain the difference?* One of the points of differences is the duration of breast feeding. Children born after contraceptive use have got enough time to breast fed and are stronger than those before. The other difference is that those born after the service have got adequate supplementary diet as contraceptive service enabled spacing pregnancy and gave me time to earning income, buy foods and prepare it in time. They grew in normal pattern catching up to their normal age and weight. I also feel healthy after using the service. I feel strong and comfortable inside. My womb has got adequate rest and I really feel healthy.

Dis #2. We have seen significant benefit since we started using contraceptive methods. *What do you mean by benefits?* Benefits can be explained in many ways. First and for most, we are benefited by spacing/delaying pregnancy which we used to do on yearly basis before. By delaying pregnancies and child births, we have got to give better care for our children and take care of ourselves. *Please further explain these benefits in connection to your overall livelihood including your economic status.*

The improvement in our livelihood is related to avoiding unplanned pregnancy and getting time to spare for better child care. Instead of having children on yearly base delaying this to three and more years has created opportunities for us to rest and apportion our time and resource in wise manner. We feed our children for longer time than before. We are able to keep our hygiene both our children and ours. We provide our children better food than the previous time and feed for ourselves in well manner. *What do you mean by saying hygiene of yours and your children?*

When we say cleanliness/hygiene, we mean that when we bear children in close gaps, we have no time to wash properly both our children and our selves. As a result, we look dirty/untidy. When we space or delay pregnancy, we have time and resource to maintain our cleanliness.

Dis # 3. Before contraceptive use our time was occupied by pregnancies and child births which in turn deprived us from much personal and social participation. But through contraceptive use now we are able to send our children school. On top of sending our children to school, we also have planned to continue our schooling which we have dropped long back. Among the factors created conducive conditions for this is contraceptive service use. Now we are not only sending our children’s to school but also we support them in all activities that would help them be strong in their study. We have also got chance to participate in various income generating activities at individual level and some of us are employed outside home.

*How do you contraceptive use towards involving in trading and other income generating activities?*

Dis #4. When we have young child it is must for us to stay home caring for him/her. It is unthinkable to go to market leaving a young child at home. Not only that but we get pregnant before we adequately breast feed our early child. After we have started using contraceptive methods, we give better time for our children until they attain normal growth pattern and age, we got time to move outside our homes to market, involve in trading, increase our revenue. Contraceptive service use has enabled us to be clean and healthy.

Dis #5. Health extension workers have given as many health related information. With respect to my individual life experience; I gave my first birth on April 5 1987 E.C. I had nothing by the time when I gave that birth. I have nothing to give her to eat or to drink. My husband was the ex-soldier and we have no adequate resource. My daughter is pretty but due to our abject poverty we couldn’t give her basic requirements. Therefore, she was critically malnourished to the extent that her hair turns to be white as old person. While I was unable to feed my first daughter in the aforementioned critical situation, I got pregnant again suddenly in a year time. Thus I was forced to stop breast feeding my first child despite her deep desire to continue it. She was crying very much when I prohibited her from breast feeding. My second child came soon after.

After having the two births in such situation, I heard the availability of contraceptive services in Family Guidance Association of Ethiopia, Apposto Branch. I went there and in 1989 E.C. and started to use contraceptive service. I gave my third child birth in 1992 E.C. spacing for four years. I, then continued using contraceptive service up to 1997 where I gave birth to my forth and last child. After that I started using one of the long acting methods, the surgical implant. Since I started using this method, I have engaged in trading activities. I travel long ways “up to Areka” and buy items and sell in local markets. My last child is seven year and currently she is grade two.

*What is your life experience about contraceptive use and capability to involve in income generation at you own, providing decision and physically to move outside from your home in order to participate in social affairs, various meeting and religious affairs?*

Dis #6. As of the time contraceptive services have been incepted in our area, we, the women have greatly benefited. I can site some benefits out of them as follows:

- we are able to space pregnancies and child births;
- we got relief from burdens related to unplanned pregnancies and child births;
- Handle our children in better manner than the previous time, etc…

Our area is known for chronic food shortage; consequently our children were chronically malnourished formerly. We are trying to overcome such hardships involving in many activities. In this manner we send our children to school by attempting to fulfill their necessities. We lived in such challenging circumstances before we started using contraceptive services. Contraceptive service use have enabled us to delay unwanted or mistimed pregnancies and there by properly care for our children, helped us to provide better food as compared to previous time, enabled them (children) grow well, are among the benefits we obtained.

On top the above benefits, when I forecast for the future, my recent (last) child and the older children look not the same. *How the differences are experienced?* One of the differences is related to the duration of breast feeding. My children born before the time of contraceptive use were unable to fed breast milk adequately. They were forced to stop despite their urging desires to continue. On the other hand, my last child fed on breast milk for four complete years. They also differ in their growth mile stones. My last child grew up very fast and strong as compared to the older children. These are some of the benefits I could mention as observed in my life. What I anticipate for the future is that contraceptive service will further enable us to offer better care sustainably. It will enable us to give more attention to our children’s future career and competencies. Generally, in my experience and opinion, I conclude that contraceptive service use has created wonderful opportunity for our livelihood.

*What was your experience related to the bond between your children before and after contraceptive services?*

I had experienced unique bonding with my last child. I had better awareness about child care during the last pregnancy*.* The adequate time I had during my last birth relatively given me more time to spend and care for my last child. I devoted long time to breast fed her as a result we got chance to play each other. I cuddle her and she smiles facing me. By doing so, our relation was strengthened in comparison to others.

*Why you didn’t use the method previously as it had all these benefits?* I stayed out of use in past time as I had no awareness about the service. I started using the service as I got information about the service and its benefits. Not only to use for me alone but this time I promote the importance of the services to other non-user currently.

Dis #7. In my part since the time I have started using contraceptive service in first place it has enabled me to properly manage my children. In addition to that most of us who are using the service abled to engage in various income generating activities such as trading. We became well in health, social interaction, and psychological perspectives. Since the time of the service use, we obtained normal body weight; eat well, and able to send our children to school. More over contraceptive use has enabled me to better breast feed my children as it has postponed the subsequent pregnancy. My recent children have shown nice growth profile, easily catch-up normal growth pattern and over all look gorgeous.

*What is your feeling and expectation about if the contraceptive service had been available before 2o or 30 years in this manner?*

Dis #1. In order to explain the situation, it is good to compare the two situations. I mean our life before and after contraceptive service use. When comparing the life and growth pattern of children I born before contraceptive service and a child I born after the service, there is quite visible differences. My first two children were born in close gap and I couldn’t provide proper care for both. None of them were able to feed on breast milk for at least two years. When I got pregnant unknowingly for next time, people warned me not to give breast for my first child. Since my first child has not got proper care, he frequently became ill. My second child born in the manner I was challenged between caring for my first one. As a result, she also suffered similar burdens. She failed to attain normal growth pattern or bear normal body weight to her age. The conditions of my earlier children have greatly annoyed me and I have decided to take implannon for fear of the occurrence of subsequent pregnancy. Some people viewed me as if I was a mad, but I never given attention to what they said. I had enough challenges already. I was greatly challenged with my second child. Her health status was not good. I therefore, strongly determined to continue on contraceptive use. After I have started using the service, even my health status has improved. I am able to attain normal body weight. I have also no fear of pregnancy at this time. Therefore, had the service been in place in our area before 20 or 30 years means, our mothers could have been used and transmitted information for us at our early child hood. If we were informed about the service from our parents and community members lived that time, we would have never engaged in such miserable life related to unregulated fertility.

*What are your experiences about contraceptive use and engagement in various social, economic and political affairs in your locality?*

Dis #8. Yes we have quite experiences in these regards. As it has well explained earlier, our pre contraceptive lives were circumscribed by close pregnancies and child births. We had no enough time to look for other life affairs. Though some of us were knowledgeable about the other spheres of life, we couldn’t exercise as we had no spare time for such affairs. That life alienated us from many social affairs. We remained pregnant for one year and breast feeding and caring for the child the second year. So we had no time to go out of our houses either to market or other social gatherings. We were totally isolated from such affairs and limited ourselves in domestic activities. Our lives with contraceptive service are different now. We, the women deprived of many pleasant social and economic affairs involvement, this time we are able to participate in most activities. Moreover, we became advocates for the non-users to join us. Since contraceptive services have enabled as to get better time, now we go to market, buy and sell, generate some income and boost our income, participate in significant social and political affairs in our community, came to visibility in the community, mixed with them. We are able to access health institutions for the services we are desirous. The services have created chance for us to be role models in our community. We not only benefited for ourselves but pulling many women to the service as they observed our condition and motivated to use the services. Therefore, this time, our contraceptive service use has created a better community network that can be used for many other developmental activities. The service has enabled us to use our potential in better way. Our communication capacity has improved. I speak in meeting without any shyness now which was unthinkable before. I explain the benefits of contraceptive use in big gathering presenting my experiences as example. I say to them that see my life, God has brought this service to help me. When we start to talk to our neighbour about contraceptive service, it has also improved our relation with our neighbours. We share our experience and outcomes to our neighbours and convert many of them to the service use. In general, I can say that contraceptive service has transformed our status and acted as a vehicle to transform others.

Dis # 9. If we had this service some times in the past, we would never be troubled and challenged with life misery. We feel happy as we lived in this service for short time. If we had chances to be used the service in long past, I can imagine how much our lives could have been transformed. I surely say that had we used the service earlier than this time, we would grow, prosperous, and healthier (steps higher). We all here in the discussion and others left in their house but using the service have greatly benefits from the service. If you ask me what are the benefits, not only planning numbers of our children but we are able to engage ourselves in many outside home activities and generate incomes. Some of us have changed our house cover from grass roof to corrugated iron sheet. We have benefited by sending our children to school and able to buy new cloths, able to visit health institutions and access health services for ourselves and our children. On top of that, we are able to speak out to others about the benefits of contraceptive services. So it is clear that if we are this much satisfied with the benefits contraceptive use with our short time use, we would have been much better of this if we used much earlier. We could have prevented some of our life challenges, improved our health status further, and reduced some drawbacks. We would have been happier, more thankful to the government and the Lord. That doesn’t mean that I am reserved from giving thanks to my God and government. I know that even with this short time use I have seen many changes. At least my children were able to adequately breast feed and got enough food. I was not able to do this before contraceptive use. My former children were deprived of breast milk while they had strong desires and I was not able to feed them while they urgently require food. I left them empty stomach in house for search of food at least if I could drop in their mouth. I got relief from all such ugly circumstances now. This is clearly due to contraceptive service use. My health status has improved. I built better house. I am able to own cow in my house and now getting milk. My children are easily feeding milk from mu house. In the grace of the God and facilitation of the government, I arrived this stage and wish for better in the future.

*What is your experience related to education in your family including your and your children a live in the era of contraceptive?*

Dis # 2. Yes one of the many ways we got change in our lives is ability to send our children to school. Had there been adequate contraceptive services formerly, we ourselves would have been educated and respected like our educated sisters. We would have arrived at better career, and obtained better positions. We would have been posted at higher decision making positions and prestigious. We left a bit back warded as we were uneducated. With recognition of all the drawbacks in our lives, now we are committed in teaching our children. We attempt to push our children forward in the career in order to attain better positions which we couldn’t. We work very hard (በቁጭት እየሰራን እንገኛለን) on our children career with expectation that they will fill the gaps we had. *How do you relate contraceptive service use with female education?*

Dis #3. Contraceptive service use has enabled our family to get relief. By the time when we bear children in close gaps, it was impossible to send our children to school. It was particularly unthinkable to send female children to school in that time. There was great burden on the elder female child when her mother bears children year by year. She was the one to feed her mother, wash diapers of the newborn, responsible for other family members and look for food from outside. These all family responsibilities could not let her attend to school. When a mother uses contraceptive service and avoid unplanned or mistimed pregnancy, a female child gets better chance and relief from the burdens mentioned above. Consequently, she can attend her school. *Are there no female children in this kebele outside school?* There are some female children still outside school. At this time it is unlikely to say that all female children in the kebele are attending school.

*If we compare a woman currently using contraceptive services with non-user woman, who do you think have better chance of sending their female children to school?*

Dis # 3. It is clear that the woman who uses contraceptive service has better chance to send her female children to school. As mentioned above the reasons are clear.

Dis # 1. With regard to female education, I always regret that I could not attend school and progressed in education direction. Having this in my heart I am dedicated to educate my female children. I offer all necessary support for them. I also inform her to be in school despite and challenges. Contraceptive service use has given me better time to care for my female children, inform them about the challenges of early marriage and child births, how these affect female career. I advise my female children no to be tempted to early marriage as this would blur their future career. I encourage them to continue in educational ladder to the extent they can go and attain better positions and develop competencies. In the process of contraceptive use I not only got time to care for my children but developed better awareness how to treat my female children and understand the challenges of reproductive health and advise them timely.

*What is your lived experience in terms of your health status in the era your lived in contraceptive use?*

Dis #10. Ahe…..My health situation since the time of contraceptive use has greatly improved. Before contraceptive use I had many health problems such as headaches, abdominal cramps and feeling of discomfort in different parts of my body. I fee healthy now. *Tell your health experience in terms of attaining desirable body weight, feeding well and balanced diet*. I have used two types of contraceptive methods; the first one is injectable and now surgical implant. Since I have started using the service, both my health and my children have improved.

Dis # 11. I was badly harmed before I started using contraceptive methods. *What harms were that?* My child bearing history was like this. The first two children born in very close gaps and I got pregnant for the third time. The third pregnancy was turn to be twin. I had difficulty labour this time. I was really puzzled to care for them in many ways. I had no enough to feed them and care properly for them. Had I known the existence of contraceptive service earlier, I would have never faced such problems. I have started t use contraceptive service in short fast. Before that I got pregnant immediately after I gave birth to one of my female children. Not knowing that I was pregnant, I kept giving breast milk. Due to these both the one in the womb and the one who fed breast became emaciated. When I gave birth, the baby was underweight. Her elder child was died as I could not give her proper care. The new born was highly underweighted as the result she had difficulty to catch up and I was forced to take her to hospital and health center. Through the efforts and supports of the health professionals and nutritional supplement, she has been able to survive and celebrated her fourth year birth day. Immediately after her birth, I started to use contraceptive method and at least able to care for my child. Thus, now my child is able to play with her age children outside. She is growing well and try to please me at home. I was badly affected previously with repeated pregnancies, child births and related health and livelihood problems. However, I feel well now as I am on contraceptive service. *How many children do you want to have in the future?* I am not ready to have more children at this time. Let me first take care of these children, educate them, and provide them what they want to eat, drink, and wear. Once I have reached to the extent that my older children reached desired level, we can plan to have addition. You see we cannot say no or enough to the number of children. However, I take care not to be trapped like the former time. Having many children in close gap create many problems both for my children and my health.

*What is your lived experience in terms of your relation to your husband in sexual and reproductive life and romance since you have been on contraceptive services?*

Dis # 3. When a woman is in perperium (post -delivery), her body, particularly near her chest and breast get dirty. Child’s urine contaminate our clothes and the dripping of breast milk wet our chest and cloths We have critical shortage of water, soap and time to clean these. We feel that we are unhygienic, our body is not well built, and therefore, we have barely any feeling for sex. We neither be happy nor feel to have it. Neither our husbands get attracted h to us in such messy situations. In case if they show desire to have sexual relation, we never show sign of acceptance to them. Consequently, the attraction (love) between our husbands and us has greatly affected. Contraceptive service use has made us to maintain our hygiene and keep our body well. Now some of us look very young and attractive to our husbands. Now we are not only satisfy the sexual desire of our husbands but also overtly express our desire for sex as we are clean and neat.

Dis # 4. Before contraceptive use our lives were trapped in poverty thus we were not in position to think of what a sexual desire was mean. We were unable to keep our cleanliness, care for ourselves and never think of what we have to be for ourselves. After we have started using contraceptive service, we are able to understand that sexual relation with husband is not only to bear children but also to strengthen the marital relation and love by satisfying each other in sexual performances. Since I have good stand both in health and hygiene that can motivate me to sexual relation, I am able to manage my husband’s sexual desire. Both I and my husband are happy now.

Dis #5. My husband has two more wives. I am his first wife. He has married to the two more wives after me. When I think back why my husband had married to these two wives, I recognized that I couldn’t satisfy his sexual desire properly. I was emaciated due to repeated pregnancies and child births. I was overburdened and tiresome with uncountable domestic responsibilities and not in position to think of myself. We weren’t able to harmonize our sexual feeling discrepancies. In my part, I had many drawbacks: not able to feed adequately, unhygienic, was terrified with the miserable lives of my children. As a result I was not aroused sexual as he does. This has created disagreement between us. He had raised his eyes to other women outside as I was not giving him what he need from me. I also remember those days when I said to me, ‘who knows God will bring days that would favour me as well’. Surprisingly, now my husband came back to me, we established a wonderful romantic life and practice our sexual relation as if we were very young adults. This is because my health status and cleanliness has improved through contraceptive use. Now I have promoted as a respected wife for him. We lead smooth life this time. All those bud times have passed. To mention some: the time he said where to go from your dirty live, brought additional wife in my house as he had disgraced me. Thanks to my God, all that days had passed and new sun rose for me to see this bright day. My life has greatly transformed and I am renewed.

All other discussants have showed their agreement on this point with broken hearts and surprises.

*What are your lived experiences in relation to your children health status? Tell us your experiences by comparing the time before and after the contraceptive use.*

Dis# 2. Our children health status after we started using contraceptive service has descent outcomes as compared to our pre-contraceptive use time. To elaborate this, let’s begin from the status of breast feeding, children born after contraceptive use have got enough time to fed on breast milk. When a woman become pregnant on a planned manner by using contraceptive service, she has enough time to visit health institution to attend antenatal care and receive vaccination. This vaccination provides some degree of protection to the fetus during birth. As we have time we take our children to vaccinations that prevent them from many child hood sickness and deaths. To mention some of these vaccinations are against measles, neonatal tetanus, tuberculosis, etc…. When the health statuses of children have improved, the frequency for a mother visiting to health institution for treatment seeking has greatly reduced. Health post provides services for minor health problems and refers to higher level health institutions for difficult cases. In such a way the health statuses of our children have improved and mothers got relief. *How do you relate contraceptive use with children vaccinations?* Let me put this condition in this way. Before contraceptive use I born children year by year and no time to care for them. Not time to take them for get vaccination but even sick they are. Now I am free to go to market and buy and sell commodities and earn income by which I buy good food items for them. I have time to take them to health post easily in order to get vaccination.

Dis# 7. In real sense as of the time we are on contraceptive services, the health statuses of our children have greatly improved in many ways. We got better times to take them to health institutions for vaccination and treatment if they got sick. Before contraceptive use, I was unable to go out of my house easily thus I couldn’t get necessary materials for my family. I had no time to keep them clean and fulfill what they need. As I was given birth year by year, I was forced to stay home watching my children. Contraceptive service use has created additional chance for me to be exposed for the information how to handle children and what are the factors affecting their health. In the past when my children got sick, I was overwhelmed to carry out all what lay people told me. For example when my child got abdominal ache, they advise me to give juices of various plant leaves and I did it. Moreover they advised me to put aste (ጠባሳ) when they had abdominal ach and I did that too. When people came to do the aste, I held his hands and legs to refrain him from movement until the procedure get done. But, I was crying with him while my child cried. Now this is not the case. We immediately take our children to health institutions when they get sick. We cleared our minds from attempting unproven traditional practices on our children, we are able to take them to modern health services.

Dis # 5. As of the time I have started using contraceptive method, I am able to plan my pregnancy and child births. Planning pregnancy has made me to access antenatal care services from the health post. This in turn has made my fetus to grow well in my womb and paved ways for his prospective life. I received all necessary care including vaccination, which have also positive contribution to the health of the fetus. More specifically we have got better health services both for our children and ourselves as the time of the establishment of the health post in our kebele. We were largely troubled with our children born before the establishment of this health post moving here and there seeking health services. We also were forced them to take to medically unproven services and exposed them to unsanitary/unhygienic services. Some of our children have lost their lives and others remained disabled. Now we are grateful to both our God and our government for them to avail the health post in our kebele, we got relief and our children are growing well and healthy.

*What level of change have you got in terms of contraceptive service as the time of the health extension programs incepted here?*

Before the establishment of the health post in this kebele, few of us had accessed contraceptive service by walking long distance to the health center or hospital or they came once in a month time as an outreach program and served us. During that time we had no enough time to discuss any problems arising from the method use. But now as the health extension workers are living with us all the time, we bring minor problem even to share with them and get support and advice from them. Formerly we were easily terrified when we saw minor problems related to the contraceptive methods. We said to ourselves that wow!, are we receiving much trouble than the problems of pregnancy and badly terrified. Before the establishment of the health extension program, we were exposed to hunger, transport expenses and forced to stay long time outside our homes going to hospital or health center. We are able to use what method suit our condition here. We live relaxed and smooth life now.

Dis # 9. Before the establishment of the health post here, though we were cognizant of the existence of the service, we had no time to go far place to access it. Our husbands were not supportive to be absent from house for such long time; a day or half a day. But now the service is very close to us we never tell then where we are going and simple goo out as if we are going to the next neighbour and access it. Moreover it has created opportunity for us to choose among the available methods. In general, the health extension program has created wide chance for contraceptive service access for both us and to share our experience for the current non-user.

*What is your experience in terms of contraceptive service that you need to be improved for the future?*

Dis # 10. With regard to contraceptive service in this health post we have depo-provera, implannon and we go to the health center if we need loop. We don’t know methods other than these but you may know if any other methods exist other than this and that can be introduced to our health post. At the beginning of this discussion you have raised about the permanent contraceptive method. Where can we access this service from? We wish to have such permanent methods service here in our area. If services that can be accessed by men, they can discuss with their partner and access the services. This will improve the overall contraceptive service access and reduce burden on the women. Even among us there are some who may opt to the permanent method. In this regard I have different idea to what one of the discussants said earlier, saying ‘we would never say no for child in future’. I have enough children and don’t want to have more children, therefore want to go for the permanent method. I us I have strong desire to use the permanent method.
