# Supplementary material for: Women's perception about contraceptive use benefits towards empowerment: A phenomenological study in Southern Ethiopia
Source: PLoS One. 2018 Sep 13;13(9):e0203432. doi: 10.1371/journal.pone.0203432 (PMC6136733; doi:10.1371/journal.pone.0203432)
Supplement: Supplementary file 4 [file pone.0203432.s004.docx]

UNIVERSITY OF SASKATCHEWAN

Behavioural Research Ethics Board (Beh-

Certificate of Approval

PRINCIPAL INVESTIGATOR	DEPARTMENT	BEH#
Lori Hanson	Community Health and Epidemiology	13-189

INSTITUTION(S) WHERE RESEARCH WILL BE CONDUCTED
University of Saskatchewan

STUDENT RESEARCHER(S)
Abraham Alano Ali

FUNDER(S)
UNIVERSITY OF SASKATCHEWAN
HAWASSA UNIVERSITY

TITLE
Contraceptive Utilization and Related Benefits Toward Women's Health and Empowerment in Sidama Zone, South Nationalities and People's Region Ethiopia

ORIGINAL REVIEW DATE	APPROVAL ON	APPROVAL OF:	EXPIRY DATE
03-Jun-2013	25-Sep-2013	Application for Behavioural Research Ethics Review<br>Participant Consent Form<br>Key Informant Guide<br>Focus Group Guide<br>Individual in-depth interview guide<br>Acknowledgement of:<br>Institutional Review Board Approval Hawassa University<br>TCPS2 CORE Tutorial Certificate of Completion Abraham Alano	24-Sep-2014

Full Board Meeting ☐

Delegated Review ☒

CERTIFICATION

The University of Saskatchewan Behavioural Research Ethics Board has reviewed the above-named research project. The proposal was found to be acceptable on ethical grounds. The principal investigator has the responsibility for any other administrative or regulatory approvals that may pertain to this research project, and for ensuring that the authorized research is carried out according to the conditions outlined in the original protocol submitted for ethics review. This Certificate of Approval is valid for the above time period provided there is no change in experimental protocol or consent process or documents.

Any significant changes to your proposed method, or your consent and recruitment procedures should be reported to the Chair for Research Ethics Board consideration in advance of its implementation.

Please send all correspondence to:

Research Ethics Office
University of Saskatchewan
Box 5000 RPO University, 1602-110 Gymnasium Place
Saskatoon SK S7N 4J8
Telephone: (306) 966-2975 Fax: (306) 966-2069

PRINCIPAL INVESTIGATOR
Lori Hanson

DEPARTMENT
Community Health and Epidemiology

Beh #
13-189

ONGOING REVIEW REQUIREMENTS
In order to receive annual renewal, a status report must be submitted to the REB Chair for Board consideration within one month of the current expiry date each year the study remains open, and upon study completion. Please refer to the following website for further instructions: *http://www.usask.ca/research/ethics_review/*

Beth Bilson

Beth Bilson, Chair
University of Saskatchewan
Behavioural Research Ethics Board

Please send all correspondence to:

Ethics Office
University of Saskatchewan
Room 306 Kirk Hall, 117 Science Place
Saskatoon SK S7N 5C8
Telephone: (306) 966-2084 Fax: (306) 966-2069

ሀዋሳ ዩኒቨርሲቲ
ህክምናና ጤና ሳይንስ ኮሌጅ
የምርምር ስነ-ምግባር ገም ጋሚ
ቦርድ

HAWASSA UNIVERSITY
COLLEGE OF MEDICINE AND HEALTH SCIENCES
Institutional Review Board

ቁጥር IRB/175/05
Ref.No
ቀን 28/08/13
Date

Research Ethical Clearance

Name of Researcher/s: ***Abraham Alano, Lori Hanson,*** [illegible]

Topic of Proposal: ***Contraceptive utilization and related benefits toward women's health and empowerment in Sidama*** [illegible] ***Zone, South Nations, Nationalities and People's Region, Ethiopia.***

Dear Sir/Madam,

The Hawassa University, College of Medicine and Health Sciences Ethical Review Board has reviewed the aforementioned project proposal with special emphasis on the following points:

1. Are all principles considered?
   - 1.1. Respect for person: Yes ☑ No ☐
   - 1.2. Beneficence: Yes ☑ No ☐
   - 1.3. Justice: Yes ☑ No ☐
2. Are the objectives of the study ethically achievable? Yes ☑ No ☐
3. Are the proposed research methods ethically sound? Yes ☑ No ☐

Based on the above-mentioned ethical assessment the IRB has:

A. Approved the proposal for implementation ☑
B. Conditionally Approved ☐
C. Not Approved ☐

Best regards,

Techalew Shimelis W/Kiros
Institutional Review Board
Chairperson

ሀዋሳ ዩኒቨርሲቲ ህክምናና ጤና ሳይንስ ኮሌጅ Institutional Review Board — Hawassa University College of Medicine & Health Sciences

CC:

- Head, CMHS

☎ +046 820 92 90
Fax + 046 2208755
✉ 1560 Hawassa
